# Transforming colloidal Cs_4_PbBr_6_ nanocrystals with poly(maleic anhydride-*alt*-1-octadecene) into stable CsPbBr_3_ perovskite emitters through intermediate heterostructures†
†Electronic supplementary information (ESI) available: Experimental details and procedures, EDS-STEM data, ^1^H and ^1^H–^13^C HSQC NMR spectra and discussion, annotated XRD patterns, PLQY spectra, FTIR and NIR absorbance spectra, tests of PMAO reactivity with powders of bulk Cs_4_PbBr_6_ and amine-free Cs_4_PbBr_6_ NCs, stability tests of CsPbBr_3_/PMAO NCs, HRTEM images of Cs_4_PbBr_6_–CsPbBr_3_ heterostructures, low-resolution TEM size analysis, PL maps and spectra of Cs_4_PbBr_6_ NCs at 27 K, and time-resolved PL, micro-PL, and Raman spectra for the NC-PMAO blend (PDF). A video showing transformation of non-luminescent Cs_4_PbBr_6_ NCs into green-emitting CsPbBr_3_ NCs after addition of PMAO (MP4). See DOI: 10.1039/d0sc00738b.


**DOI:** 10.1039/d0sc00738b

**Published:** 2020-03-20

**Authors:** Dmitry Baranov, Gianvito Caputo, Luca Goldoni, Zhiya Dang, Riccardo Scarfiello, Luca De Trizio, Alberto Portone, Filippo Fabbri, Andrea Camposeo, Dario Pisignano, Liberato Manna

**Affiliations:** a Nanochemistry Department , Istituto Italiano di Tecnologia , Via Morego 30 , 16163 Genova , Italy . Email: dmitry.baranov@iit.it ; Email: liberato.manna@iit.it; b Analytical Chemistry Lab , Istituto Italiano di Tecnologia , Via Morego 30 , 16163 Genova , Italy; c CNR NANOTEC , Institute of Nanotechnology , c/o Campus Ecotecne , via Monteroni , 73100 Lecce , Italy; d NEST , Istituto Nanoscience-CNR , Piazza S. Silvestro 12 , I-56127 Pisa , Italy; e Dipartimento di Fisica “Enrico Fermi” , Università di Pisa , Largo Bruno Pontecorvo 3 , I-56127 Pisa , Italy

## Abstract

The preparation of strongly emissive CsPbBr_3_ perovskite nanocrystals with robust surface passivation is a challenge in the field of lead halide perovskite nanomaterials. We report an approach to prepare polymer-capped CsPbBr_3_ perovskite nanocrystals by reacting oleylammonium/oleate-capped Cs_4_PbBr_6_ nanocrystals with poly(maleic anhydride-*alt*-1-octadecene) (PMAO). PMAO contains succinic anhydride units that are reactive towards the oleylamine species present on the Cs_4_PbBr_6_ nanocrystals' surface and produces polysuccinamic acid, which, in turn, triggers the Cs_4_PbBr_6_ to CsPbBr_3_ conversion. The transformation occurs through the formation of Cs_4_PbBr_6_–CsPbBr_3_ heterostructures as intermediates, which are captured because of the mild reactivity of PMAO and are investigated by high-resolution electron microscopy. The Cs_4_PbBr_6_–CsPbBr_3_ heterostructures demonstrate a dual emission at cryogenic temperature with an indication of the energy transfer from Cs_4_PbBr_6_ to CsPbBr_3_. The fully-transformed CsPbBr_3_ NCs have high photoluminescence quantum yield and enhanced colloidal stability, which we attribute to the adhesion of polysuccinamic acid to the NC surface through its multiple functional groups in place of oleate and alkylammonium ligands. The PMAO-induced transformation of Cs_4_PbBr_6_ NCs opens up a strategy for the chemical modification of metal halide NCs initially passivated with nucleophilic amines.

## Abstract 2

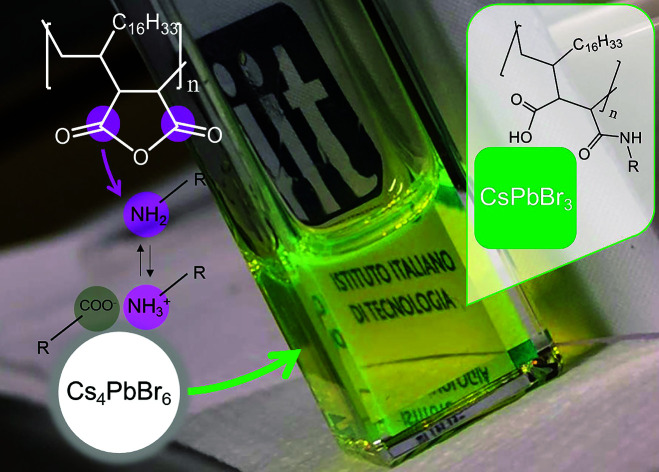
The challenge of making strongly emissive CsPbBr_3_ perovskite nanocrystals with a robust surface passivation is solved *via* Cs_4_PbBr_6_ → CsPbBr_3_ transformation triggered by a reaction of oleylamine ligand with poly(maleic anhydride-1-*alt*-octadecene).

## Introduction

Nanocrystals (NCs) of cesium lead halides have recently emerged as a class of semiconductor materials promising for light-emitting applications.^1^–^3^ The chemical reactivity of these NCs and the interconversion between the NCs of the two most studied bromides in this class, Cs_4_PbBr_6_ and CsPbBr_3_ perovskite, have been of interest since these NCs were first synthesized in the colloidal form.^4^–^8^ The Cs_4_PbBr_6_ → CsPbBr_3_ conversion, which can be triggered using various reagents (for example, Prussian blue,^9^ oleic acid,^10^ PbBr_2_,^7^,^11^ and water)^12^–^14^ is an interesting approach to prepare emissive CsPbBr_3_ NCs. For example, Yin's group exploited heterogeneous water-mediated CsBr extraction from Cs_4_PbBr_6_ NCs in hexane as a method for making luminescent CsPbBr_3_/SiO_2_ or CsPbBr_3_/Ta_2_O_5_ Janus-type heterostructures,^13^ and branched CsPbBr_3_ dodecapods.^15^ Despite several reports on Cs_4_PbBr_6_ → CsPbBr_3_ transformation at the nanoscale, the nanocrystal intermediates of this reaction and the surface passivation and stability of the resulting CsPbBr_3_ NCs have not been investigated.

Designing the Cs_4_PbBr_6_ → CsPbBr_3_ NC transformation in such a way that it delivers encapsulated CsPbBr_3_ NCs with an enhanced stability is a promising approach for exploiting the Cs_4_PbBr_6_ NC reactivity, as shown by the above mentioned studies of Yin's group.^13^ The use of an organic polymer instead of an inorganic oxide (*e.g.* SiO_2_ or Ta_2_O_5_) shell would yield a NC-polymer blend which can be drop-cast, spin-coated or electrospun, widening the range of available applications.^16^ More generally, polymer encapsulation of CsPbX_3_ perovskite NCs (X = Cl, Br, I, and their mixtures) is promising because it has been shown to enhance the shelf-time of NCs by providing enhanced stability against moisture and photodegradation.^17^ Interestingly, stability enhancement has been reported irrespective of whether polymer chains preserve the native CsPbX_3_ NC surface ligands as in the case of polystyrene^17^–^19^ and poly(styrene–ethylene–butylene–styrene),^17^ or whether the polymer adheres to the surface of CsPbX_3_ NCs as in the case of ammonium bromide-terminated polystyrene^20^ or poly(*n*-butyl methacrylate) modified with zwitterionic sulfobetaine or phosphorylcholine functional groups.^21^ Arguably, an ideal NC transformation of Cs_4_PbBr_6_ → CsPbBr_3_ in this context could be caused by a polymer which acts both as a reactant and a macromolecular surfactant,^20^ minimizing the number of reagents and preparatory steps involved in the process.

**Fig. 1:**
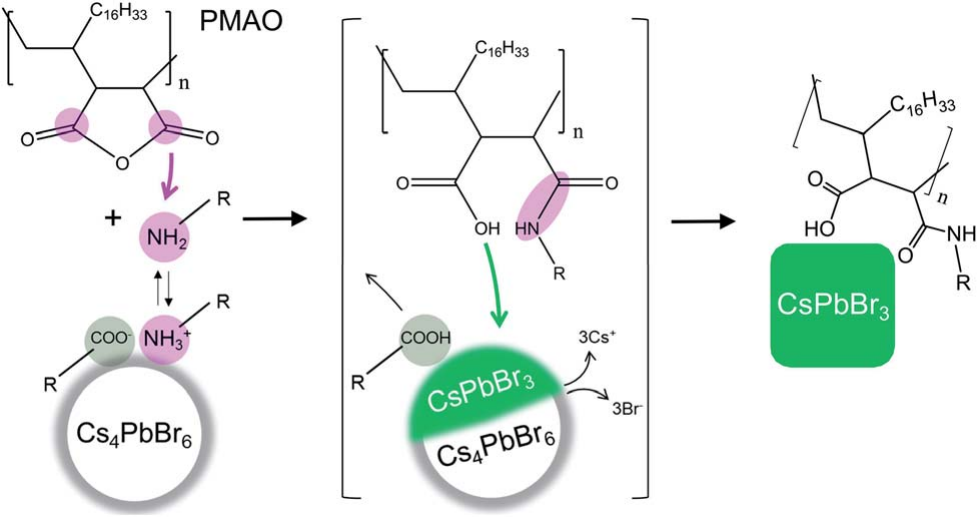
Schematic representation of the Cs_4_PbBr_6_ → CsPbBr_3_ NC transformation induced by PMAO. Oleylamine species from the NC surface react with the cyclic anhydride rings of PMAO, forming polysuccinamic acid. The removal of oleylamine-based and oleate ligands destabilizes the NC surface, and the formation of polysuccinamic acid increases the acidity of the medium, triggering the Cs4PbBr_6_ → CsPbBr_3_ transformation (see the text for the chemical equation). The resulting CsPbBr_3_ NCs are stabilized by the polysuccinamic acid in place of the original ligands.

In this work, we demonstrate that poly(maleic anhydride-1-*alt*-octadecene) (PMAO) can simultaneously trigger the Cs_4_PbBr_6_ → CsPbBr_3_ NC transformation and provide enhanced surface passivation to the resulting CsPbBr_3_ NCs. PMAO is a widely available co-polymer of 1-octadecene and maleic anhydride and has been extensively used for the surface functionalization of NCs.^22^–^24^ In our experiments, upon mixing PMAO with oleylammonium/oleate-capped Cs_4_PbBr_6_ NCs, the cyclic anhydride groups of PMAO react with oleylamine species, forming polysuccinamic acid (Fig. 1). Polysuccinamic acid destabilizes the NC surface by displacing both the amine and the oleate ligands and acidifies the reaction environment, thus triggering the formation of CsPbBr_3_ NCs (Fig. 1). The core chemistry of the NC transformation is summarized by the following chemical equation: Cs_4_PbBr_6_ + *n*RNH_2_ + (–R′(CHCO)_2_O–)_*n*_ → CsPbBr_3_ + (–R′(CHCOOH)(CHCONHR)–)_*n*_ + 3Cs^+^_(solvated)_ + 3Br^–^_(solvated)_, where R = oleyl, R′ = octadecenyl, and the ratio between oleylamine molecules and anhydride units is assumed to be 1 : 1 for simplicity. The extent of the transformation is tunable by varying the amount of added PMAO, enabling the investigation of the transformation intermediates, which consist of Cs_4_PbBr_6_–CsPbBr_3_ heterostructures. The fully-transformed CsPbBr_3_ NCs are bright emitters and retain their green emission for four weeks of storage under ambient conditions in air, even after one washing cycle with ethyl acetate (a solvent which typically causes the degradation of oleylammonium/oleate-capped CsPbBr_3_ NCs within hours or days). The increase in the stability of CsPbBr_3_ NCs synthesized from Cs_4_PbBr_6_ and PMAO is attributed to the adhesion of polysuccinamic acid to the NC surface through its multiple functional groups.

## Results and discussion

### Cs_4_PbBr_6_ NCs and their transformation with PMAO in solution

**Fig. 2:**
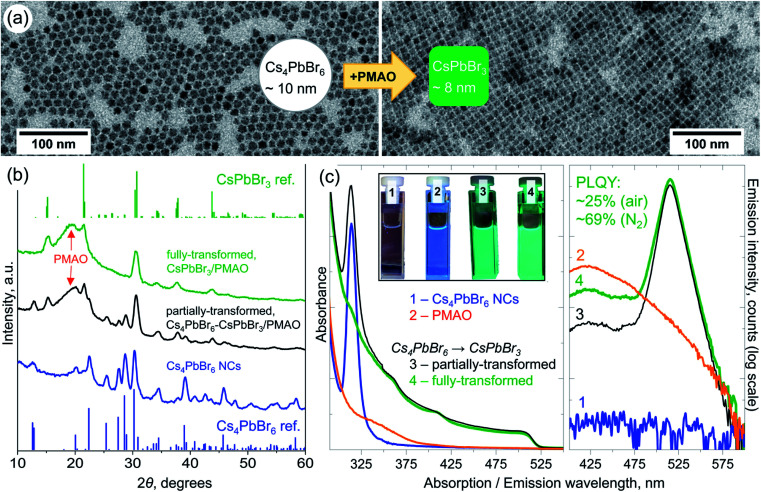
(a) Low-magnification TEM images of the initial Cs_4_PbBr_6_ NCs (average diameter 10 ± 1.5 nm) and fully-transformed CsPbBr_3_ NCs (average edge length 8 ± 0.4 nm) after their reaction with PMAO in toluene. (b) XRD patterns of the initial Cs_4_PbBr_6_ NCs and of the partially- and fully-transformed ones. Top and bottom stick patterns are those of the reference bulk compounds: rhombohedral Cs_4_PbBr_6_ (pattern ID 04-015-9683, ICSD code 162158)^27^ and orthorhombic CsPbBr_3_ (pattern ID 96-451-0746, COD code 4510745).^28^ The broad peak at ∼20° is due to PMAO. (c) Optical absorption (left panel) and emission (right panel) spectra of toluene solutions of initial Cs_4_PbBr_6_ NCs (blue curve), PMAO (orange curve), and partially- (black curve), and fully-transformed (green curve) NCs. The inset in the left panel shows photographs of the samples under excitation with a 365 nm lamp demonstrating visible green PL of partially- and fully-transformed NC samples.

The synthesis of the initial Cs_4_PbBr_6_ NCs was performed in air, *via* the hot injection of cesium oleate into the solution of lead(ii) bromide dissolved in a mixture of oleylamine and oleic acid in 1-octadecene,^7^ as detailed in Section S1 of the ESI.† The synthesis is similar to that of CsPbBr_3_ NCs,^5^ except that it is performed at a higher concentration of oleylamine and oleic acid with respect to lead ([oleylamine] : [oleic acid] : [PbBr_2_] ∼0.63 : 0.31 : 0.027  M). Such reaction conditions favor the formation of a Pb-poor Cs_4_PbBr_6_ phase over the CsPbBr_3_ phase, as detailed previously.^25^ The synthesis delivers batches of Cs-rich rhombohedral Cs_4_PbBr_6_ NCs with a narrow size distribution and an average diameter in the range from 10 to 16 nm (Fig. 2a, b, and S1–S5†). ^1^H and ^1^H–^13^C heteronuclear single quantum coherence (HSQC) nuclear magnetic resonance (NMR) experiments established that Cs_4_PbBr_6_ NCs are passivated by a mixture of oleylammonium oleate and neutral oleylamine with a ligand ratio of ∼3 : 2 between (oleylamine + oleylammonium) : oleate species (see Section S3 and Fig. S6–S9 of the ESI for details†).

These Cs_4_PbBr_6_ NCs can react with PMAO in toluene, fully or partially transforming into CsPbBr_3_ NCs, depending on the amount of the added polymer (Fig. 2c). The reaction of Cs_4_PbBr_6_ NCs with PMAO typically starts within a few minutes after the addition of PMAO at room temperature (Movie S1 and Fig. S10†) and can be accelerated by mild heating of the reaction mixture (up to 80 °C). It is important to highlight here that heating up the NCs alone to 80 °C without the addition of PMAO does not trigger any transformation (Fig. S11†). The fully-transformed CsPbBr_3_ NCs have a narrow size distribution, as inferred from their self-organization into ordered close-packed monolayers on a carbon-coated TEM copper grid (Fig. 2a). The XRD patterns of the initial NCs, partially- and completely-transformed samples are shown in Fig. 2b. Following the transformation, the XRD reflections of the rhombohedral Cs_4_PbBr_6_ crystal structure gradually disappeared, and peaks characteristic of the orthorhombic CsPbBr_3_ perovskite phase emerged (Fig. 2b, see Fig. S12† for peak assignment). The progression of the reaction was monitored by steady-state UV-Vis absorption spectroscopy (Fig. 2c, left panel, Fig. S10†) in which the disappearance of the ∼314 nm peak characteristic of Cs_4_PbBr_6_^7^ and the appearance of the ∼510 nm band edge absorption of CsPbBr_3_ are evident. The transformation was also tracked by steady-state photoluminescence (PL) spectroscopy, through the appearance of a cyan emission (*λ*_max_ ∼475–480 nm) in the early stages of the reaction (Fig. S10†). The absolute PL quantum yield (PLQY) of the samples transformed in air was measured to be ∼19% (partially transformed), and ∼25% (fully transformed). On the other hand, when the transformation was performed under an inert atmosphere, the sample had a 69% PLQY (Fig. S13–S15†). Such a value is comparable to those reported for other Cs_4_PbBr_6_ → CsPbBr_3_ chemical transformations of NCs: 47% (ref. 7) and 62% (ref. 11) *via* the addition of solid PbBr_2_ at elevated temperatures, and 75% (ref. 12) upon reaction with H_2_O. The lower PLQY of the samples transformed in air is attributed to the presence of electron traps formed as a result of sample exposure to atmospheric O_2_. Similar results have been reported by Rodà *et al.* who observed PL dimming in oxygen-exposed CsPbBr_3_ nanocubes.^26^

### Rationalization of the observed reactivity between PMAO and Cs_4_PbBr_6_ NCs

PMAO is a copolymer of octadecene-1 and maleic anhydride, and it consists of repeating units composed of a saturated hydrocarbon chain and a cyclic succinic anhydride ring (Fig. 1). PMAO has a negligible reactivity towards inorganic salts such as Cs_4_PbBr_6_, as confirmed in a control experiment on finely ground powder of bulk Cs_4_PbBr_6_ (Fig. S16 and S17†). However, the succinic anhydride rings of PMAO feature acyl groups that are reactive towards nucleophilic reagents such as water and primary amines (yielding, in the latter case, either succinamic acid at room temperature^23^,^29^–^31^ or cyclic imides at high temperatures^31^–^33^). The presence of a significant amount of water as a potential reactant towards PMAO in the Cs_4_PbBr_6_ NC samples was ruled out based on FTIR and NIR characterization (Fig. S18 and S19†). On the other hand, the ligand shell of Cs_4_PbBr_6_ NCs contains partially-protonated oleylamine (Section S3 and Fig. S6–S9†). In analogy with the widely studied oleylammonium/oleate-capped CsPbBr_3_ NCs,^25^,^34^–^36^ the ligands on the surface of Cs_4_PbBr_6_ NCs are likely to exist in a dynamic equilibrium between neutral and protonated species (oleylamine and oleylammonium, respectively). Thus neutral oleylamine is always available in the NC solution. Neutral oleylamine is a nucleophile with a documented reactivity towards linear and cyclic anhydrides,^37^,^38^ and polymaleic anhydride derivatives.^39^,^40^ The reaction between neutral oleylamine and PMAO in the absence of NCs causes broadening of the vinyl hydrogen resonance of the oleyl chain in the ^1^H NMR spectrum due to the attachment of small oleylamine molecules to PMAO macromolecules (the specified *M*_w_ of PMAO is ∼30 000–50 000 g mol^–1^, which roughly corresponds to ∼80–150 succinic anhydride-octadecene subunits) (Fig. S20–S22†). The addition of neutral oleylamine to cyclic anhydride produces a succinamic acid derivative, as was confirmed by ^1^H and ^1^H–^13^C HSQC NMR in a control reaction (Fig. S23†). Therefore, the formation of polysuccinamic acid (Fig. 1) is expected upon mixing of PMAO with oleylammonium/oleate capped Cs_4_PbBr_6_ NCs. The key role of oleylamine species in the Cs_4_PbBr_6_ → CsPbBr_3_ NC transformation was further verified by a control reaction between PMAO and oleylamine-free Cs_4_PbBr_6_ NCs (synthesized with tri-*n*-octylphosphine oxide (TOPO) and oleic acid^41^). The Cs_4_PbBr_6_ NCs synthesized with TOPO and oleic acid were found to be unreactive towards PMAO (Fig. S27 and S28†).

In summary, the removal of oleylamine from the surface of Cs_4_PbBr_6_ NCs destabilizes them, while polysuccinamic acid acidifies the reaction environment. Surface destabilization and acidic environments are both general conditions that are known to cause the Cs_4_PbBr_6_ → CsPbBr_3_ transformation.^10^,^25^,^42^ The stoichiometry of the transformation is balanced by a nominal removal of 3 equivalents of CsBr from 1 equivalent of Cs_4_PbBr_6_, yet we have not experimentally detected crystalline CsBr by XRD or high-resolution TEM (HRTEM). This discrepancy is tentatively rationalized by solvation of Cs^+^ and Br^–^ ions by oleate and polysuccinamic acid species, similar to the previously reported dissociation of CsBr in dimethylformamide in the presence of the polyacrylic acid co-polymer.^43^ Eventually, our ^1^H NMR analysis also revealed that the final NCs were capped solely by polysuccinamic acid, indicating the displacement of both the oleate and amine/ammonium ligands from the NC surface upon transformation (see the discussion in Section S9 and Fig. S20–S26†).

### Enhanced stability of the CsPbBr_3_/PMAO NCs

The fully-transformed CsPbBr_3_ NCs formed an optically clear solution in toluene. These CsPbBr_3_ NCs possessed an enhanced stability compared to the polymer-free, ligand-capped CsPbBr_3_ NCs directly synthesized by following the cesium oleate/lead(ii) bromide route.^5^,^25^ Such enhanced stability was demonstrated by the fact that the NCs retained their green emission after four weeks of storage under ambient conditions in air (Fig. S29†), even after undergoing a washing cycle of precipitation/redispersion with ethyl acetate (Fig. S30 and S31†), while the polymer-free CsPbBr_3_ NCs aggregated within hours or days after undergoing a similar washing procedure. Another indicator of the increased stability is the observation that the CsPbBr_3_/PMAO NCs could be concentrated or diluted over ∼5 orders of magnitude range of concentrations, from ∼26 mg ml^–1^ to ∼1 × 10^–4^ mg ml^–1^, without any loss of optical transparency or PL emission (Fig. S32†). The increase in the stability of the fully-transformed CsPbBr_3_/PMAO NCs is in agreement with prior reports on CsPbBr_3_ NCs blended with PMAO^44^,^45^ or with the related dodecyl-grafted-poly(isobutylene-*alt*-maleic-anhydride).^46^ Our hypothesis is that the binding of polysuccinamic acid through its multiple functional groups to the NC surface, in place of the standard ligands used in the direct synthesis of CsPbBr_3_ NCs (as discussed above and in Section S9, Fig. S20–S26†), is the origin of this enhancement. To test this hypothesis, we compared the solvodynamic diameters of PMAO and CsPbBr_3_/PMAO NCs (washed once with ethyl acetate) determined by dynamic light scattering (∼1.7 ± 1.2 nm and ∼11.2 ± 0.9 nm, respectively, Fig. S33 and S34†) with the sizes of the inorganic CsPbBr_3_ cores from the TEM analysis of the same sample (∼7 nm edge length, Fig. S35†). The larger solvodynamic diameter of CsPbBr_3_/PMAO NCs in solution compared to the CsPbBr_3_ NC edge length from TEM is explained by the PMAO wrapping and NC tumbling in solution (the diagonal of a cube with a 7 nm edge length is ∼12 nm). The lack of a substantial increase in the solvodynamic diameter of CsPbBr_3_/PMAO NCs is interpreted as an indicator of PMAO wrapping around NCs, supporting the hypothesis about the origin of increased NC stability. In addition, the relatively small solvodynamic diameter of CsPbBr_3_/PMAO NCs indicates that PMAO molecules do not bind multiple NCs together.

### Cs_4_PbBr_6_–CsPbBr_3_ heterostructures

**Fig. 3:**
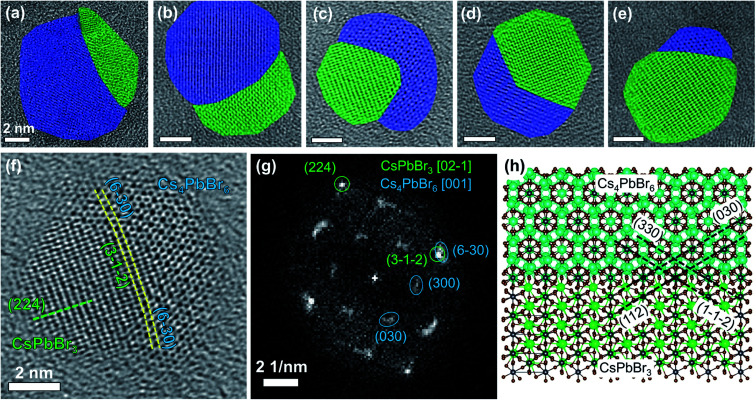
(a–e) HRTEM images of Cs_4_PbBr_6_–CsPbBr_3_ heterostructures formed upon partial conversion of Cs_4_PbBr_6_ NCs with PMAO (scale bars are 2 nm). Cs_4_PbBr_6_ domains are shaded in blue, and CsPbBr_3_ domains are shaded in green; (f) a magnified view of (c) and (g) the corresponding FFT image; and (h) ball-and-stick atomic model of the epitaxial interface built using VESTA software (ver. 3.4.6, the atoms are depicted as spheres with radii corresponding to 40% of actual atomic radii).^47^ Cs atoms in the model are colored in two different colors for clarity: in cyan for Cs_4_PbBr_6_ and green for CsPbBr_3_.

The Cs_4_PbBr_6_ → CsPbBr_3_ NC transformation with PMAO is relatively slow at room temperature. This enabled the observation of NC intermediates consisting of Cs_4_PbBr_6_–CsPbBr_3_ heterostructures (Fig. 3), which were investigated by HRTEM (Fig. 3a–e). In one of the partially-transformed samples we observed NCs with different degrees of conversion (Fig. 3a–e). The heterostructures displayed a variety of interfaces between Cs_4_PbBr_6_ and CsPbBr_3_, some adopting an epitaxial relationship, some not (analysis of the cases is shown in Fig. S36†). For example, the heterostructure shown in Fig. 3c, analyzed in detail in Fig. 3f–h, is characterized by an epitaxial relationship adopted by the two domains, as indicated by the overlap of the spots from the planes of the two crystal structures in fast Fourier transform (FFT, Fig. 3g) of the real space image. The <5% mismatch between the atomic spacing of the two domains 
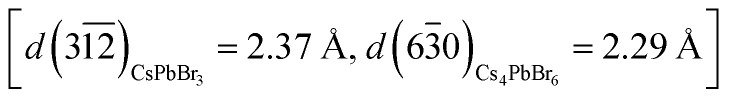
 leads to a slight bending of the planes, as labeled by the dashed lines in Fig. 3f. This bending also indicates that the atomic planes of Cs_4_PbBr_6_ domains on the two sides of the CsPbBr_3_ domain are rotated by a small angle. The rotation gives rise to extended diffraction spots in the FFT image, instead of single sharp spots that would otherwise appear for a single crystal. Considering an orthorhombic phase for CsPbBr_3_ (ICSD: 97851, *a* = 8.207 Å, *b* = 8.255 Å, *c* = 11.759 Å), the epitaxial relationship between the two domains can be described as follows: CsPbBr_3_ [021[combining macron]]‖Cs_4_PbBr_6_ [001], and CsPbBr_3_ (112)‖Cs_4_PbBr_6_ (030) (see Fig. 3h).

The low-magnification TEM images of the two NC samples were analyzed to quantify changes in the NC dimensions before and after the transformation (Fig. S37–S42†). For example, a sample of 10.1 nm ± 1.4 nm diameter Cs_4_PbBr_6_ NCs transformed into 8 nm ± 0.4 nm edge length CsPbBr_3_ NCs (Fig. 2a). The Scherrer analysis of the XRD patterns of the same sample before and after the transformation indicated a reduction in the crystallite size from 16.1 ± 1.8 nm to 12.5 ± 2.6 nm, in agreement with the TEM analysis (larger dimensions from XRD as compared to TEM are due to the differences between techniques and analyses). In another sample, 15.7 nm ± 2.6 nm Cs_4_PbBr_6_ NCs transformed into 12 nm ± 1.9 nm NCs (dimensions from TEM). If one assumes that such transformation does not proceed by dissolution–recrystallization, but simply by the gradual removal of CsBr from each individual spherical NC of Cs_4_PbBr_6_, converting it to a cube-shaped NC of CsPbBr_3_, then by volume contraction the resulting CsPbBr_3_ NCs should have an edge length of 6 nm in TEM (9.5 nm in the second sample), which is ∼2 nm smaller than the obtained value (Table S1†). Hence, dissolution–recrystallization processes should also play an important role in this transformation. A similar mechanism has been previously invoked to rationalize the inverse NC transformation (from CsPbBr_3_ to Cs_4_PbBr_6_).^8^,^10^

**Fig. 4:**
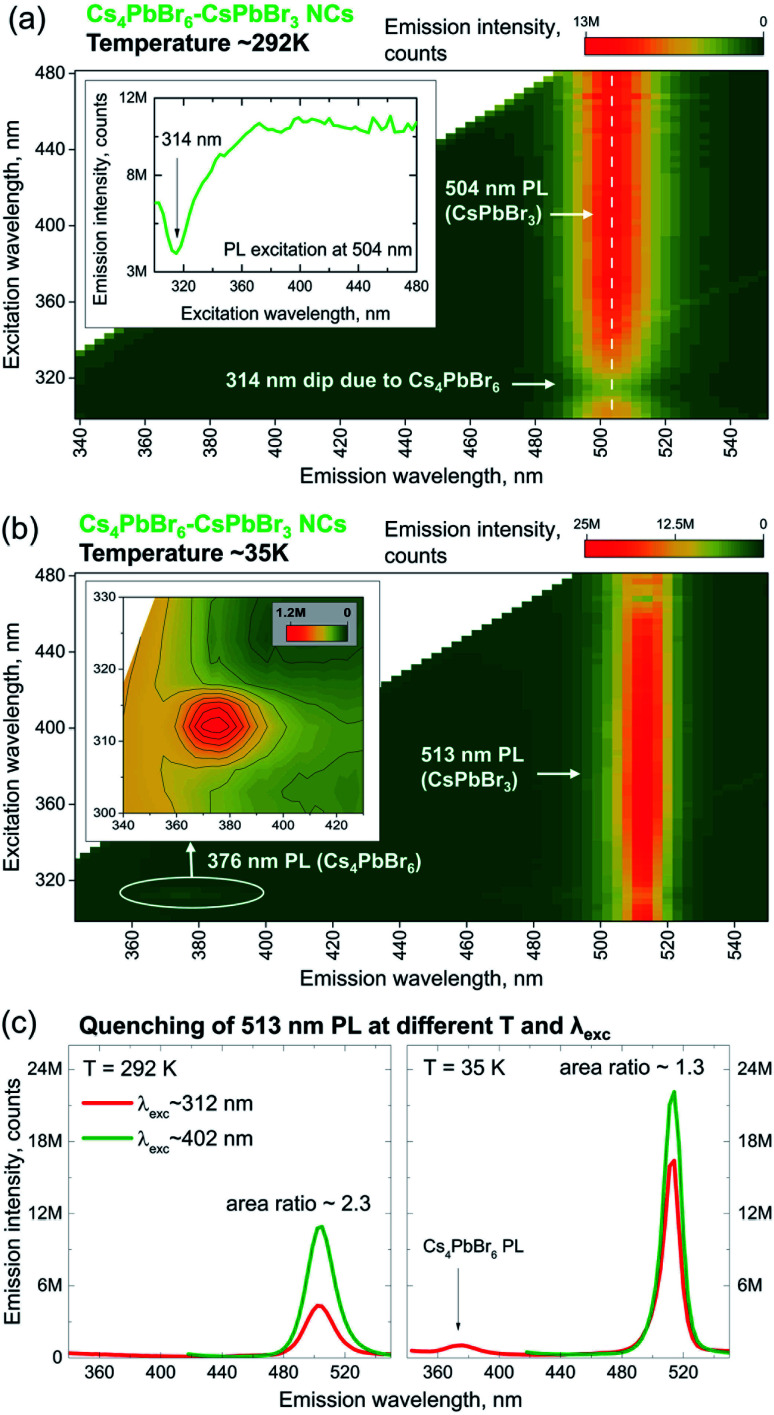
PL maps of (a) partially-transformed Cs_4_PbBr_6_–CsPbBr_3_ NCs at room temperature, and the inset shows the PL excitation spectrum at ∼504 nm (indicated by a white dashed line in the PL map); (b) partially-transformed Cs_4_PbBr_6_–CsPbBr_3_ NCs at ∼35 K, and the inset shows the low intensity region around 376 nm; (c) PL spectra of Cs_4_PbBr_6_–CsPbBr_3_ NCs at 292 K (left panel) and 35 K (right panel) collected under ∼312 nm (red curve) and ∼402 nm (green curve) excitation.

The PL of the partially-converted sample containing Cs_4_PbBr_6_–CsPbBr_3_ heterostructures was surveyed at room and cryogenic temperatures (see Section S14 of the ESI† for experimental details) because their optical properties are unknown to date. The results are presented in Fig. 4a and b as excitation-emission maps (PL maps). The room temperature (*T* ∼292 K) PL map of the partially-converted sample contains a single emission peak of CsPbBr_3_ at ∼504 nm (Fig. 4a). The CsPbBr_3_ emission has a broad PL excitation spectrum (inset in Fig. 4a) with a dip at ∼314 nm characteristic of Cs_4_PbBr_6_ absorption. Upon cooling to *T* ∼ 35 K, the PL map shows two emission peaks (Fig. 4b): an intense peak at ∼513 nm and a weak peak at ∼376 nm (inset in Fig. 4b). The ∼513 nm peak is an emission feature of CsPbBr_3_, red-shifted from ∼504 nm as a result of cooling.^48^,^49^ The ∼376 nm emission with narrow excitation at ∼313 nm is assigned to Cs_4_PbBr_6_ because it matches with previously reported cryogenic PL spectra of bulk Cs_4_PbBr_6_ (ref. 50) and Cs_4_PbBr_6_ aggregates in CsBr.^51^ This assignment was further confirmed by collecting the PL map of the as-synthesized Cs_4_PbBr_6_ NCs at *T* ∼ 27 K (Fig. S43†). At 27 K, the emission of the as-synthesized Cs_4_PbBr_6_ NCs is dominated by a peak at ∼376 nm surrounded by weaker features due to various electronic transitions in Pb^2+^ ions.^52^–^54^ The as-synthesized Cs_4_PbBr_6_ NCs are not emissive at room temperature and, besides the discussed ∼376 nm emission, are non-emissive up to the detection limit of 1600 nm when cooled (Fig. S44†).

The dual emission of partially-transformed NCs provides an opportunity to probe the energy transfer between Cs_4_PbBr_6_ and CsPbBr_3_. Fig. 4c shows a comparison between pairs of emission spectra for the partially-converted sample collected at two different temperatures (292 K and 35 K) and two different excitation energies: one matching with Cs_4_PbBr_6_ absorption (*λ*_exc_ ∼ 312 nm) and one below it (*λ*_exc_ ∼ 402 nm, only CsPbBr_3_ absorbs). At 292 K (Fig. 4c, left panel), only CsPbBr_3_ emits, regardless of excitation energy, and its emission is quenched by a factor of ∼2.3 after changing the excitation energy from ∼402 nm to ∼312 nm. This quenching is attributed to the attenuation of ∼312 nm excitation due to absorption by Cs_4_PbBr_6_ and an excitation-dependent PL efficiency.^55^ At 35 K, both materials emit, and the CsPbBr_3_ emission is quenched by a smaller factor of ∼1.3 (Fig. 4c, right panel). We can assign the lower quenching of CsPbBr_3_ emission at 35 K to the energy transfer from Cs_4_PbBr_6_, which indeed is favored due to the overlap between the emission of the donor (Cs_4_PbBr_6_) and the absorption of the acceptor (CsPbBr_3_). These initial observations make Cs_4_PbBr_6_–CsPbBr_3_ NCs a promising platform for future spectroscopic studies of the energy flow between lead halide perovskites and related compounds.

### Reactivity of Cs_4_PbBr_6_ NC samples with PMAO in drop-cast films

**Fig. 5:**
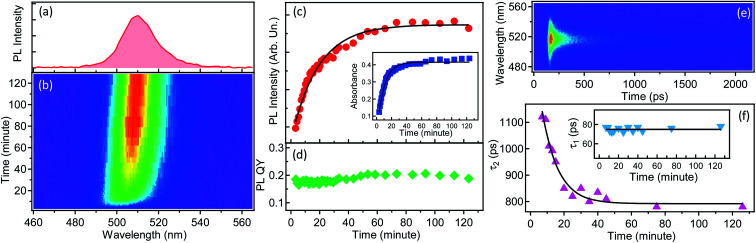
Tracking the Cs_4_PbBr_6_ → CsPbBr_3_ NC transformation in a drop-cast film by PL spectroscopy. (a) PL spectrum of the fully transformed CsPbBr_3_ NCs, peaking at ∼510 nm. (b) Spectrally-resolved temporal evolution of the PL spectrum on a minute scale, for ∼120 minutes. (c) Time evolution of the integrated PL intensity fitted with first-order kinetics (solid black line, time ∼10 min). The inset shows time-dependent absorbance at 405 nm over the course of the transformation. (d) Time-dependent PLQY of the drop-cast film. (e) PL intensity map showing the picosecond temporal behavior of the emission intensity of the drop-cast films. (f) Temporal evolution of the longer PL decay lifetime, *τ*_2_, over the NCs transformation. The continuous line is a fit to the data by first-order kinetics. The corresponding trend for the shorter component, *τ*_1_, is shown in the inset.

The reaction described above can also proceed inside a polymer film (as was confirmed by *in situ* Raman spectroscopy, see Fig. S45†), which makes its investigation relevant for the emerging application of blends between PMAO and oleylammonium/oleate-capped perovskite NCs in light-emitting diodes.^44^,^45^ From this point of view, the Cs_4_PbBr_6_ to CsPbBr_3_ transformation is an indicator of amine-anhydride reactivity, and its kinetics can be studied *in situ* by steady-state and time-resolved PL. Fig. 5 shows the results of the *in situ* PL measurements from a macroscopic area (∼2 mm excitation spot size) of the film made by quick drop-casting of a freshly prepared PMAO–Cs_4_PbBr_6_ NCs blend. Green PL develops within the first few minutes in the drop-cast film, and reaches a stable intensity and position (∼510 nm, full width at half maximum of 18 nm) after ∼2 hours (Fig. 5a and b), indicating the timescale of the complete conversion. Both the PL intensity and the absorbance of the film (at 405 nm, the wavelength of the CW laser used for excitation) increase over the course of the transformation, with a characteristic time constant of about ∼10 minutes (Fig. 5c). Similar kinetics were obtained by *in situ* micro-PL performed with a confocal fluorescence microscope (Fig. S46†), suggesting that the transformation proceeds uniformly across the blend. The PLQY in the film remains almost constant at ∼20% throughout the transformation, similar to the values measured in the solution (Fig. 5d).

The evolution of PL during the transformation was also monitored by *in situ* spectrally-resolved transient PL. The temporal PL decay is sub-ns and contains two main components, the shorter (∼70 ps) and longer (950 ps) ones (Fig. 5e and S47†). The shorter decay component varies little over the course of the transformation while the longer decay component decreases from ∼1.1 ns to ∼800 ps with a time constant of ∼10 minutes (Fig. 5f). The PL decay of the emitting NCs in the film is much shorter than that of the NCs in solution (∼4–5 ns), the polymer-free CsPbBr_3_ NCs^5^,^35^,^56^–^58^ (∼2–10 ns), and polymer-encapsulated single CsPbBr_3_ NCs (∼6 ns).^18^ It is definitely much shorter than that of MAPbBr_3_ NCs/polymer blends (>100 ns).^59^ The fast PL decay of CsPbBr_3_/PMAO NCs in the drop-cast film can be attributed to various possible causes, including: (i) the appearance of a new non-radiative carrier recombination channel, ascribable to oxygen molecules (as the samples were prepared in air) which act as traps for electrons;^26^ (ii) electron hopping between neighboring nanocrystals in the film;^60^ (iii) a more defective surface of NCs formed in films, due to reduced mobility of ions and molecules (preventing efficient passivation of surface sites in comparison to the solution case). The sub-ns PL decay of NCs in blends with PMAO, combined with a reasonable PLQY, should be of interest for applications in scintillators, where ultrafast and efficient emission is required for fast timing capability of imaging detectors.^61^,^62^

## Conclusions

Chemical transformation of colloidal Cs_4_PbBr_6_ NCs to perovskite CsPbBr_3_ NCs induced by the organic co-polymer PMAO is presented as a promising strategy to prepare stable and bright CsPbBr_3_ NC emitters. The PMAO reactivity towards oleylammonium/oleate-capped Cs_4_PbBr_6_ NCs favors an addition reaction of oleylamine ligands from the NC surface to the succinic anhydride groups of the polymer. This destabilizes the NCs and acidifies the reaction environment through the formation of polysuccinamic acid, a PMAO–oleylamine adduct, which binds to the surface of the NCs *in lieu* of the original ligands. These two factors – ligand replacement and *in situ* acid formation – drive the Cs_4_PbBr_6_ to CsPbBr_3_ NC transformation. The lower reactivity of PMAO, as compared to that of the previously reported reagents, enabled the investigation of Cs_4_PbBr_6_–CsPbBr_3_ intermediate heterostructures by HRTEM. The heterostructures feature a variety of epitaxial and non-epitaxial relationships between the two structurally dissimilar domains. At cryogenic temperature, Cs_4_PbBr_6_–CsPbBr_3_ NCs display dual emission at ∼376 nm and 513 nm with evidence of energy transfer from Cs_4_PbBr_6_ to CsPbBr_3_. The PMAO-induced transformation proceeds both in solutions and in drop-cast films, producing CsPbBr_3_ NCs with a narrow size distribution and attractive photoluminescence properties (up to 69% PLQY in solution and a sub-ns PL lifetime in the drop-cast films). The resulting CsPbBr_3_/PMAO NCs demonstrate enhanced stability by retaining their green emission for several weeks in air. The increased stability of CsPbBr_3_/PMAO NCs is attributed to the adhesion of polysuccinamic acid through its multiple functional groups to the NC surface. The PMAO-induced transformation of Cs_4_PbBr_6_ NCs opens up a general strategy for chemical modification of inorganic NCs passivated with nucleophilic amines.

## Author contributions

The manuscript was written through the contributions of all authors. All authors have given approval to the final version of the manuscript.

## Conflicts of interest

The authors declare no competing financial interest.

## Abbreviations

COD: Crystallography open database
EDS: Energy dispersive X-ray spectroscopy
FFT: Fast Fourier transform
FTIR: Fourier transform infrared spectroscopy
HRTEM: High resolution TEM
HSQC: Heteronuclear single quantum coherence
ICSD: Inorganic crystal structure database
NIR: Near infrared
NMR: Nuclear magnetic resonance
NC: Nanocrystal
PL: Photoluminescence
PMAO: Poly(maleic anhydride-*alt*-1-octadecene)
QY: Quantum yield
STEM: Scanning TEM
TEM: Transmission electron microscopy
XRD: X-ray diffraction

## Supplementary Material

Supplementary information

Supplementary movie

## References

[cit1] Kovalenko M. V., Protesescu L., Bodnarchuk M. I. (2017). Science.

[cit2] Akkerman Q. A., Rainò G., Kovalenko M. V., Manna L. (2018). Nat. Mater..

[cit3] Shamsi J., Urban A. S., Imran M., De Trizio L., Manna L. (2019). Chem. Rev..

[cit4] Nedelcu G., Protesescu L., Yakunin S., Bodnarchuk M. I., Grotevent M. J., Kovalenko M. V. (2015). Nano Lett..

[cit5] Protesescu L., Yakunin S., Bodnarchuk M. I., Krieg F., Caputo R., Hendon C. H., Yang R. X., Walsh A., Kovalenko M. V. (2015). Nano Lett..

[cit6] Akkerman Q. A., D'Innocenzo V., Accornero S., Scarpellini A., Petrozza A., Prato M., Manna L. (2015). J. Am. Chem. Soc..

[cit7] Akkerman Q. A., Park S., Radicchi E., Nunzi F., Mosconi E., De Angelis F., Brescia R., Rastogi P., Prato M., Manna L. (2017). Nano Lett..

[cit8] Liu Z., Bekenstein Y., Ye X., Nguyen S. C., Swabeck J., Zhang D., Lee S.-T., Yang P., Ma W., Alivisatos A. P. (2017). J. Am. Chem. Soc..

[cit9] Palazon F., Urso C., De Trizio L., Akkerman Q., Marras S., Locardi F., Nelli I., Ferretti M., Prato M., Manna L. (2017). ACS Energy Lett..

[cit10] Udayabhaskararao T., Houben L., Cohen H., Menahem M., Pinkas I., Avram L., Wolf T., Teitelboim A., Leskes M., Yaffe O., Oron D., Kazes M. (2018). Chem. Mater..

[cit11] Li Y., Huang H., Xiong Y., Kershaw S. V., Rogach A. L. (2018). CrystEngComm.

[cit12] Wu L., Hu H., Xu Y., Jiang S., Chen M., Zhong Q., Yang D., Liu Q., Zhao Y., Sun B., Zhang Q., Yin Y. (2017). Nano Lett..

[cit13] Hu H., Wu L., Tan Y., Zhong Q., Chen M., Qiu Y., Yang D., Sun B., Zhang Q., Yin Y. (2018). J. Am. Chem. Soc..

[cit14] Yang L., Wang T., Min Q., Liu B., Liu Z., Fan X., Qiu J., Xu X., Yu J., Yu X. (2019). ACS Omega.

[cit15] Chen M., Hu H., Tan Y., Yao N., Zhong Q., Sun B., Cao M., Zhang Q., Yin Y. (2018). Nano Energy.

[cit16] Wei Y., Cheng Z., Lin J. (2019). Chem. Soc. Rev..

[cit17] Raja S. N., Bekenstein Y., Koc M. A., Fischer S., Zhang D., Lin L., Ritchie R. O., Yang P., Alivisatos A. P. (2016). ACS Appl. Mater. Interfaces.

[cit18] Rainò G., Landuyt A., Krieg F., Bernasconi C., Ochsenbein S. T., Dirin D. N., Bodnarchuk M. I., Kovalenko M. V. (2019). Nano Lett..

[cit19] Wang Y., Zhu Y., Huang J., Cai J., Zhu J., Yang X., Shen J., Jiang H., Li C. (2016). J. Phys. Chem. Lett..

[cit20] Kim H., So S., Ribbe A., Liu Y., Hu W., Duzhko V. V., Hayward R. C., Emrick T. (2019). Chem. Commun..

[cit21] Kim H. Hight-Huf N. Kang J.-H. Bisnoff P. Sundararajan S. Thompson T. Barnes M. Hayward R. Emrick T. S., Angew. Chem., Int. Ed.10.1002/anie.201916492 , , accepted article . 10.1002/anie.201916492 32141215

[cit22] Pellegrino T., Manna L., Kudera S., Liedl T., Koktysh D., Rogach A. L., Keller S., Rädler J., Natile G., Parak W. J. (2004). Nano Lett..

[cit23] Lin C.-A. J., Sperling R. A., Li J. K., Yang T.-Y., Li P.-Y., Zanella M., Chang W. H., Parak W. J. (2008). Small.

[cit24] Di Corato R., Quarta A., Piacenza P., Ragusa A., Figuerola A., Buonsanti R., Cingolani R., Manna L., Pellegrino T. (2008). J. Mater. Chem..

[cit25] Almeida G., Goldoni L., Akkerman Q., Dang Z., Khan A. H., Marras S., Moreels I., Manna L. (2018). ACS Nano.

[cit26] Rodà C., Abdelhady A. L., Shamsi J., Lorenzon M., Pinchetti V., Gandini M., Meinardi F., Manna L., Brovelli S. (2019). Nanoscale.

[cit27] Velázquez M., Ferrier A., Péchev S., Gravereau P., Chaminade J.-P., Portier X., Moncorgé R. (2008). J. Cryst. Growth.

[cit28] Stoumpos C. C., Malliakas C. D., Peters J. A., Liu Z., Sebastian M., Im J., Chasapis T. C., Wibowo A. C., Chung D. Y., Freeman A. J., Wessels B. W., Kanatzidis M. G. (2013). Cryst. Growth Des..

[cit29] Kluger R., Hunt J. C. (1984). J. Am. Chem. Soc..

[cit30] Kluger R., Hunt J. C. (1989). J. Am. Chem. Soc..

[cit31] Jin Z., Du L., Zhang C., Sugiyama Y., Wang W., Palui G., Wang S., Mattoussi H. (2019). Bioconjugate Chem..

[cit32] Coleman L., Bork J., Dunn H. (1959). J. Org. Chem..

[cit33] Vermeesch I., Groeninckx G. (1994). J. Appl. Polym. Sci..

[cit34] De Roo J., Ibáñez M., Geiregat P., Nedelcu G., Walravens W., Maes J., Martins J. C., Van Driessche I., Kovalenko M. V., Hens Z. (2016). ACS Nano.

[cit35] Bodnarchuk M. I., Boehme S. C., ten Brinck S., Bernasconi C., Shynkarenko Y., Krieg F., Widmer R., Aeschlimann B., Günther D., Kovalenko M. V., Infante I. (2019). ACS Energy Lett..

[cit36] Quarta D., Imran M., Capodilupo A.-L., Petralanda U., van Beek B., De Angelis F., Manna L., Infante I., De Trizio L., Giansante C. (2019). J. Phys. Chem. Lett..

[cit37] Watanabe S., Kawahara H., Kuramochi T. (1991). J. Am. Oil Chem. Soc..

[cit38] Abbas M., Slugovc C. (2012). Monatsh. Chem..

[cit39] Percec S., Howe L., Li J., Bair S. (2012). J. Polym. Sci., Part A: Polym. Chem..

[cit40] Zhang C., Gao C., Gao F., Wang J., Zhang D., Wang Y., Xu D. (2014). Pet. Sci..

[cit41] Almeida G., Ashton O. J., Goldoni L., Maggioni D., Petralanda U., Mishra N., Akkerman Q. A., Infante I., Snaith H. J., Manna L. (2018). J. Am. Chem. Soc..

[cit42] Park S., An N. M., Almeida G., Palazon F., Spirito D., Krahne R., Dang Z., De Trizio L., Manna L. (2019). Nanoscale.

[cit43] Liu Y., Wang Z., Liang S., Li Z., Zhang M., Li H., Lin Z. (2019). Nano Lett..

[cit44] Meyns M., Perálvarez M., Heuer-Jungemann A., Hertog W., Ibáñez M., Nafria R., Genç A., Arbiol J., Kovalenko M. V., Carreras J., Cabot A., Kanaras A. G. (2016). ACS Appl. Mater. Interfaces.

[cit45] Wu H., Wang S., Cao F., Zhou J., Wu Q., Wang H., Li X., Yin L., Yang X. (2019). Chem. Mater..

[cit46] Carrillo-Carrión C., del Pino P., Pelaz B. (2019). Applied Materials Today.

[cit47] Momma K., Izumi F. (2011). J. Appl. Crystallogr..

[cit48] Wright A. D., Verdi C., Milot R. L., Eperon G. E., Pérez-Osorio M. A., Snaith H. J., Giustino F., Johnston M. B., Herz L. M. (2016). Nat. Commun..

[cit49] Guo Y., Yaffe O., Hull T. D., Owen J. S., Reichman D. R., Brus L. E. (2019). Nat. Commun..

[cit50] Nikl M., Mihokova E., Nitsch K., Somma F., Giampaolo C., Pazzi G. P., Fabeni P., Zazubovich S. (1999). Chem. Phys. Lett..

[cit51] Babin V., Fabeni P., Mihokova E., Nikl M., Pazzi G. P., Zazubovich N., Zazubovich S. (2000). Phys. Status Solidi B.

[cit52] Radhakrishna S., Pande K. P. (1973). Phys. Rev. B.

[cit53] Jacobs P. W. M. (1991). J. Phys. Chem. Solids.

[cit54] Yin J., Zhang Y., Bruno A., Soci C., Bakr O. M., Brédas J.-L., Mohammed O. F. (2017). ACS Energy Lett..

[cit55] Hoy J., Morrison P. J., Steinberg L. K., Buhro W. E., Loomis R. A. (2013). J. Phys. Chem. Lett..

[cit56] Imran M., Caligiuri V., Wang M., Goldoni L., Prato M., Krahne R., De Trizio L., Manna L. (2018). J. Am. Chem. Soc..

[cit57] Imran M., Ijaz P., Baranov D., Goldoni L., Petralanda U., Akkerman Q., Abdelhady A. L., Prato M., Bianchini P., Infante I., Manna L. (2018). Nano Lett..

[cit58] Imran M., Ijaz P., Goldoni L., Maggioni D., Petralanda U., Prato M., Almeida G., Infante I., Manna L. (2019). ACS Energy Lett..

[cit59] Wang Y., He J., Chen H., Chen J., Zhu R., Ma P., Towers A., Lin Y., Gesquiere A. J., Wu S.-T., Dong Y. (2016). Adv. Mater..

[cit60] Yoon S. J., Guo Z., dos Santos Claro P. C., Shevchenko E. V., Huang L. (2016). ACS Nano.

[cit61] Dujardin C., Auffray E., Bourret-Courchesne E., Dorenbos P., Lecoq P., Nikl M., Vasil’ev A. N., Yoshikawa A., Zhu R. (2018). IEEE Trans. Nucl. Sci..

[cit62] Tomanová K., Čuba V., Brik M. G., Mihóková E., Turtos R. M., Lecoq P., Auffray E., Nikl M. (2019). APL Mater..

