# Correction: Transforming colloidal Cs_4_PbBr_6_ nanocrystals with poly(maleic anhydride-*alt*-1-octadecene) into stable CsPbBr_3_ perovskite emitters through intermediate heterostructures

**DOI:** 10.1039/d0sc90125c

**Published:** 2020-06-18

**Authors:** Dmitry Baranov, Gianvito Caputo, Luca Goldoni, Zhiya Dang, Riccardo Scarfiello, Luca De Trizio, Alberto Portone, Filippo Fabbri, Andrea Camposeo, Dario Pisignano, Liberato Manna

**Affiliations:** Nanochemistry Department, Istituto Italiano di Tecnologia Via Morego 30 16163 Genova Italy dmitry.baranov@iit.it liberato.manna@iit.it; Analytical Chemistry Lab, Istituto Italiano di Tecnologia Via Morego 30 16163 Genova Italy; CNR NANOTEC, Institute of Nanotechnology c/o Campus Ecotecne, via Monteroni 73100 Lecce Italy; NEST, Istituto Nanoscience-CNR Piazza S. Silvestro 12 I-56127 Pisa Italy; Dipartimento di Fisica “Enrico Fermi”, Università di Pisa Largo Bruno Pontecorvo 3 I-56127 Pisa Italy

## Abstract

Correction for ‘Transforming colloidal Cs_4_PbBr_6_ nanocrystals with poly(maleic anhydride-*alt*-1-octadecene) into stable CsPbBr_3_ perovskite emitters through intermediate heterostructures’ by Dmitry Baranov *et al.*, *Chem. Sci.*, 2020, **11**, 3986–3995, DOI: 10.1039/D0SC00738B.

After the publication of our manuscript, an inquiry from a reader pointed out an earlier publication that was not cited in the context of prior art relevant to our study. We thank the reader for their interest in our work. Prompted by the inquiry, we thought it would be appropriate to acknowledge a few earlier and relevant publications that escaped our attention.

De Matteis *et al.*^1^ have reported room temperature excitation–emission maps (photoluminescence maps) of powders containing a mixture of Cs_4_PbBr_6_ and CsPbBr_3_ compounds (Fig. 9 and 10 in ref. 1). The photoluminescence maps show a dip at around ∼314 nm in the excitation spectrum of the CsPbBr_3_ compound emitting at ∼520 nm. The dip matches the wavelength of the electronic absorption in Cs_4_PbBr_6_. In a similar vein, Shin *et al.*^2^ have reported room temperature photoluminescence maps of CsBr/PbBr_2_ co-evaporated thin films containing a mixture of CsPbBr_3_ and Cs_4_PbBr_6_ compounds. In a photoluminescence map shown in Fig. 5b of ref. 2, the emission of the CsPbBr_3_ compound at ∼517 nm is quenched at the excitation wavelength of ∼318 nm, consistent with absorption by Cs_4_PbBr_6_. Both De Matteis *et al.*^1^ and Shin *et al.*^2^ observed an additional room temperature UV emission at ∼375 nm and ∼360 nm, respectively, from the mixed CsPbBr_3_–Cs_4_PbBr_6_ samples and assigned it to Cs_4_PbBr_6_.

The room temperature photoluminescence maps of Cs_4_PbBr_6_–CsPbBr_3_ heterostructured nanocrystals studied in our work (Fig. 4a)^3^ show a qualitatively similar dip in the intensity of ∼504 nm emission from CsPbBr_3_ when excited at ∼314 nm, the absorption wavelength of Cs_4_PbBr_6_. In contrast to the above-mentioned observations, Cs_4_PbBr_6_–CsPbBr_3_ heterostructured nanocrystals were not emissive in UV at room temperature but showed a weak ∼376 nm emission from Cs_4_PbBr_6_ only when cooled down to ∼35 K (Fig. 4b).^3^ The three studies share similar photoluminescence measurements and chemical formulas of the studied compounds. However, the synthetic origins and structures of the samples, together with discussions of the observed phenomena, are different in the three studies.

Krieg *et al.*^4^ have reported effective colloidal stabilization of CsPbBr_3_ nanocrystals over a wide range of concentrations, from 400 mg ml^−1^ to 4 × 10^−6^ mg ml^−1^ of inorganic content in toluene (Fig. 2 in ref. 4) by means of lecithin, a naturally occurring zwitterionic ligand. The lecithin-stabilized nanocrystals have been reported to be stable against multiple rounds of washing, *i.e.*, precipitation–redispersion with an antisolvent. The poly(maleic anhydride-*alt*-1-octadecene) compound (PMAO) used in our work to transform Cs_4_PbBr_6_ nanocrystals into CsPbBr_3_ nanocrystals yielded colloids of PMAO-capped CsPbBr_3_ nanocrystals which survive several rounds of washing and are stable in the concentration range of ∼26 mg ml^−1^ to ∼1 × 10^−4^ mg ml^−1^ (Fig. S32). It is notable that both lecithin and PMAO increase the colloidal stability of CsPbBr_3_ nanocrystals despite an apparently different surface binding chemistry and a different way of being introduced into the nanocrystal preparation.

The Royal Society of Chemistry apologises for these errors and any consequent inconvenience to authors and readers.
