# Supplementary material for: Transforming colloidal Cs4PbBr6 nanocrystals with poly(maleic anhydride-alt-1-octadecene) into stable CsPbBr3 perovskite emitters through intermediate heterostructures
Source: Chem Sci. 2020 Mar 20;11(15):3986–95. doi: 10.1039/d0sc00738b (PMC7116022; doi:10.1039/d0sc00738b)
Supplement: Supplementary file 1 [file SC-011-D0SC00738B-s001.pdf]

Electronic Supplementary Material (ESI) for Chemical Science.
This journal is © The Royal Society of Chemistry 2020

Supporting Information for

Transforming Colloidal Cs_4PbBr_6 Nanocrystals by Poly(maleic anhydride-alt-1-octadecene) into Stable $CsPbBr_3$ Perovskite Emitters Through Intermediate Heterostructures

Dmitry Baranov,[1]* Gianvito Caputo,[1] Luca Goldoni,[2] Zhiya Dang,[1] Riccardo Scarfiello,[3] Luca De Trizio,[1] Alberto Portone,[4] Filippo Fabbri,[4] Andrea Camposeo,[4] Dario Pisignano,[4,5] Liberato Manna [1]*

[1]*Nanochemistry Department, Istituto Italiano di Tecnologia, Via Morego 30, 16163 Genova, Italy*

[2]*Analytical Chemistry Lab, Istituto Italiano di Tecnologia, Via Morego 30, 16163 Genova, Italy*

[3]*CNR NANOTEC, Institute of Nanotechnology, c/o Campus Ecotecne, via Monteroni, 73100 Lecce, Italy*

[4]*NEST, Istituto Nanoscience-CNR, Piazza S. Silvestro 12, I-56127 Pisa, Italy*

[5]*Dipartimento di Fisica "Enrico Fermi", Università di Pisa, Largo Bruno Pontecorvo 3, I-56127 Pisa, Italy*

*Corresponding authors: dmitry.baranov@iit.it, liberato.manna@iit.it

Contents

S1. Experimental Details 3

a) Synthesis and isolation of Cs_4PbBr_6 nanocrystals (NCs) 3

b) Reaction of Cs_4PbBr_6 NCs with poly(maleic anhydride-alt-1-octadecene) (PMAO) in solution 4

c) Steady-state optical spectroscopy on liquid samples 4

d) Energy-dispersive X-ray spectroscopy (EDS) in STEM 5

e) Fourier transform infrared spectroscopy (FTIR) 5

f) Nuclear magnetic resonance (NMR) 6

g) Transmission electron microscopy (TEM) 6

h) X-ray diffraction (XRD) 7

i) PL quantum yield (QY) of NC samples in solution 7

j) High-resolution TEM (HRTEM) 7

k) NC-PMAO transformation in the film 8

S2. EDS-STEM 9

S3. ^{1}H and ^{1}H-^{13}C NMR investigation of surface passivation of Cs_4PbBr_6 NCs 13

S4. Evolution of the optical spectra at the onset of the transformation 18

S5. XRD patterns of Cs_4PbBr_6-$CsPbBr_3$ NC samples 19

S6. PLQY of NC samples in solution 20

S7. Reactivity of bulk Cs_4PbBr_6 powder with PMAO 22

S8. FTIR and NIR absorbance spectra 24

S9. ^{1}H NMR investigation of Cs_4PbBr_6-PMAO reactivity 26

S10. Control reaction between amine-free Cs_4PbBr_6 NCs and PMAO 34

S11. Stability of resulting $CsPbBr_3$/PMAO NCs 36

S12. Additional HRTEM images 41

S13. TEM size analysis before and after transformation 42

a) ~10 nm Cs_4PbBr_6 NCs to ~8 nm $CsPbBr_3$ NCs 42

b) ~16 nm Cs_4PbBr_6 NCs to ~12 nm $CsPbBr_3$ NCs 44

c) Calculations of conversion stoichiometry 46

S14. PL maps of NC samples at room and cryogenic temperatures 47

S15. Raman, μ-PL, and TRPL of $CsPbBr_3$/PMAO film 50

S16. References 53

S1. Experimental Details

a) Synthesis and isolation of Cs_4PbBr_6 nanocrystals (NCs)

Chemicals. Lead (II) bromide ($PbBr_2$, ≥98%, 211141-100G), cesium carbonate (Cs_2CO_3, 99%, 441902-100G), 1-octadecene (technical grade, 90%), oleylamine (≥98% primary amine, HT-OA100-1.5KG or 70% technical grade), oleic acid (technical grade, 90%), toluene, hexane, and tetrachloroethylene were purchased from Sigma Aldrich and used as received without further purification. Lead (II) bromide was opened, stored, and handled inside a nitrogen-filled glovebox. Volumes of liquid reagents were measured using either disposable syringes or mechanical micropipettes.

Procedure. The synthesis of Cs_4PbBr_6 NCs was adapted from the work of Akkerman et al.[1] with two modifications: 1) reaction was performed in air, 2) 72 mg of $PbBr_2$ were used instead of 36 mg. The stock solution of the cesium oleate precursor was prepared by dissolving 400 mg of Cs_2CO_3 in 8 ml of oleic acid at 100 ^oC under stirring on air. Upon cooling to room temperature, the cesium oleate precursor remained clear and transparent.

For the NC synthesis, room temperature cesium oleate was injected into the reaction mixture containing $PbBr_2$ dissolved in a mixture of ligands. For a single batch of NCs, 72±2 mg of $PbBr_2$ were combined with 5 ml of octadecene-1, 1.5 ml of oleylamine, and 0.2 ml of oleic acid in a 20 ml glass vial. The mixture was heated to ~120-130 ^oC under stirring on top of the hotplate until all visible solid $PbBr_2$ got dissolved, forming a clear colorless solution. The $PbBr_2$ dissolution typically took less than 5 minutes. Next, the vial was transferred into a machined aluminum block pre-heated to 80-82 ^oC, and the temperature of the reaction mixture was let to cool down and stabilize at ~80^oC. At that point, 0.5 ml of the cesium oleate precursor were swiftly injected. The injection of cesium oleate generally resulted in the formation of a cloudy mixture, which was removed from the heating and allowed to cool in the air under stirring. Two different outcomes of the cesium oleate injection have been observed over the course of the work (about ~40 separate syntheses). 1) The reaction mixture gradually turns cloudy within the first 1-2 minutes after the injection of cesium oleate. In that case, the reaction was cooled down after a total of 2 minutes of elapsed time after the cesium oleate injection. 2) The reaction mixture remains clear and colorless after the injection of cesium oleate precursor. In that case, the reaction mixture was kept at ~80 ^oC for 10 minutes, after which it was allowed to cool to room temperature turning cloudy white over the course of the cooling. Regardless of the scenario, the isolated NCs synthesized by this procedure had a narrow size distribution and an average size in the 10-16 nm range.

NC Isolation. Once the reaction mixture cooled down to the room temperature, it was split equally into four 4 ml vials using a Pasteur pipette. The vials were centrifuged for 3 minutes at 4000 rpm, yielding white to light grey precipitate at the bottom and a clear supernatant. The supernatant was discarded. To further clean the precipitate from residual supernatant, the vials were centrifuged again (1 min at 4000 rpm) to collect the residual non-decanted supernatant. For that centrifugation step, the vials were oriented in the centrifuge such that the precipitate was pointing outwards, so the remaining liquid could be collected in the lower and opposite (with respect to precipitate) part of the vial. After the centrifugation, the liquid was removed by a

cotton tip, and the centrifugation step repeated to collect all of the visible residual supernatant from the vial. Such centrifugation allows isolation of Cs_4PbBr_6 NCs without using antisolvents. NCs were dissolved in anhydrous toluene, hexane, tetrachloroethylene, or deuterated solvents for subsequent experiments. NCs were used within a week after the synthesis, as storing them longer in air results in the loss of solubility, possibly due to coalescence. Attempts to wash Cs_4PbBr_6 NCs by precipitation with antisolvents resulted in either immediate (acetone) or gradual (ethyl acetate) and irreversible aggregation of NCs.

b) Reaction of Cs_4PbBr_6 NCs with poly(maleic anhydride-alt-1-octadecene) (PMAO) in solution

Chemicals. Poly(maleic anhydride-alt-1-octadecene) (PMAO) reagent of two varieties was tested: Sigma-Aldrich, #419117, average M_n 30,000-50,000, light yellow-colored powder, and Polysciences, Inc., # 05152, Poly(maleic anhydride 1-octadecene) 1:1 (molar), M_w 30,000-50,000, very light yellow almost white powder, giving similar results.

Procedure. An optically clear stock solution of PMAO in toluene with a polymer concentration of ~85 mg/ml was prepared by dissolving 170 mg of PMAO in 2 ml of anhydrous toluene under shaking at room temperature (~21 ^oC). Mild heating with a heat gun could be applied to facilitate the PMAO dissolution. Once all PMAO dissolved (by visual inspection), the resulting solution was filtered through a hydrophobic 0.45 micron PTFE filter. For the transformation reactions, 1/4 of the NC batch (a full amount of NC solid from one of the four vials, as described above) was dissolved in 2 ml of toluene to form a Cs_4PbBr_6 NC stock solution.

Typically, the reactions were performed between small aliquots of the NC stock solution (50-100 μL) and PMAO (5-80 μL). After the mixing of the desired amount of NCs and PMAO, the clear and colorless reaction mixture begins to acquire bright green color within tens of seconds or a few minutes, depending on the NC batch. The change in color indicates the formation of $CsPbBr_3$. The PMAO amounts necessary for a complete or partial transformation have been empirically determined for a given batch of Cs_4PbBr_6 NCs by mixing a fixed volume of NC solution with various volumes of the PMAO stock solution, followed up by checking UV-Vis absorbance spectra (disappearance of the ~314 nm peak of Cs_4PbBr_6 NCs over time indicates the reaction progress). The described reaction conditions are typical, but exact amounts of reagents may vary from batch to batch, dryness of the solvents, and other parameters that were not controlled.

The full batch of Cs_4PbBr_6 NCs can be fully-converted to $CsPbBr_3$ by dissolving the solid of isolated Cs_4PbBr_6 NCs in 1-2 ml of toluene and mixing it with 6 ml of 85 mg/ml solution of PMAO in toluene and leaving the reaction mixture on a shaker overnight at room temperature.

c) Steady-state optical spectroscopy on liquid samples

Samples of NCs, PMAO, and their mixtures were prepared as dilute toluene solutions in 4 mm by 10 mm quartz cuvettes (Hellma Analytics, 114F) and were used for the measurements in UV-Vis spectral region. Samples of NCs and 1-octadecene, oleylamine, and cesium oleate stock solution were prepared as dilute solutions in tetrachloroethylene in 10 mm by 10 mm

quartz cuvettes (Firefly Sci, 41FL-UV-10) for absorbance measurements in near-infrared (NIR) spectral region.

Optical absorbance spectra in UV-Vis spectral region were collected using Cary 500 spectrophotometer, and Cary 5000 spectrophotometer was used to collect the spectra in NIR spectral region. In each case, the spectra were corrected for the absorbance of the solvent blank by subtracting the absorbance spectra of (cuvette + solvent) from the sample spectra. Photoluminescence (PL) spectra in 400-600 nm wavelength region were collected using Cary Eclipse spectrofluorimeter with excitation wavelength set at 350 nm, excitation and emission slits were typically set at 2.5 nm each.

d) Energy-dispersive X-ray spectroscopy (EDS) in STEM

Materials. Cesium bromide (CsBr, 99.9% trace metal basis, Aldrich, 202142-100G, Lot# 0000020256), lead bromide ($PbBr_2$, ≥98%, 211141-100G, Lot# BCBW1334), two replica batches of Cs_4PbBr_6 NCs synthesized as described above.

The phase purity of CsBr and $PbBr_2$ commercial powders was confirmed by XRD. For XRD, the samples of bulk CsBr and $PbBr_2$ powders were prepared by mixing finely ground powders with a small amount of high vacuum silicone grease (Dow Corning®) and compacting it on top of a zero-diffraction silicon wafer.

EDS in Scanning TEM (EDS-STEM). EDS spectra in STEM were collected using the JEOL-JEM1400 electron microscope operating at 120 kV acceleration voltage. Samples of bulk CsBr and $PbBr_2$ powders were prepared by drop-casting toluene suspensions of the finely ground powders onto amorphous carbon-covered Cu grids. Grids prepared in that way contained sub-micron particles suitable for EDS-STEM analysis of "bulk" materials. The Cs_4PbBr_6 NC samples were prepared by drop-casting dilute solutions onto amorphous carbon-covered Cu grids. The EDS data were acquired in STEM mode, sample tilt 6°, spot size was adjusted between S2 or S3 to optimize the signal counts. STEM images and corresponding EDS spectra were collected from several areas of the grid.

Quantification of elemental composition in EDS-STEM. Collected EDS spectra were analyzed using Analysis Station software ver. 3.8.0.59 (JEOL Engineering Co., Ltd), JED Series AnalysisProgram ver. 3.8.0.37. Cs, Br, and Pb were chosen as the only elements for quantification (other detected elements were C, O, Cu, and Cr – they originate from the TEM grid). Cs L, Br K, and Pb L lines were used for the quantification. Quantification of the EDS spectra was performed using a standardless Ratio quantification method built into the software.

e) Fourier transform infrared spectroscopy (FTIR)

A solid of as-synthesized NCs was dispersed in toluene, and a drop of the toluene solution was mixed with 100 mg of KBr powder. After that, the mixture was dried under vacuum at 40°C and compressed into a pellet for FTIR measurements. A similar procedure was employed to record the FTIR spectrum of the cesium oleate precursor. The measurements were carried out using a Bruker Vertex 70 spectrometer in transmission mode from 4000 to 500 cm^{-1} with 64 scans.

f) Nuclear magnetic resonance (NMR)

The NC only, NC-PMAO, PMAO, and model mixture solutions of ligands were prepared in toluene-d_8 and transferred into 5 mm diameter tubes for the NMR experiments. The destructive ligand composition analysis was performed by dissolving the solid of NCs (as isolated after the synthesis) in 500-600 μL of DMSO-d_6 with the addition of 5-7 μL of trifluoroacetic acid (TFA) and performing the NMR experiment in a 3 mm or a 5 mm diameter tube.

^{1}H NMR spectra (**Figures S7, S8, S9, S20, S21, S22, S23a-c, S24-S26**) were acquired on a Bruker Avance III 400 MHz spectrometer equipped with a Broad Band Inverse probe (BBI) at 300.0 K. 32-128 transients (depending on the sample concentration), and 64k of digit points were accumulated after applying a 90-pulse, with a relaxation delay of 30 s, over a spectral width of 20.55 ppm and with the offset centered at 6.18 ppm.

^{1}H-^{13}C HSQC (multiplicity edited Heteronuclear Single Quantum Coherence, hsqcedetgp, Bruker library, Bruker Avance III 400 MHz spectrometer) spectrum of oleylamine/succinic anhydride mixture (**Figure S23d,e**) was acquired with 16 transients, 1020 data points, and 256 increments, over a spectral width of 15.43 and 165.0 ppm (offset at 6.49 and 75.0 ppm) for ^{1}H and ^{13}C, respectively.

^{1}H-^{13}C HMBC (Heteronuclear Multiple-Bond Correlation, hmbcgplpndqf, Bruker library, Bruker Avance III 400 MHz spectrometer) spectrum of oleylamine/succinic anhydride mixture (**Figure S23f**) was acquired by using 16 transients, 512 data points, and 128 increments, over a spectral width of 12.99 ppm and 222.1 ppm (offset at 5.00 and 99.8 ppm) for ^{1}H and ^{13}C respectively.

^{1}H-^{13}C HSQC (multiplicity edited Heteronuclear Single Quantum Coherence, hsqcedetgpsp.3, Bruker library) experiments for NC characterization (**Figure S6**) were conducted on a Bruker Avance III 600 MHz spectrometer, equipped with 5 mm QCI cryoprobe, using 24 scans, 2048 data points, and 648 increments, over a spectral width of 15.00 ppm for ^{1}H and 200.0 ppm for ^{13}C, with the offsets at 6.00 and 110.0 ppm respectively.

g) Transmission electron microscopy (TEM)

The as-synthesized Cs_4PbBr_6 NCs and $CsPbBr_3$ NCs derived from them after PMAO-induced transformation were imaged using JEM 1400-Plus JEOL electron microscope operating at an acceleration voltage of 120 kV. The samples for TEM experiments were prepared by drop-casting sample solutions onto carbon-coated copper grids with a pipette and letting the solvent evaporate.

The images were collected using DigitalMicrograph software (version 3.11.1048.0) and processed using ImageJ software (version 1.51j8)[2] and a different version of DigitalMicrograph (1.71.38) equipped with PASAD plugin for Fast Fourier Transform (FFT) image analysis (PASAD-tools, v1.0).[3] The thresholding analysis was performed on the images of Cs_4PbBr_6 NCs for their size determination (**Section S13**),[4, 5] while FFT analysis was performed on the images of $CsPbBr_3$ NCs for their size determination (**Section S13**).[6]

h) X-ray diffraction (XRD)

XRD patterns were collected using a PANalytical Empyrean X-ray diffractometer equipped with a Cu Kα cathode (λ=1.5406 Å) operating at 45 kV and 40 mA. The NC and PMAO-NC samples were prepared for XRD by drop-casting from toluene solutions onto a zero-diffraction silicon wafer and letting the solvent evaporate. The XRD data processing and analysis were performed in HighScore ver. 4.7 software using "*Search Peaks…*" and "*Search Match…*" options.

i) PL quantum yield (QY) of NC samples in solution

The PLQYs of partially- and fully-transformed Cs_4PbBr_6-$CsPbBr_3$ NC samples were measured on diluted samples using FLS920 Edinburgh Instruments spectrofluorimeter equipped with an integrating sphere. The samples for PLQY were prepared in the atmosphere of air or a nitrogen-filled glovebox by diluting concentrated PMAO-NC solutions with 1 ml of toluene (anhydrous toluene was used for the NC sample prepared in the glovebox) in 4 mm x 10 mm quartz cuvettes capped with white PTFE stoppers (Hellma-Analytics, part number 114F-10-40). Dilutions were performed shortly before the measurements.

The samples were excited at 400 nm using the xenon lamp (Xe900) with an excitation slit width set at 10 nm. The emission slit width was set at 0.20-0.25 nm. The cuvettes were oriented inside the sphere such that the excitation was through the 4 mm path length. The photon number spectra for the samples and toluene solvent reference were collected over 375-625 nm spectral range (375-600 nm range is plotted in **Figures S13-S15**) with a step size of 1 nm, and a dwell time of 0.2 seconds per step. Five consecutive scans of each measurement (excitation and scatter spectra) were added together to obtain the data for PLQY calculations. Corrections for the background, PMT detector sensitivity, and the lamp reference detector were applied automatically during the data collection by the software. For PLQY calculations, the photon number spectra were integrated in the range of 385-415 nm in order to calculate the total number of scattered photons (sc photons), and in the ranges 465-575 nm (partially- and fully-transformed NCs in the air) or 435-575 nm (fully-transformed NCs in the glovebox) to determine the total number of emitted photons (em photons). The values of PLQY were calculated using the following formula: $PLQY,\% = 100 * (N_{em\ photons}^{sample} - N_{em\ photons}^{reference})/(N_{sc\ photons}^{reference} - N_{sc\ photons}^{sample})$, and reported without correction for self-absorption.

j) High-resolution TEM (HRTEM)

The partially-converted Cs_4PbBr_6-$CsPbBr_3$ NC heterostructures were prepared by mixing Cs_4PbBr_6 NCs with an amount of PMAO pre-determined to produce partially converted samples. Within minutes after mixing, already green-looking NCs-PMAO solution was drop-casted onto an ultrathin carbon/holey carbon-coated 400 mesh copper grids and placed into a JEOL JEM-2200FS microscope for HRTEM investigation. The microscope operates at 200 kV, and it is equipped with a CEOS spherical aberration corrector for the objective lens and an in-column image filter (Ω-type).

k) NC-PMAO transformation in the film

Film preparation. An 85 mg/mL solution was obtained by dissolving 170 mg of PMAO in 2 mL of toluene. After mixing by vortex until the complete dissolution of the polymer powder, the so-obtained solution was filtered through a 0.45 μm PTFE syringe filter. 80 μL of PMAO solution were mixed with 50 μL of Cs_4PbBr_6 NCs dispersed in toluene, in a glass vial. After a brief shake for a few seconds, 20 μL drop was cast on top of a quartz substrate (1×1 cm^2). The samples were dried under a gentle nitrogen flow in order to obtain solid-state films for further spectroscopic analysis.

Photoluminescence. Photoluminescence (PL) spectra were measured by exciting the samples with a continuous wave (CW) diode laser (emission wavelength of 405 nm, spot size about 2 mm and excitation power 0.7 mW), and collecting the spectra with a fiber-coupled spectrometer (Flame, Ocean Optics). Samples were mounted in an integrating sphere, which allows for measuring concomitantly the film absorbance and the photoluminescence quantum yield (QY).[7] In order to investigate the reaction evolution, these measurements were performed at regular time intervals over a period of 2 hours starting a few minutes after the mixing of the Cs_4PbBr_6 NCs and PMAO solution, and the film preparation.

Fluorescence confocal microscopy was carried out by using an inverted microscope (Olympus), equipped with a confocal laser scanning head. The samples were excited through a 10× objective (Numerical aperture: 0.4) by a 405 nm CW laser, and collecting the emitted photons through the same objective in a back-scattering configuration.

Time-resolved photoluminescence spectra were measured by utilizing a streak camera (Hamamatsu), coupled to a spectrometer (Princeton). The sample was optically pumped by a pulsed laser (Legend, Coherent) with an emission wavelength of 400 nm and a pulse duration of about 100 femtoseconds.

Raman. Micro-Raman measurements were performed with a Renishaw InVia spectrometer equipped with a confocal optical microscope. Samples were excited by a 785 nm laser, using a 100× objective and 1 μm excitation spot size. The 785 nm excitation laser allows avoiding PL signals from the studied samples. Typical acquisition times were of the order of 10 s, which allowed for collecting Raman spectra at time intervals of 1-5 minutes and for investigating the evolution of the reaction in the films.

S2. EDS-STEM

The EDS-STEM analysis of NC monolayer yielded ~$Cs_{4.9}Pb_1Br_{5.5}$ stoichiometry, which is Cs-rich and can be explained by the presence of cesium oleate on the NC surface. Atomic compositions below are reported as an average of the results from three different areas ± standard deviation unless otherwise noted.

EDS-STEM,

CsBr sub-micron particles. **Cs**, 56%±3%, **Br**, 44%±3%.

$PbBr_2$ sub-micron particles (average of five). **Pb**, 29.2%±1.6%, **Br**, 70.8%±1.5%.

Cs_4PbBr_6, NC batch 1, **Cs**, 43.3%±1.2%, **Pb**, 8.9%±0.3%, **Br**, 47.8%±1.4%.

Cs_4PbBr_6, NC batch 2, **Cs**, 43.1%±0.3%, **Pb**, 8.6%±0.7%, **Br**, 48.3%±0.8%

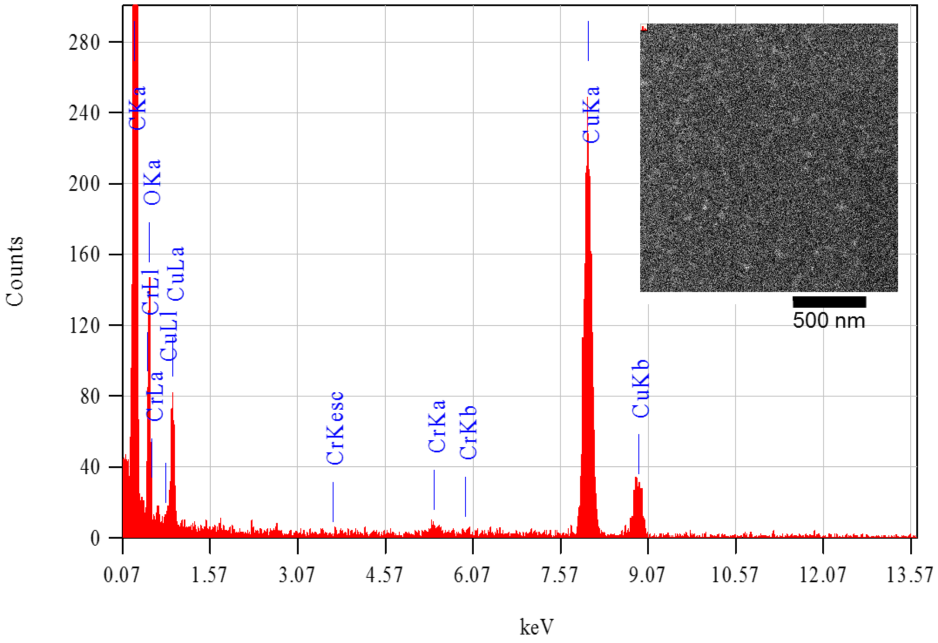

Figure S1. Representative EDS spectrum and STEM image (inset) of the analyzed area from an empty area on the carbon-coated Cu grid showing background containing carbon, oxygen, copper, and chromium.

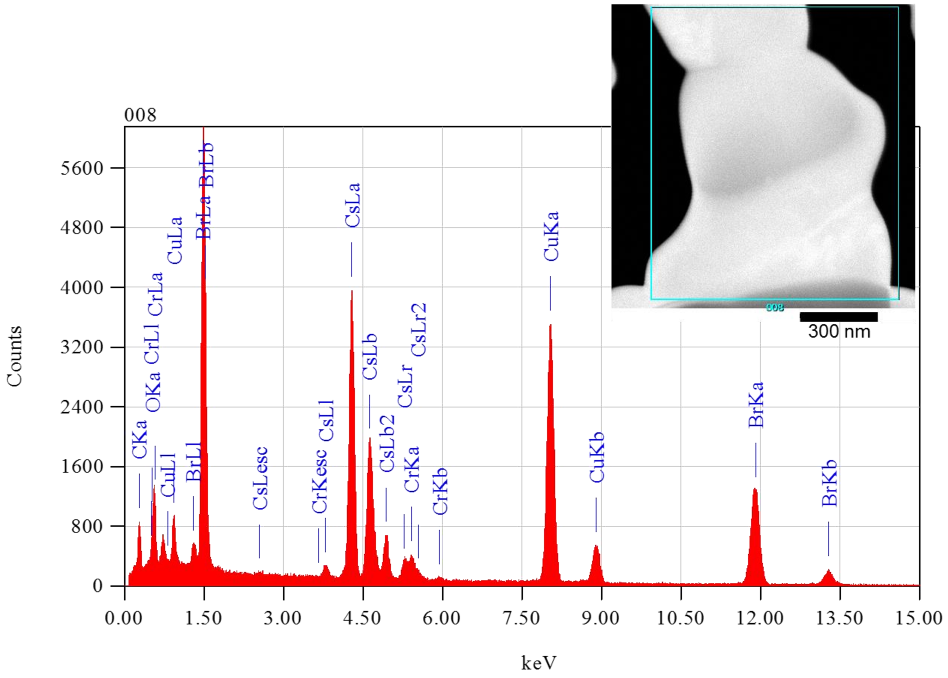

Figure S2. Representative EDS spectrum and STEM image (inset) of the analyzed area from a sub-micron particle of bulk CsBr. Cyan box indicated the area from which the EDS spectrum was collected. Cs, 54.2 at.%, Br, 45.8 at.%

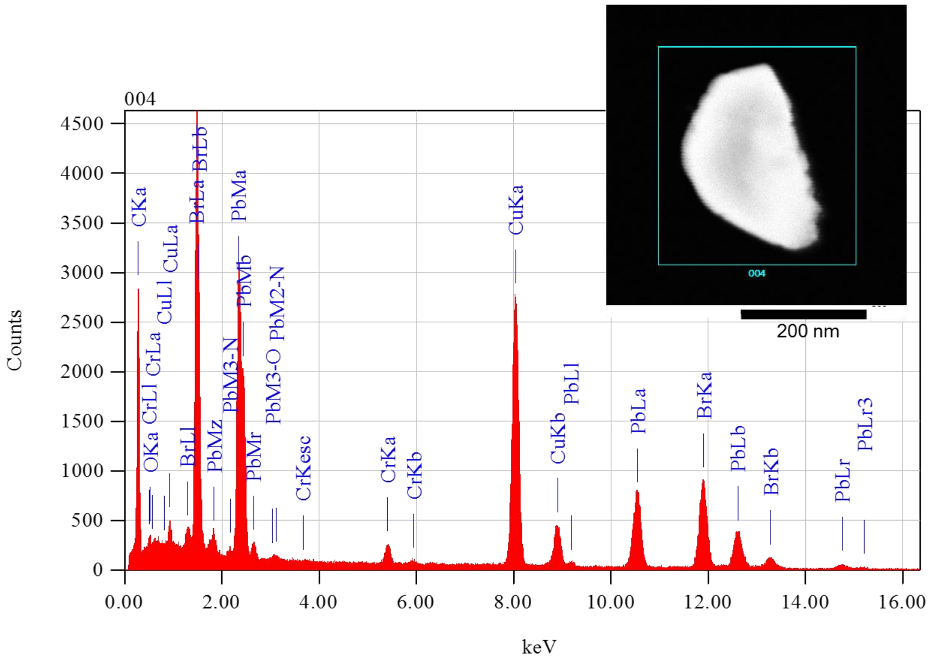

Figure S3. Representative EDS spectrum and STEM image (inset) of the analyzed area from a sub-micron particle of bulk $PbBr_2$. Cyan box indicated the area from which the EDS spectrum was collected. Pb, 30.3 at.%, Br, 69.7 at.%

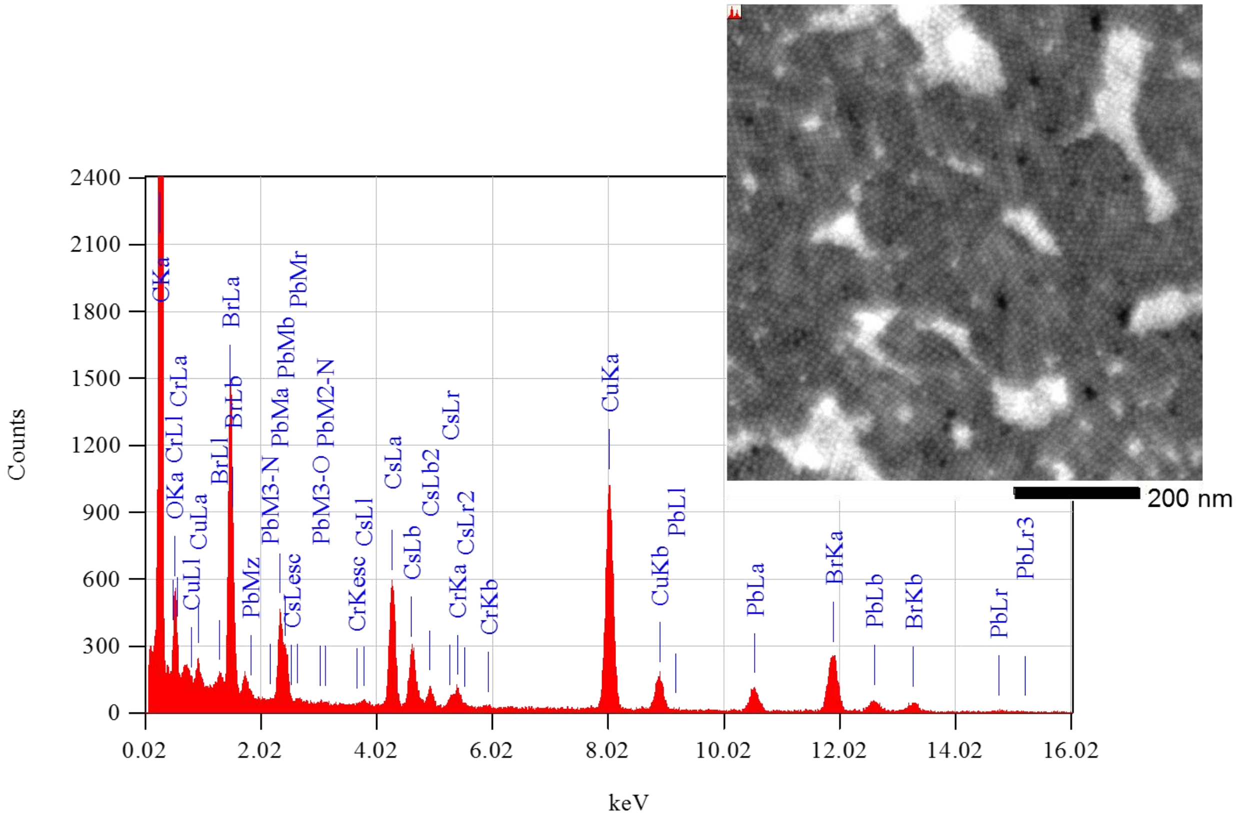

Figure S4. Representative EDS spectrum and STEM image (inset) of the analyzed area from a Cs_4PbBr_6 NC sample monolayer (batch 1). Cs, 42.6%, Pb, 8.9 at.%, Br, 48.5 at.%

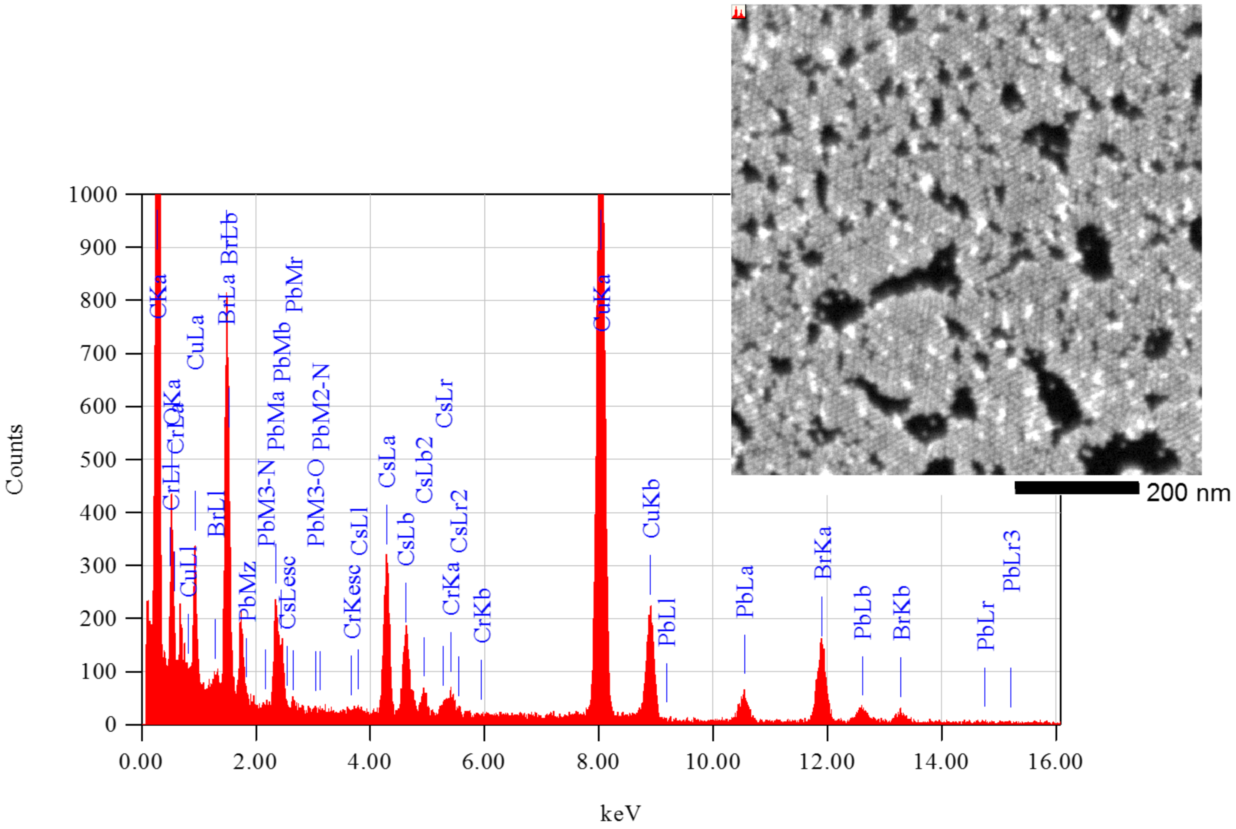

Figure S5. EDS spectrum and STEM image (inset) of the analyzed area from a Cs_4PbBr_6 NC sample monolayer (batch 2). Cs, 43.2%, Pb, 7.9 at.%, Br, 48.8 at.%

S3. ^{1}H and ^{1}H-^{13}C NMR investigation of surface passivation of Cs_4PbBr_6 NCs

An NMR characterization of the Cs_4PbBr_6 NC solutions was performed to identify and quantify organics in the samples. ^{1}H NMR spectra contained broadened resonances attributable to oleylamine/oleylammonium and oleate moieties, and sharp resonances belonging to the residual octadecene-1 and non-deuterated toluene (**Figures S6-S8**). The broadened peaks in the ^{1}H NMR spectra indicate that all of the organic species, with the exception of solvents, were either bound or in dynamic interaction with NC surface.[8-11] In order to quantify the relative amine/acid ratio in the NC samples, we employed a destructive method by dissolving NCs in DMSO-d_6 and adding a small amount of trifluoroacetic acid. Trifluoroacetic acid facilitates the NC dissolution and additionally acts as a protonating agent for organics, which helps to separate the α-CH_2 signals of oleylamine from that of oleic acid in the ^{1}H NMR spectrum, thus circumventing peak overlap. The ratio between the integrated peak intensities of α-CH_2 resonances yielded amine/acid ratios of ~3:2 (**Figure S9**). The presence of oleylamine and oleic acid on the NC surface was further confirmed by two-dimensional ^{1}H-^{13}C heteronuclear single quantum coherence (HSQC) measurements in toluene-d_8 in which α-CH_2 resonances from both ligands (**Figure S6**) were observed, with the chemical shifts (^{1}H 2.86 ppm, ^{13}C 41.40 ppm) for oleylamine, and (2.54, 38.81) for oleic acid. The 2.86 ppm shift of α-CH_2 of oleylamine is similar to that in an equimolar mixture of oleylamine and oleic acid from a prior study (Figure 4c in ref.[12]). That similarity points to the NCs containing a mixture of neutral and protonated oleylamine. The protonated oleylamine is most probably in the form of oleylammonium oleate, as is the case of $CsPbBr_3$ NCs synthesized in a similar reaction environment.[10] Overall, the structure of Cs_4PbBr_6 NCs is that of an inorganic crystalline core surrounded by a ligand shell consisting of cesium oleate (to account for the Cs-rich elemental composition), and oleylamine/oleylammonium oleate species.

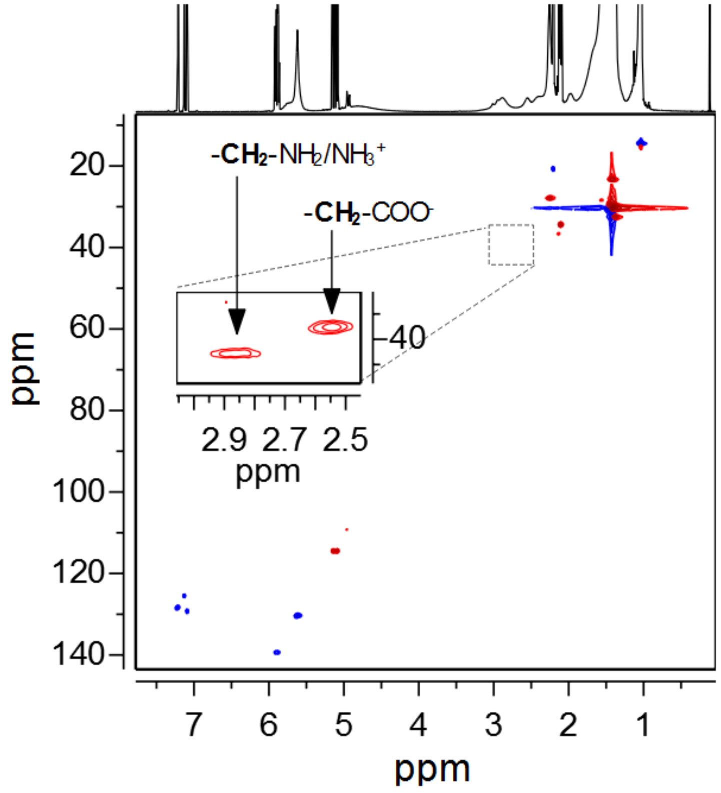

Figure S6. ^{1}H-^{13}C HSQC NMR spectrum of the Cs_4PbBr_6 NCs in toluene-d_8, with insets showing a close-up of the region with signals from α-CH_2 from oleate and partially-protonated oleylamine species.

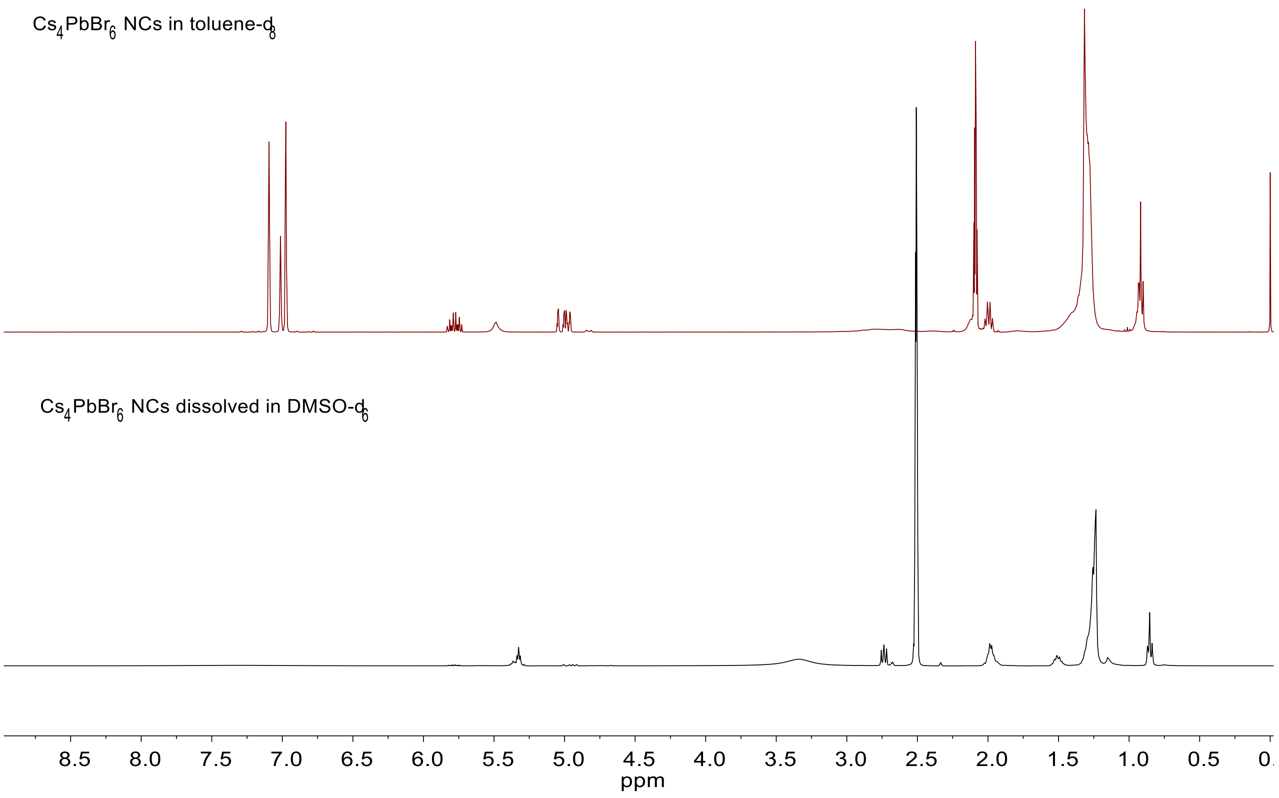

Figure S7. Full range ^{1}H NMR spectra of Cs_4PbBr_6 NCs dispersed in toluene-d_8 (top spectrum) or dissolved in DMSO-d_6 (bottom spectrum). Annotated spectra are shown in the next figure.

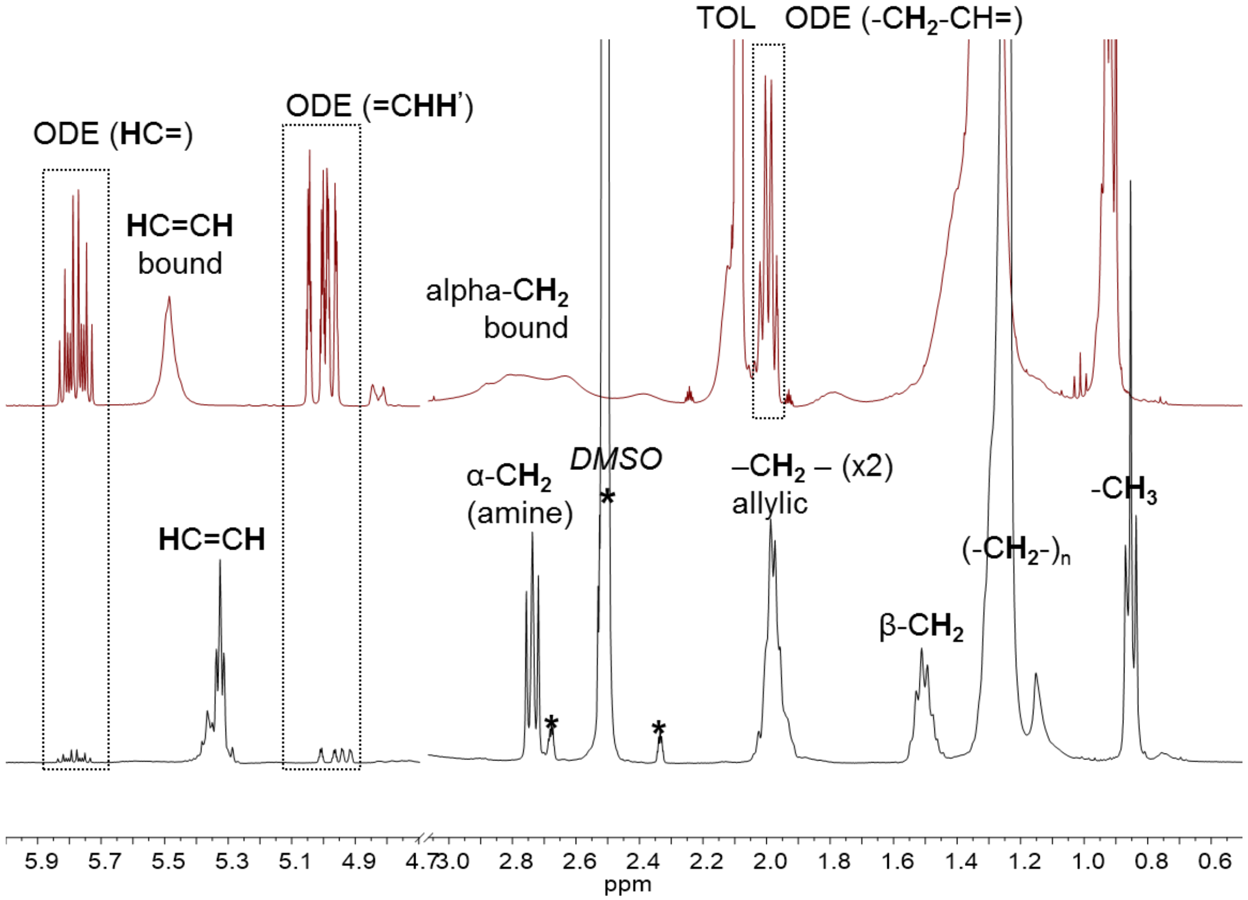

Figure S8. Annotated ^{1}H NMR spectra of Cs_4PbBr_6 NCs dispersed in toluene-d_8 (top spectrum) and dissolved in DMSO-d_6 (bottom spectrum, DMSO signal is marked with asterisks). The signal from α-CH_2 in oleate (at ~2 ppm) is overlapped with the signal from allylic hydrogens, "TOL" and "ODE" mark signals from residual toluene and 1-octadecene. The addition of trifluoroacetic acid (**Figure S9**) allows us to clearly separate α-CH_2 signals from two ligands.

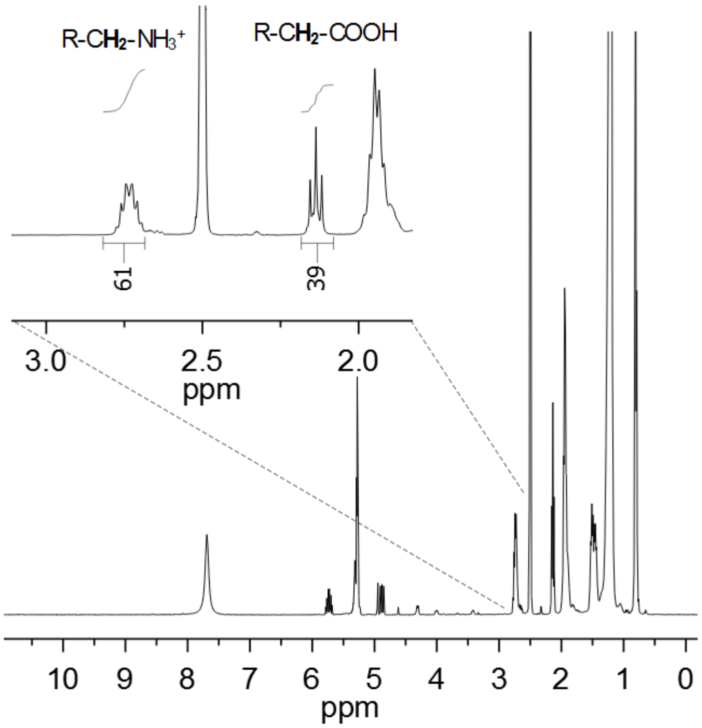

Figure S9. ^{1}H NMR spectrum of Cs_4PbBr_6 NCs dissolved in DMSO-d_6 after the addition of small amounts of protonating agent trifluoroacetic acid for quantification of the ratio between oleylamine (multiplet at 2.72 ppm from α-CH_2) and oleic acid (multiplet at 2.14 ppm from α-CH_2).

S4. Evolution of the optical spectra at the onset of the transformation

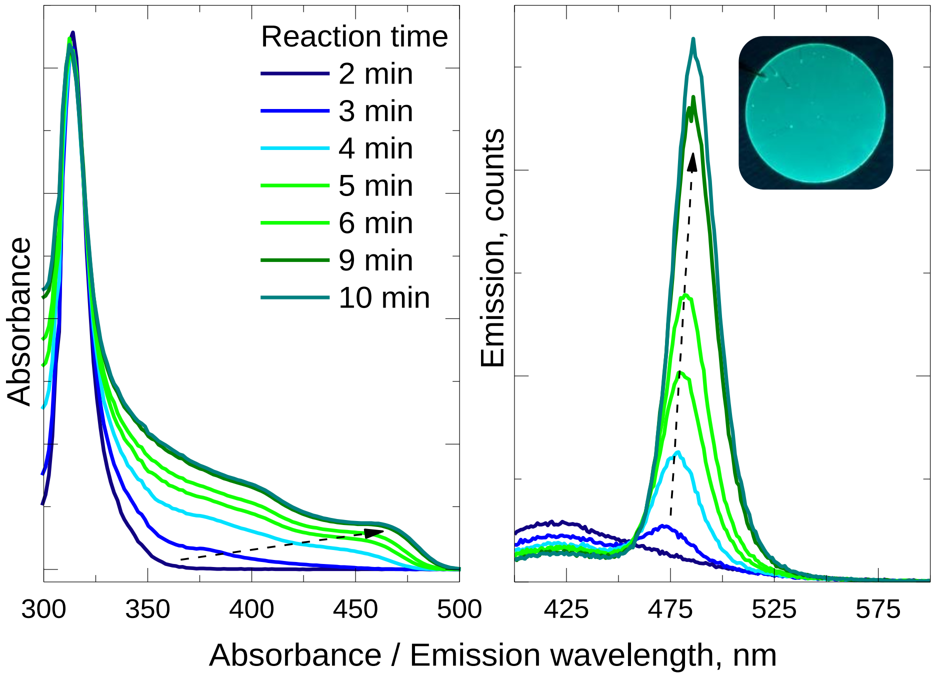

Figure S10. Evolution of steady-state optical absorbance (left panel) and PL (right panel) spectra during the first moments of the PMAO-induced transformation of Cs_4PbBr_6 NCs into $CsPbBr_3$ in toluene solution at room temperature. PL spectra recorded with 350 nm excitation. Inset shows a cyan-luminescent spin-cast film of partially-transformed NCs on top of a microscope glass cover slide (1" diameter) under 375 nm UV lamp excitation.

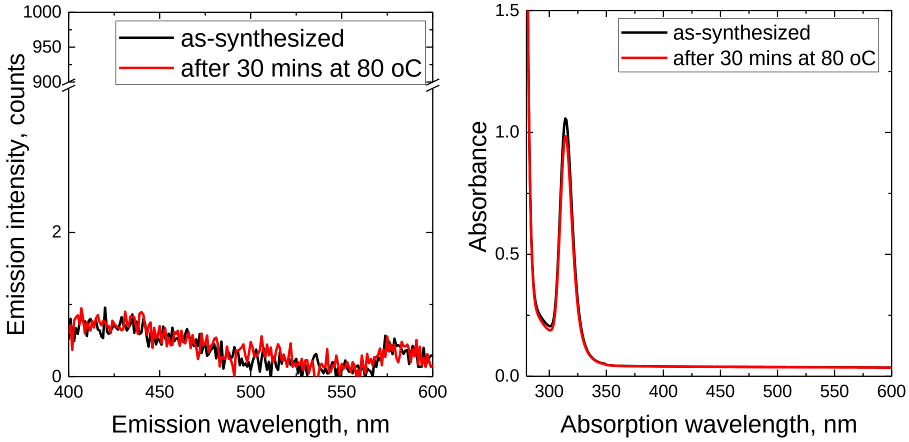

Figure S11. Photoluminescence (left) and absorbance (right) spectra of as-synthesized Cs_4PbBr_6 NCs in toluene before (black curves) and after (red curves) incubating the NC solution at 80 ^oC for 30 minutes. The absence of significant changes in both spectra confirms that there is no spontaneous conversion of Cs_4PbBr_6 NCs to $CsPbBr_3$ at 80 ^oC in the absence of PMAO.

S5. XRD patterns of Cs_4PbBr_6-$CsPbBr_3$ NC samples

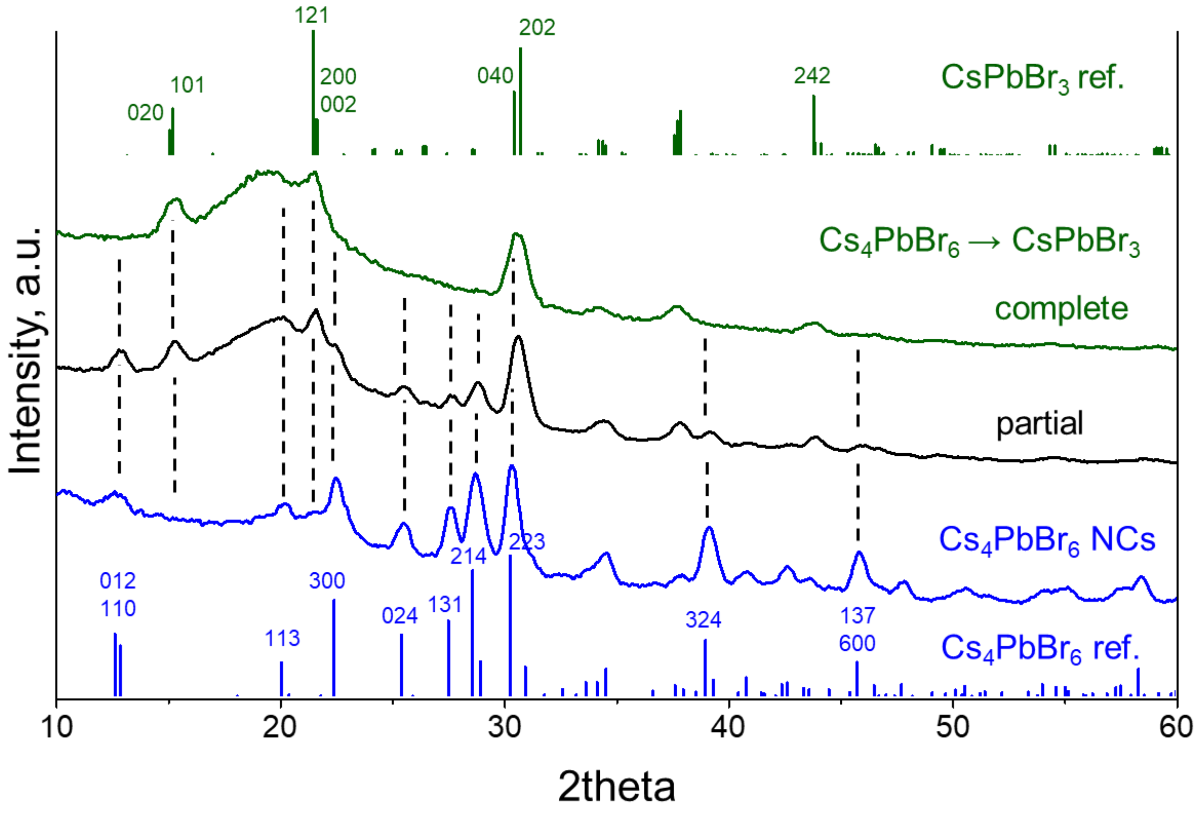

Figure S12. XRD patterns annotated with *hkl* indices for the samples of Cs_4PbBr_6 NCs: starting, partially- and fully-transformed in $CsPbBr_3$ via reaction with PMAO in toluene (reproduced from **Figure 2a**). The vertical dashed lines highlight the disappearance of selected Cs_4PbBr_6 reflections and the appearance of the ones corresponding to $CsPbBr_3$. Top and bottom stick patterns are those of the reference bulk compounds: rhombohedral Cs_4PbBr_6 (pattern ID 04-015-9683, ICSD code 162158)[13] and orthorhombic $CsPbBr_3$ (pattern ID 96-451-0746, COD code 4510745).[14]

S6. PLQY of NC samples in solution

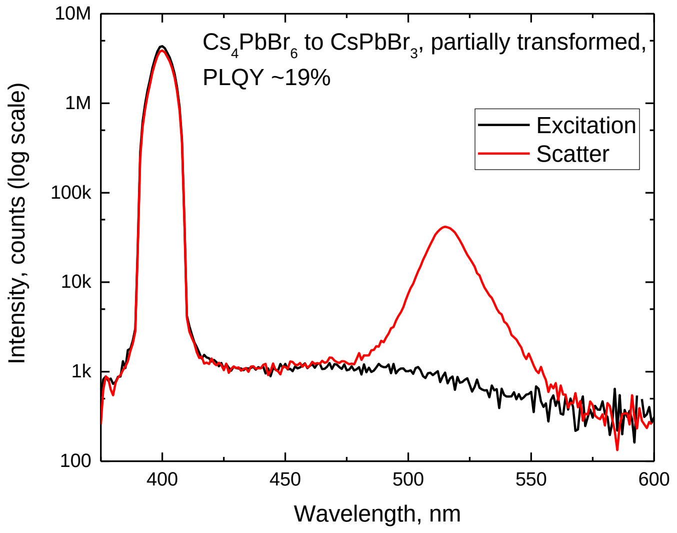

Figure S13. Absolute photon scattering spectra recorded with an integrating sphere from a partially-transformed Cs_4PbBr_6 to $CsPbBr_3$ NC sample (see **Figure 1c** for UV-Vis absorbance and PL spectra of the sample).

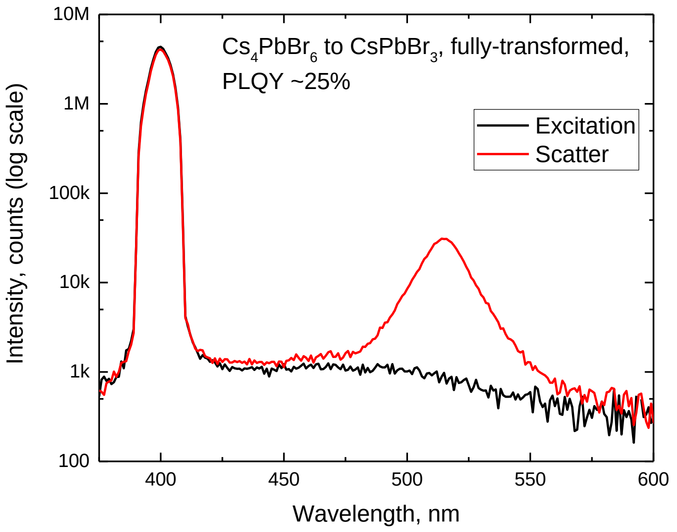

Figure S14. Absolute photon scattering spectra recorded with an integrating sphere from a fully-transformed Cs_4PbBr_6 to $CsPbBr_3$ NC sample (see **Figure 1c** for UV-Vis absorbance and PL spectra of the sample) with PMAO.

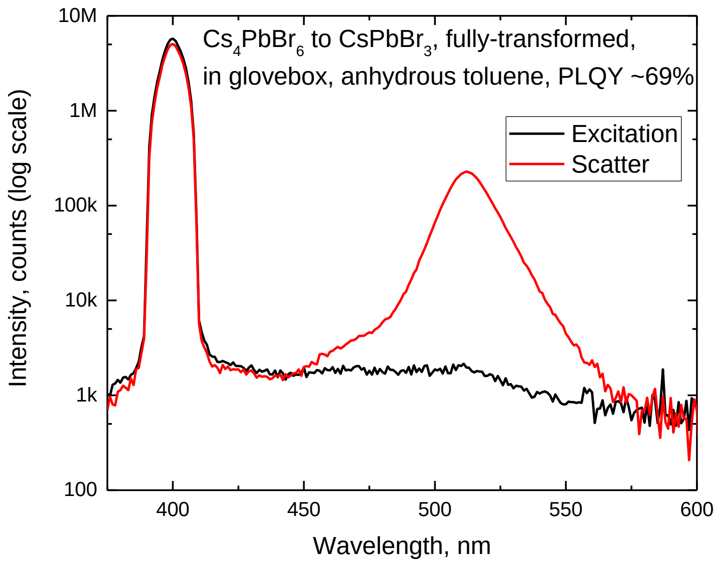

Figure S15. Absolute photon scattering spectra recorded with an integrating sphere from a fully-transformed Cs_4PbBr_6 to $CsPbBr_3$ NC sample with PMAO. The transformation was performed inside the nitrogen-filled glovebox, and anhydrous toluene was used as a solvent for NCs and PMAO.

S7. Reactivity of bulk Cs_4PbBr_6 powder with PMAO

A small portion (~7 mg) of green-emitting Cs_4PbBr_6 powder containing an impurity of CsBr (its preparation and characterization are described in work by Ray et al.[15]) was mixed with 1 ml of ~43mg/ml toluene solution of PMAO. The amounts were chosen to test the reactivity under conditions of a large excess of PMAO (molar ratio between Cs_4PbBr_6 and PMAO estimated ~1:22). The starting Cs_4PbBr_6 powder had the appearance of white powder with a light green tint. No change in its appearance was observed within the first hour after mixing with PMAO. Next, the sample was heated to 80 ^oC for 5 hours, briefly sonicated, and put on an automatic shaker overnight (~11 hrs). In the end, there was a color change from light green to light yellow (**Figure S16**). Trace amounts of $CsPbBr_3$ were detected by XRD in the final sample (**Figure S17**).

For the XRD, the starting sample of Cs_4PbBr_6 powder was mixed with a small amount of high vacuum silicone grease and compacted onto a zero diffraction Si wafer (**Figure S16**, left photo). After the reaction, the Cs_4PbBr_6-PMAO sample was centrifuged to separate solid phase from liquid, and a precipitated slurry was drop cast onto zero diffraction silicon wafer (**Figure S16**, right photo), in the latter case the residual PMAO holds the fine powder together.

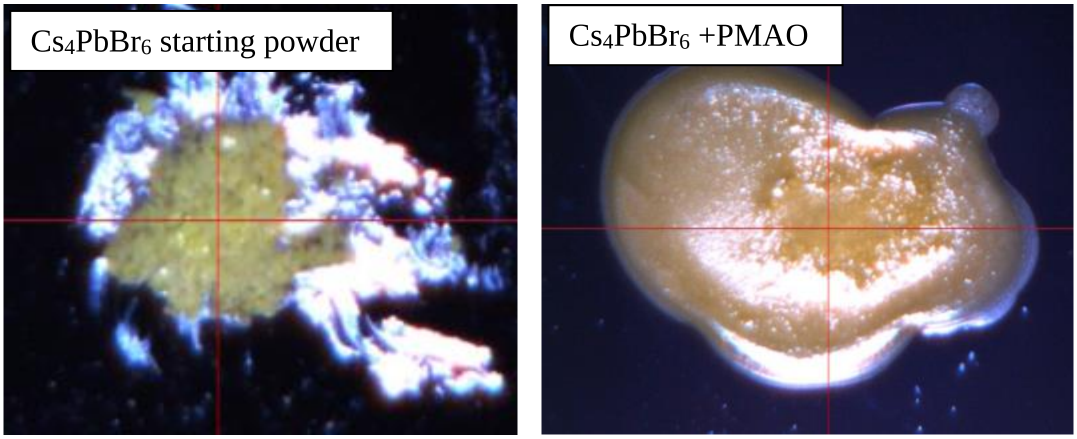

Figure S16. Photos of the XRD samples of the initial Cs_4PbBr_6 powder (left image) and the powder after the treatment with PMAO. The photos illustrate the change in the sample color from light green to light yellow after PMAO treatment.

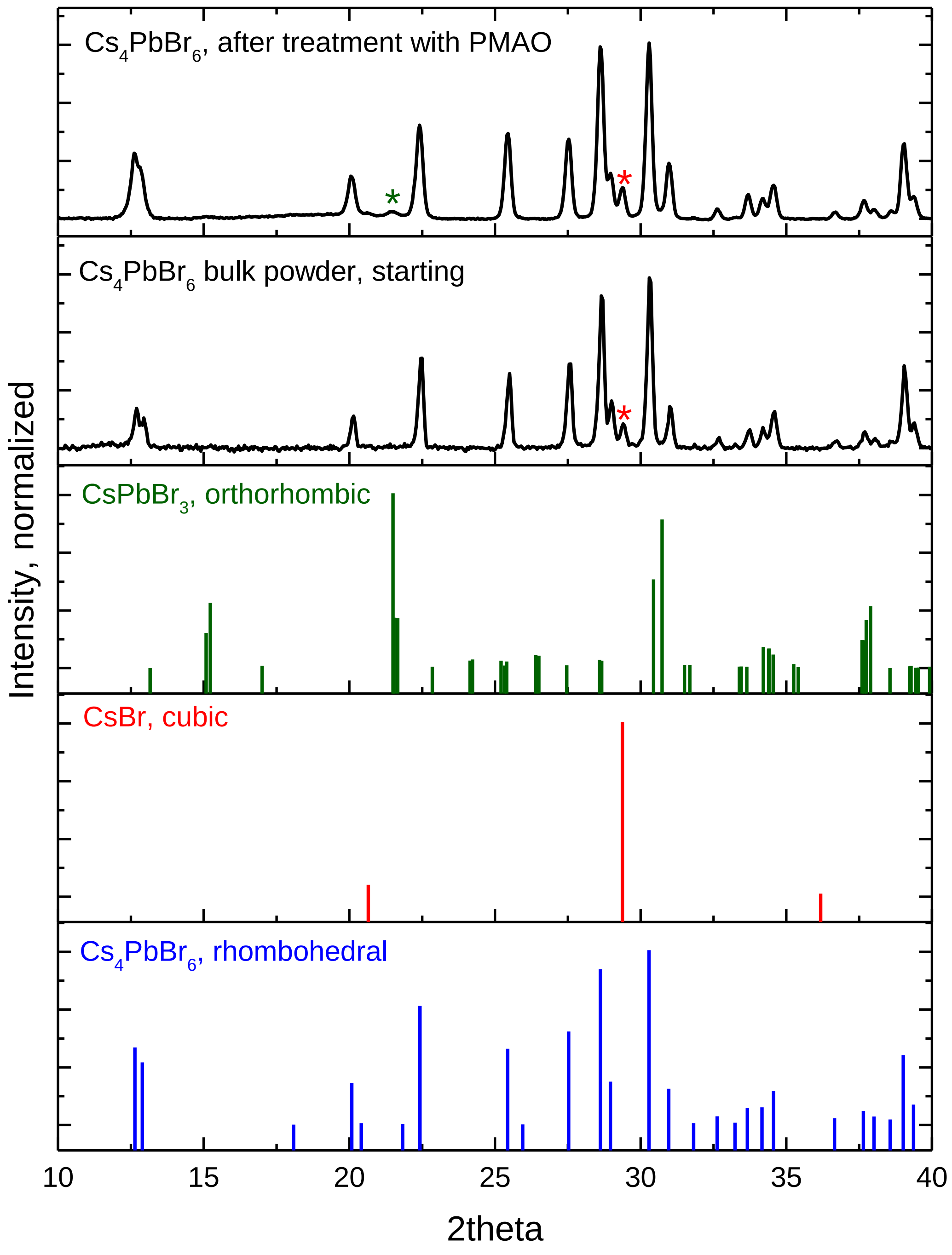

Figure S17. Experimental XRD patterns of Cs_4PbBr_6 bulk powder treated with PMAO and of the starting material (top two patterns), and the database reference patterns for Cs_4PbBr_6 (pattern ID 04-015-9683, ICSD code 162158, ref.[13]), CsBr (pattern ID 01-082-9638, ICSD code 236387, ref.[16]), and $CsPbBr_3$ (pattern ID 96-451-0746, COD code 4510745, ref.[14]). The peaks from CsBr and $CsPbBr_3$ in the experimental patterns are marked with red and green asterisks, respectively.

S8. FTIR and NIR absorbance spectra

Infrared spectra (**Figure S18**) of as-synthesized Cs_4PbBr_6 NCs evidenced the presence of oleate (peaks at ~1410 cm^{-1} and 1555 cm^{-1}) and oleylamine/oleylammonium species (a broad peak at ~3440 cm^{-1}), and the absence of free oleic acid (no ν(C=O) stretch at ~1711 cm^{-1}). Optical absorption spectra of concentrated NC solutions in the near-infrared (**Figure S19**) were consistent with these findings. No evidence of water or Pb-OH moieties[17-19] in NC samples was found by FTIR or optical absorption, despite performing the synthesis and handling samples in air.

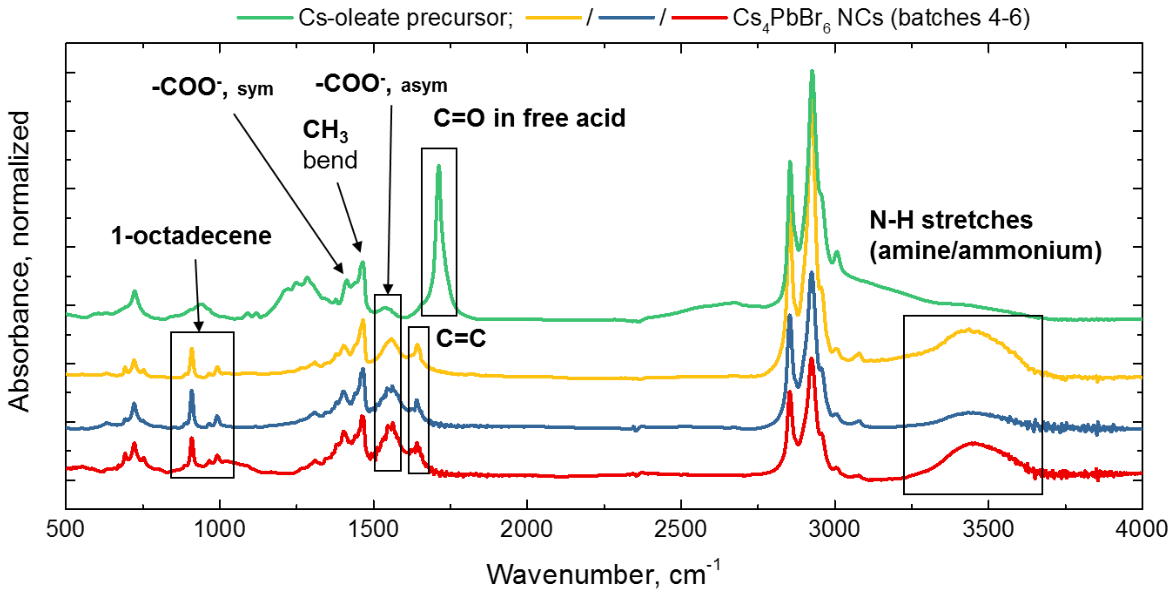

Figure S18. FTIR spectra of cesium oleate precursor (top trace) and three replica batches of Cs_4PbBr_6 NCs. The spectra were normalized to the amplitude of the CH_3 bend (~1464 cm^{-1}). The carbonyl stretch (C=O, ~1711 cm^{-1}) of free oleic acid is clearly observable in the cesium oleate precursor due to the excess of oleic acid but is absent in NC samples. A very broad asymmetric peak at ~3400-3500 cm^{-1} is interpreted as an oleylamine/oleylammonium mixture. The assignment of the peaks is based on prior works.[20, 21]

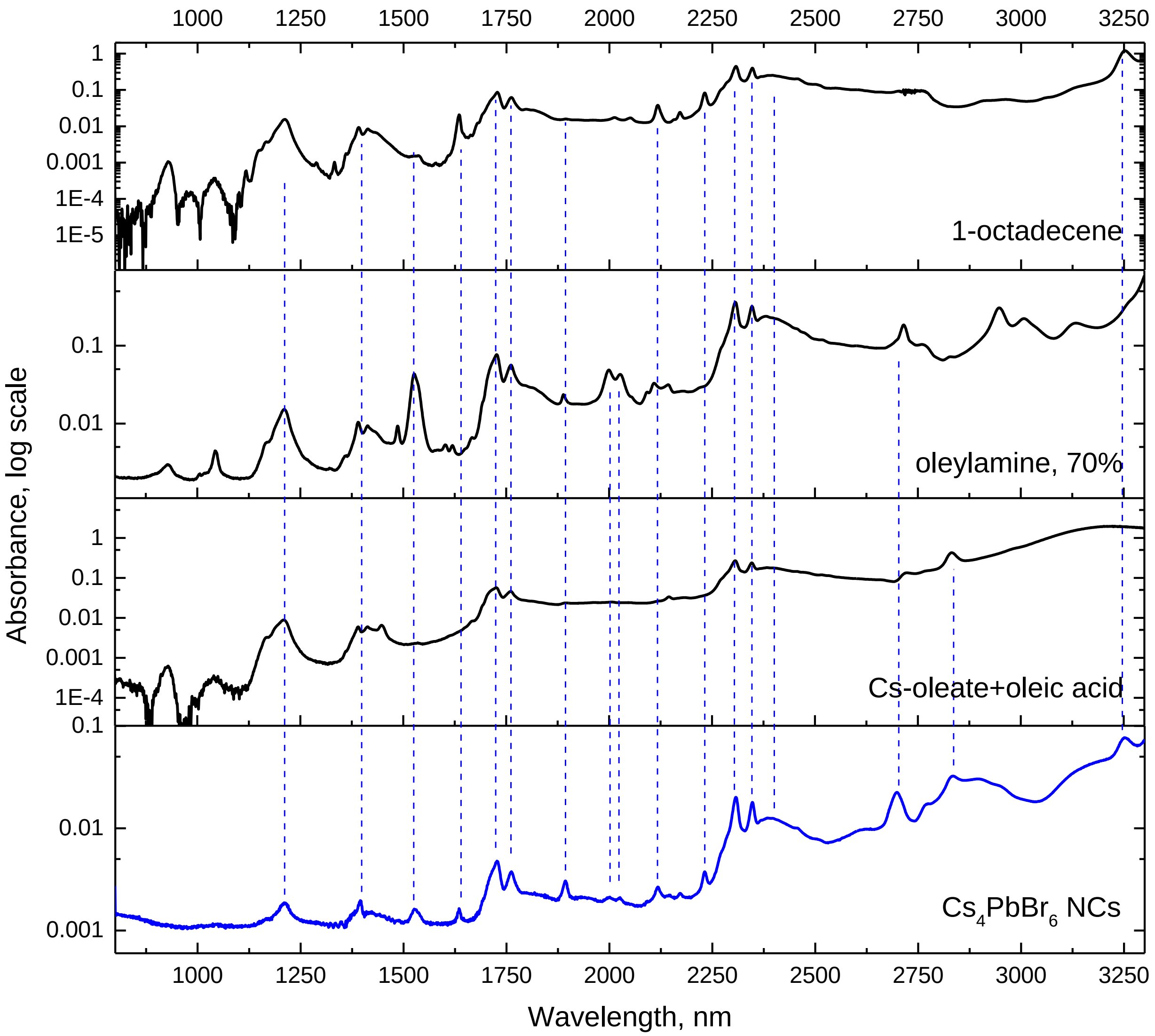

Figure S19. NIR (800-3300 nm) absorbance spectra of tetrachloroethylene solutions of Cs_4PbBr_6 NCs, cesium oleate precursor, oleylamine (oleylamine, tech. grade 70%, Aldrich), and 1-octadecene. Vertical dashed lines indicate the correspondence of the absorbance peaks in NCs with the peaks from the samples of organics.

S9. ^{1}H NMR investigation of Cs_4PbBr_6-PMAO reactivity

The reaction between oleylamine and PMAO leads to the broadening of the vinyl hydrogens resonance of oleylamine in the ^{1}H NMR spectrum (**Figures S20-S22**, sharp multiplet at 5.44 ppm of free oleylamine broadens and shifts to ~5.33 ppm in PMAO- oleylamine adduct), as discussed in the main text. The addition of oleylamine to cyclic anhydride yields an amic acid derivative, as was confirmed in a control reaction between oleylamine and succinic anhydride in DMSO-d_6 at room temperature (see figure **Figure S23** for ^{1}H and ^{1}H-^{13}C HSQC NMR spectra). The DMSO-d_6 was chosen as a solvent due to poor solubility of succinic anhydride in toluene-d_8. The succinic anhydride was used as a small molecule proxy of the reactive functional group of PMAO to avoid line broadening in NMR caused by the macromolecules. Therefore, it is assumed that PMAO- oleylamine adduct is a polysuccinamic acid in the rest of the discussion.

Note: commercial samples of PMAO contain a likely impurity of residual 1-octadecene monomer (**Figure S24**). Purification of the starting PMAO reagent was not attempted, but the final $CsPbBr_3$/PMAO NCs could be purified (washed) from 1-octadecene by several precipitation/redispersion cycles with ethyl acetate/toluene antisolvent/solvent pair (**Figure S25**).

Next, the reaction between PMAO and Cs_4PbBr_6 NCs was investigated by inspecting the ~5.3-5.9 ppm region of vinylic hydrogens in ^{1}H NMR spectra of two mixtures: transformed $CsPbBr_3$ NCs in a crude reaction mixture and after one washing cycle. The spectra were compared with ^{1}H NMR spectra of the starting Cs_4PbBr_6 NCs (**Figure S20**, see **Figures S21, S22** for full spectra). The region of vinylic hydrogens was chosen because it allows us to distinguish between bound and free states of the oleylamine and oleate ligands. The starting Cs_4PbBr_6 NCs show a single broad peak at ~5.49 ppm (**Figure S20**, "OLAM+OLAC bound") assigned to the overlapping resonances of vinyl hydrogens of oleylamine (OLAM) and oleate (OLAC) from the ligand shell of the NCs. After the addition of PMAO and a complete $Cs_4PbBr_6 \rightarrow CsPbBr_3$ transformation (confirmed by the absence of 314 nm peak in UV-Vis absorption spectra), the ^{1}H NMR spectrum of the crude reaction mixture revealed that a single broad peak of OLAM/OLAC ligands had been split in two: an even broader peak at ~5.55 ppm and a sharp multiplet at 5.47 ppm [**Figure S20**, "$CsPbBr_3$/PMAO (transformed, not washed)"]. The broad peak at ~5.55 ppm was assigned to the PMAO-oleylamine adduct based on control discussed above, while a multiplet at 5.47 ppm was assigned to the unbound ligands displaced from the NC surface. The ratio of the areas under the broad (~5.55 pm) and the sharp (5.47 ppm) peaks was estimated to be ~1.8 (via the "Line Fitting" tool in MestReNova, software ver. 12.0.0-20080). This ratio is very similar to ~3:2 molar ratio of (oleylamine+oleylammonium):oleate in the starting Cs_4PbBr_6 NCs. That similarity led us to the interpretation that a sharp multiplet at 5.47 ppm most likely belongs to oleate species displaced from the NC surface by a polysuccinamic acid formed as a result of oleylamine addition to PMAO.

The removal of oleate from the NC surface was further supported by two experiments: i) a comparison between the ^{1}H NMR spectra of the $CsPbBr_3$/PMAO NCs before and after washing with ethyl acetate. The washing purified the sample from unbound species (excess of polymer and displaced ligands) as evidence by a single broad peak at ~5.57 ppm corresponding to the PMAO-oleylamine adduct [**Figure S20**, "$CsPbBr_3$/PMAO (transformed, washed)"]; ii) dissolution of washed $CsPbBr_3$/PMAO in DMSO-d_6/TFA mixture. ^{1}H NMR spectrum of the dissolved $CsPbBr_3$/PMAO NCs only contained signatures of oleylammonium, and oleic acid was absent (**Figure S26**).

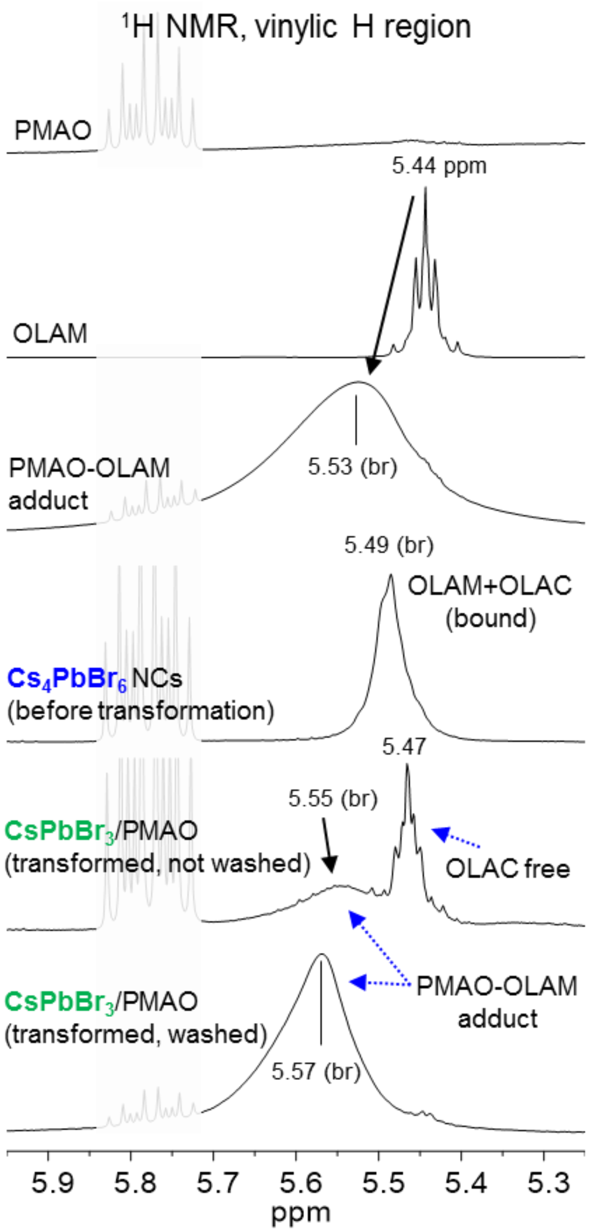

Figure S20. ^{1}H NMR spectra of vinylic hydrogens for solutions of reagents and NC samples in toluene-d_8. From top to bottom: poly(maleic anhydride-alt-1-octadecene) (PMAO), oleylamine (OLAM), PMAO-OLAM adduct (~1:1 anhydride to OLAM molar ratio; br = broad), Cs_4PbBr_6 NCs before the addition of PMAO (OLAC stands for oleate), fully-transformed NCs ($CsPbBr_3$/PMAO) before washing, and $CsPbBr_3$/PMAO NCs washed after the transformation by precipitation with ethyl acetate. Multiplets from residual 1-octadecene in the 5.85-5.7 ppm range are shaded.

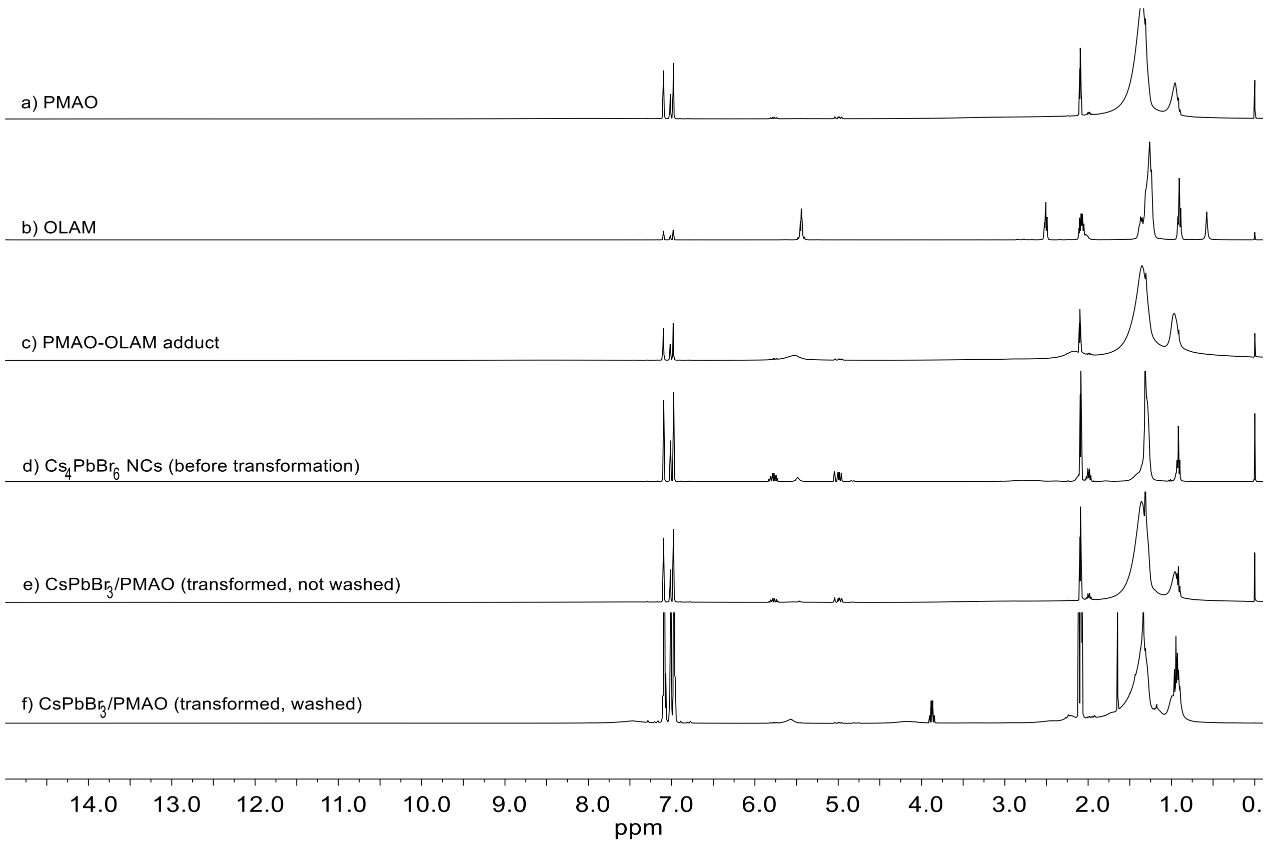

Figure S21. Full range ^{1}H NMR spectra of solutions in toluene-d_8: a) PMAO (Sigma-Aldrich), b) oleylamine (OLAM), c) PMAO-OLAM adduct (equimolar mixture), d) Cs_4PbBr_6 NCs before transformation, e) crude mixture of fully-transformed $CsPbBr_3$ NCs after PMAO was added to Cs_4PbBr_6 NCs [$CsPbBr_3$/PMAO (transformed, not washed)], f) washed $CsPbBr_3$ NCs after the transformation [$CsPbBr_3$/PMAO (transformed, washed)]. Peak at 0 ppm (a-e) is from internal standard TMS.

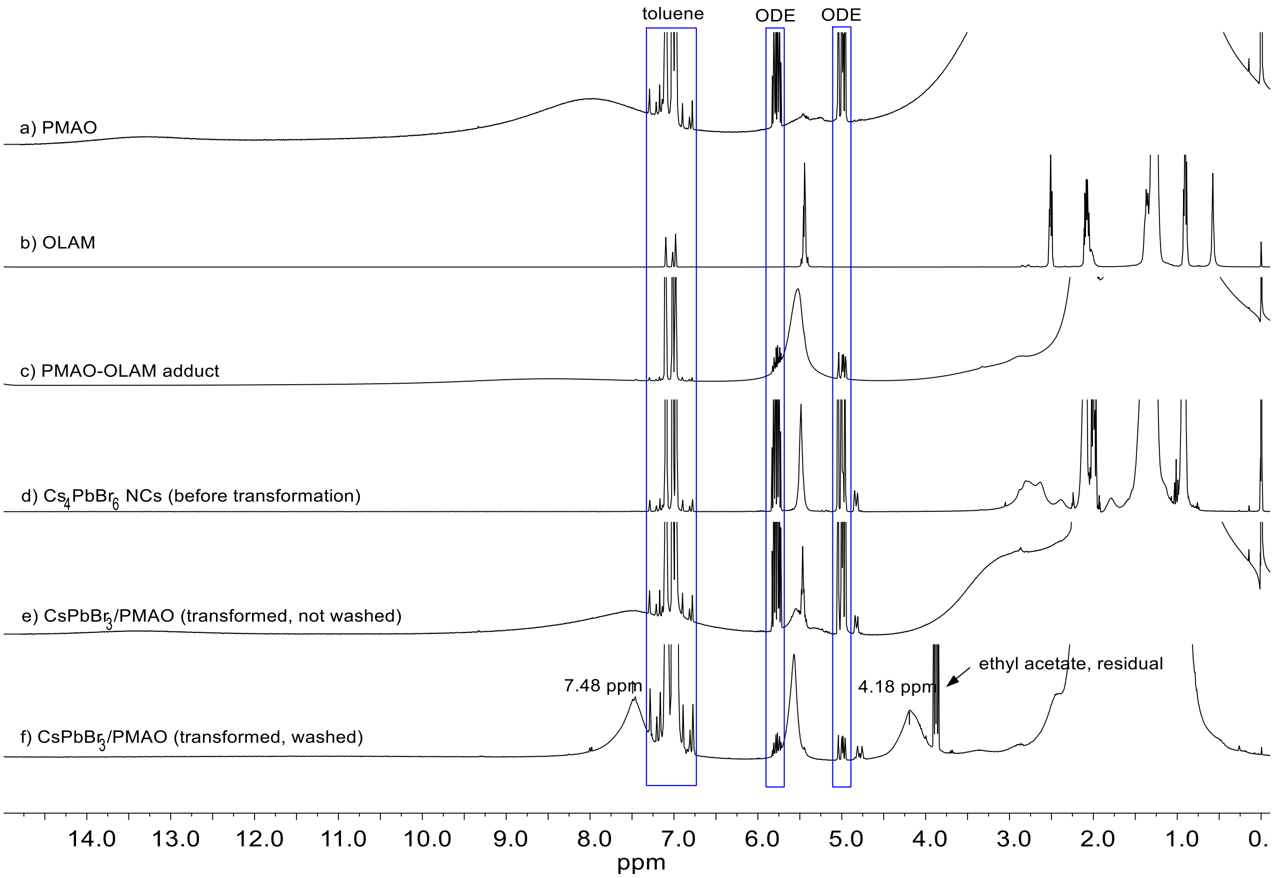

Figure S22. Low-intensity region of the full range ^{1}H NMR spectra shown in the preceding figure. In the spectrum of washed $CsPbBr_3$/PMAO NCs (panel f), the broad peaks at ~7.48 ppm and ~4.18 ppm are assigned to the **H**-N in the amide and an adjacent α-C$\mathbf{H_2}$- group in analogy with the succinamic acid control experiment [**Figure S23**, atom number 11, and "alpha CH_2 (OLAMIDE)"]. The upfield shift of the **H**-N peak (Δ~0.27 ppm) and a downfield shift of α-C$\mathbf{H_2}$ peak in polysuccinamic acid (Δ~-1 ppm) were observed.

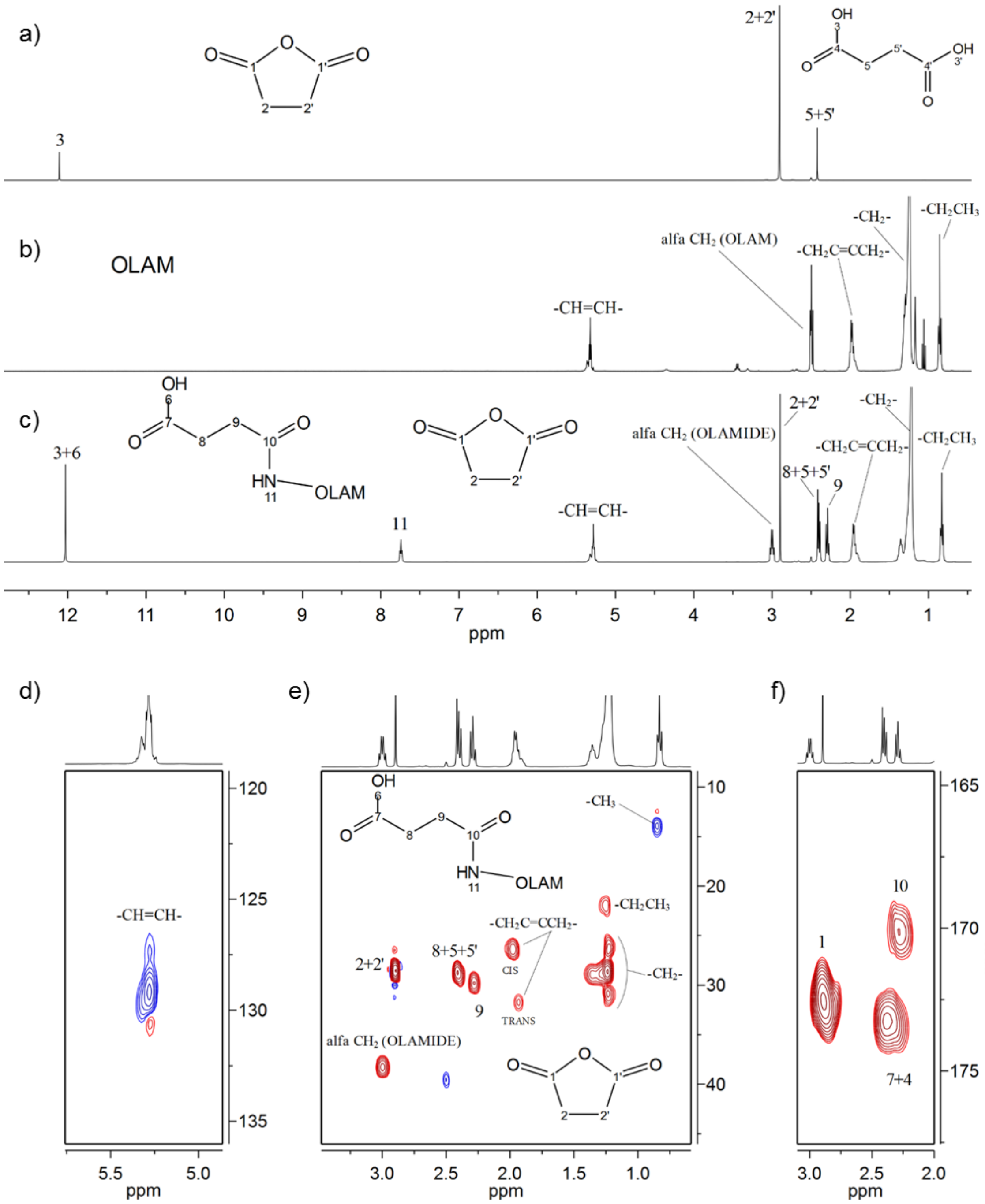

Figure S23. NMR spectra of a control reaction between excess of succinic anhydride and oleylamine (OLAM) in DMSO-d_6: a) ^1H NMR of succinic anhydride; b) ^1H NMR of oleylamine; c) ^1H NMR of the mixture of succinic anhydride and OLAM. The signal at ~12 ppm in panel a) is an impurity of succinic acid present in the starting material; d-e) ^1H-^{13}C HSQC NMR spectra and f) ^1H-^{13}C HMBC NMR spectrum of selected regions for the mixture of succinic anhydride and OLAM.

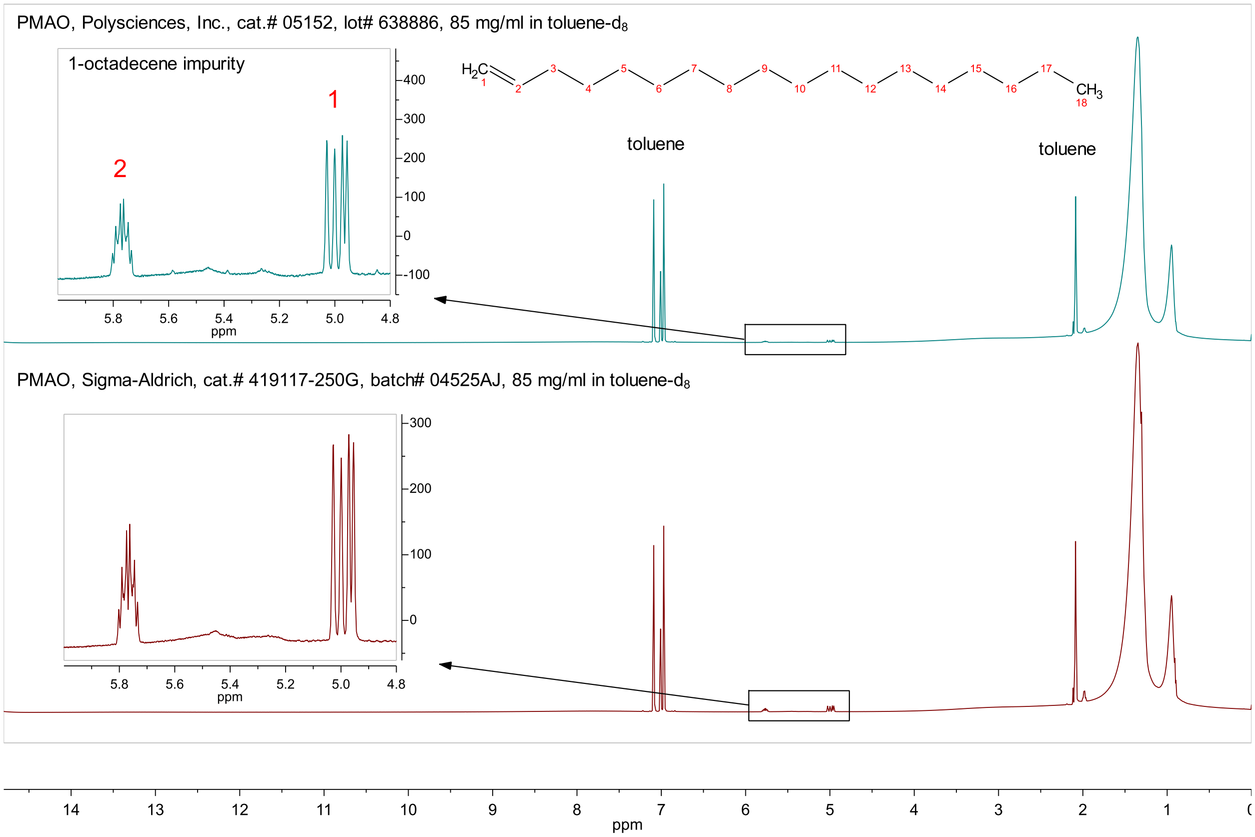

Figure S24. Comparison of ^{1}H NMR spectra of two commercial PMAO reagents (85 mg/ml solutions in deuterated toluene). The insets show a zoomed-in region of vinylic protons. The sharp multiplets at ~5.76 ppm and ~5.02-4.96 ppm were observed in ^{1}H NMR spectra of two commercial PMAO samples and assigned to vinylic protons of 1-octadecene impurity based on prior 1-octadecene characterization by McPhail and Weiss.[22] We hypothesize that this impurity is either a residual 1-octadecene monomer left after PMAO manufacturing or an unidentified side product/unsaturated hydrocarbon. Luckily, 1-octadecene is inert to Cs_4PbBr_6 and $CsPbBr_3$ at room temperature and does not appear to play a role in the chemical transformation.

The sample of $CsPbBr_3$/PMAO NCs washed once still contains a residual 1-octadecene (e.g. **Figures S20**, bottom spectrum). The residual 1-octadecene can be removed by repeated precipitation/redispersion of $CsPbBr_3$/PMAO NCs with ethyl acetate (for precipitation) and toluene (for redispersion). For example, four consecutive purifications reduced the amount of 1-octadecene in the sample to the amounts below the detection limit of the ^{1}H NMR experiment (**Figure S25**). The $CsPbBr_3$/PMAO NC sample washed four times is sufficiently stable to perform an NMR experiment.

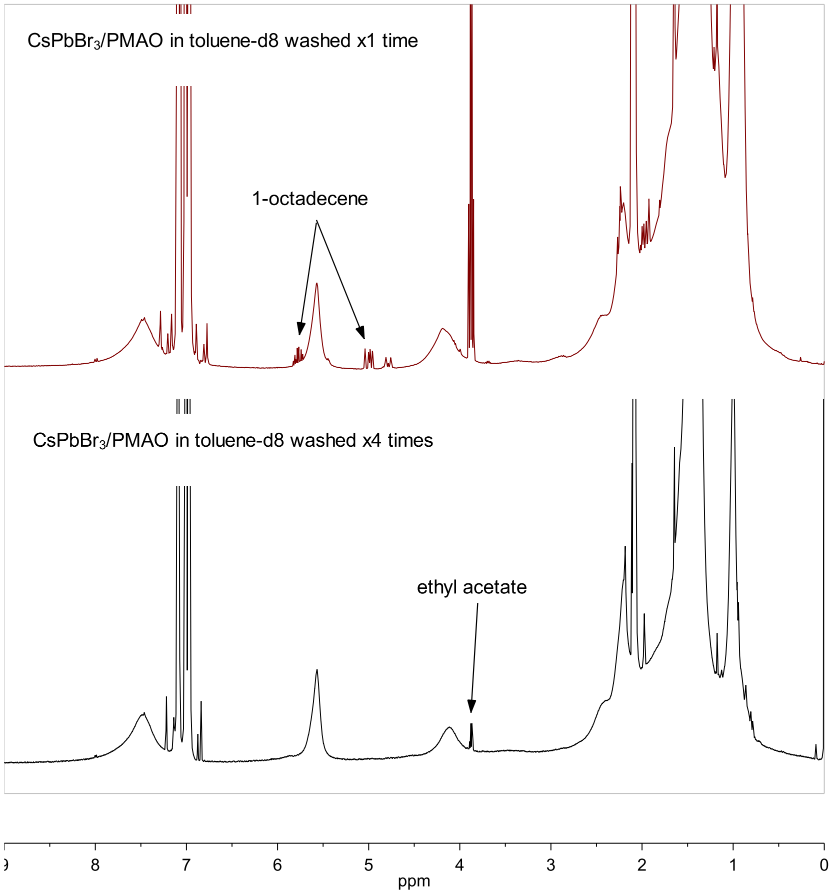

Figure S25. Comparison of the ^{1}H NMR spectra of $CsPbBr_3$/PMAO NCs after a single cycle of precipitation/redispersion with ethyl acetate and toluene (top spectrum) and after four cycles (bottom spectrum). The consecutive purification reduces the amounts of residual 1-octadecene to below the detection limit.

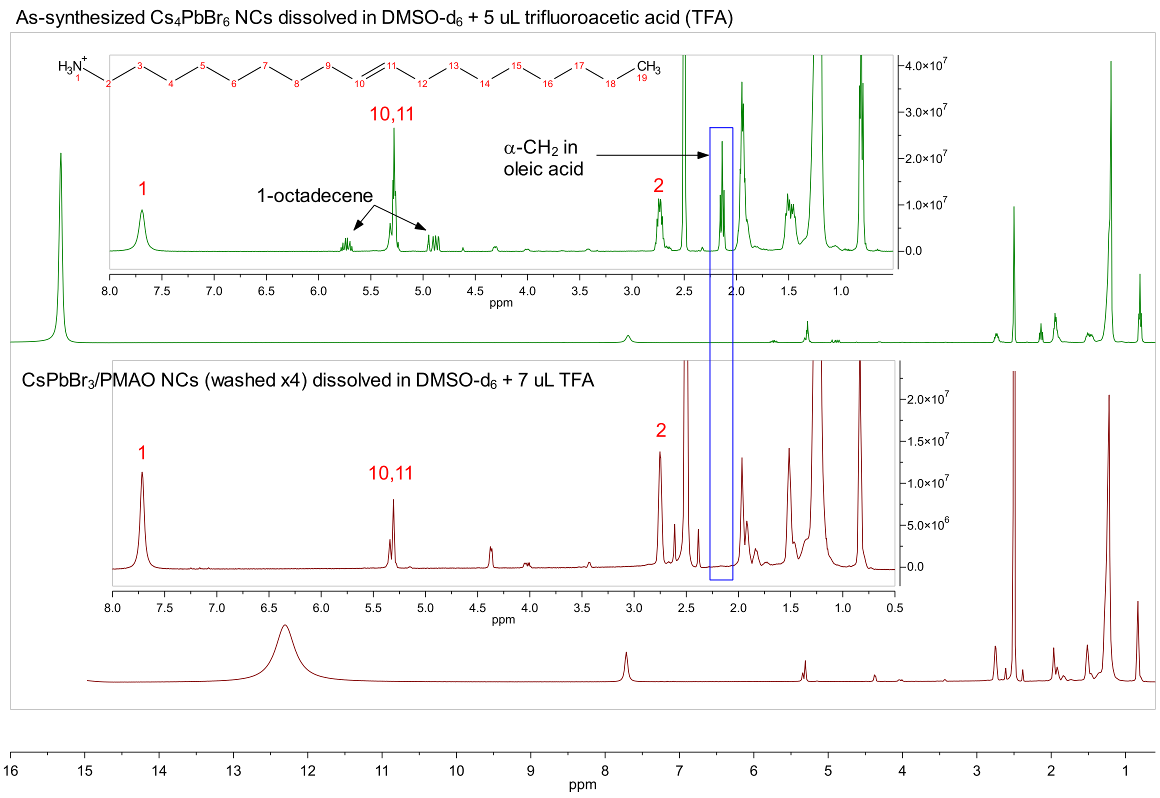

Figure S26. Comparison of ^{1}H NMR spectrum of the starting Cs_4PbBr_6 NCs dissolved in DMSO-d_6/TFA (green curve, data reproduced from **Figure S9**) with a spectrum of washed (x4 times) $CsPbBr_3$/PMAO NCs dissolved in DMSO-d_6/TFA (maroon curve). The spectrum of the dissolved Cs_4PbBr_6 NCs shows both oleylammonium and oleic acid species (α-C**H_2** multiplets at ~2.72 ppm, and ~2.14 ppm, respectively). The spectrum of the dissolved $CsPbBr_3$/PMAO NCs shows oleylammonium species due to the acid hydrolysis of PMAO-oleylamine adduct by TFA. However, the characteristic signal from oleic acid, α-C**H_2** peak at ~2.14 ppm, is absent in the spectrum of the dissolved $CsPbBr_3$/PMAO sample. That further confirms that oleic acid was removed from the final washed NCs.

S10. Control reaction between amine-free Cs_4PbBr_6 NCs and PMAO

Oleylamine-free Cs_4PbBr_6 NCs were synthesized using the tri-n-octylphosphine oxide (TOPO) instead of oleylamine following the previously described procedure.[23] The resulting NCs are larger in size, so they scatter in the optical spectra, consistent with the original *JACS* report.[23] PMAO was dissolved in anhydrous toluene to form an 85 mg/ml concentrated solution.

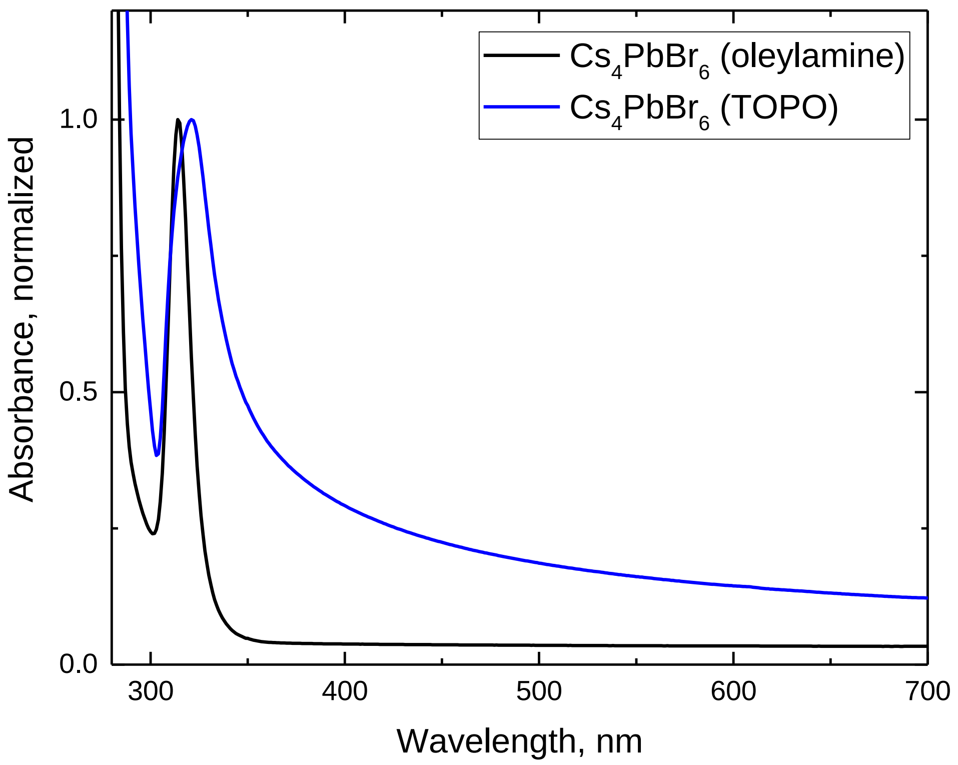

Figure S27. Comparison of the optical absorption spectra of Cs_4PbBr_6 NCs synthesized via oleylamine and TOPO routes. The TOPO-based ones are larger in size, which results in the increased contribution from Rayleigh scattering to the spectrum.

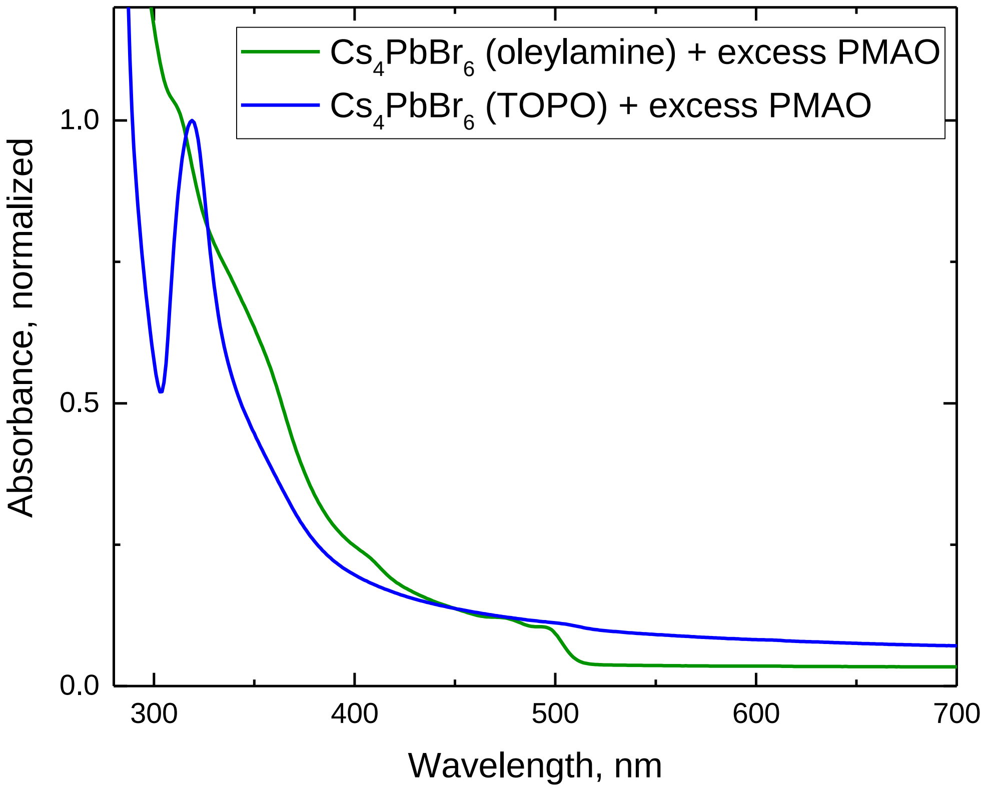

Figure S28. Optical absorption spectra of Cs_4PbBr_6 NCs from oleylamine and TOPO syntheses after reaction with an excess of PMAO. The Cs_4PbBr_6 NCs synthesized via the oleylamine route are fully converted to $CsPbBr_3$, while Cs_4PbBr_6 NCs synthesized via TOPO route have barely reacted. The reaction was conducted under heating at 80 ^{o}C. The initial concentration of Cs_4PbBr_6 between the two samples was assumed equal by setting the absorbance value at the absorption maxima between the two Cs_4PbBr_6 NC solutions at the same value through dilution of one of the samples. The same volume of 85 mg/ml PMAO solution in toluene was added to each Cs_4PbBr_6 NC sample, and the total volume of the reaction between the two was kept similar.

S11. Stability of resulting $CsPbBr_3$/PMAO NCs

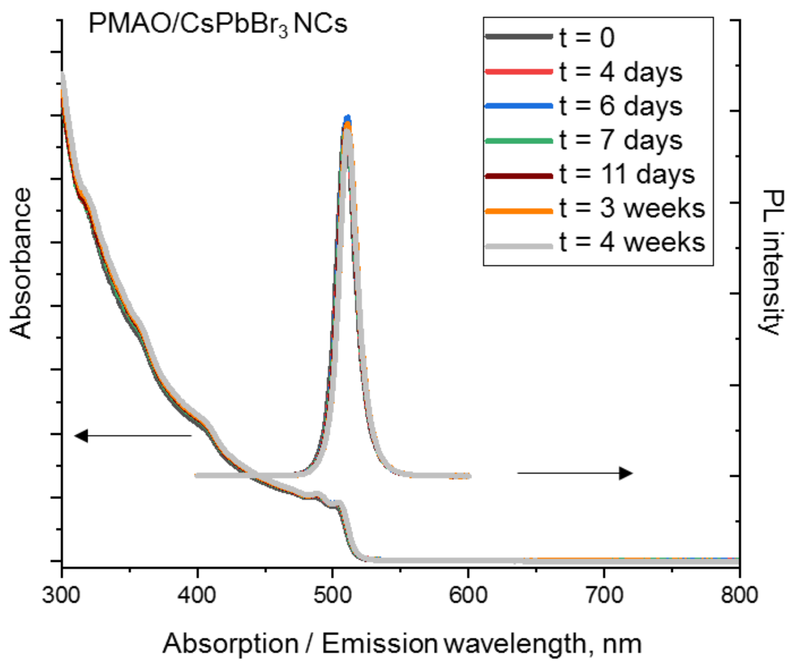

Figure S29. Absorption and PL spectra of fully-converted PMAO/$CsPbBr_3$ NCs over time while being stored under ambient conditions in the air.

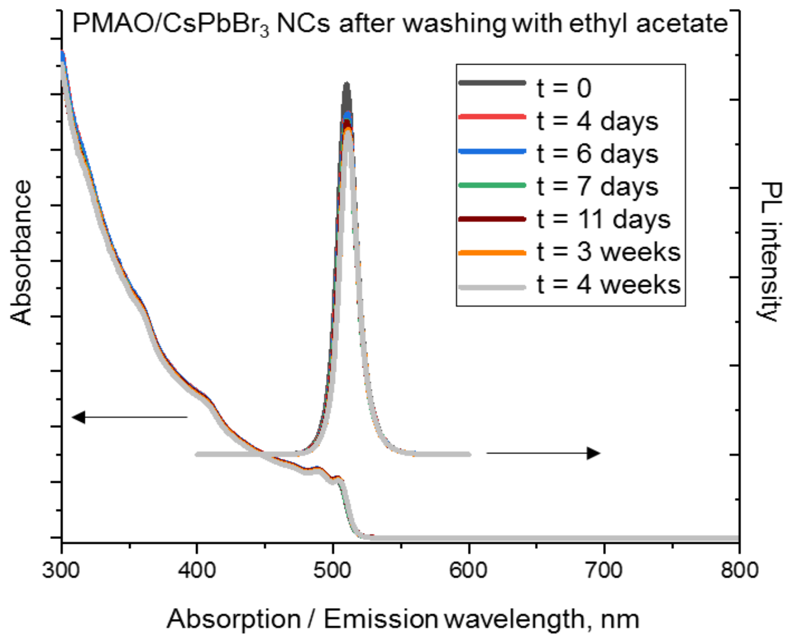

Figure S30. Absorption and PL spectra of fully-converted PMAO/$CsPbBr_3$ NCs (washed once with ethyl acetate) over time while being stored under ambient conditions in the air.

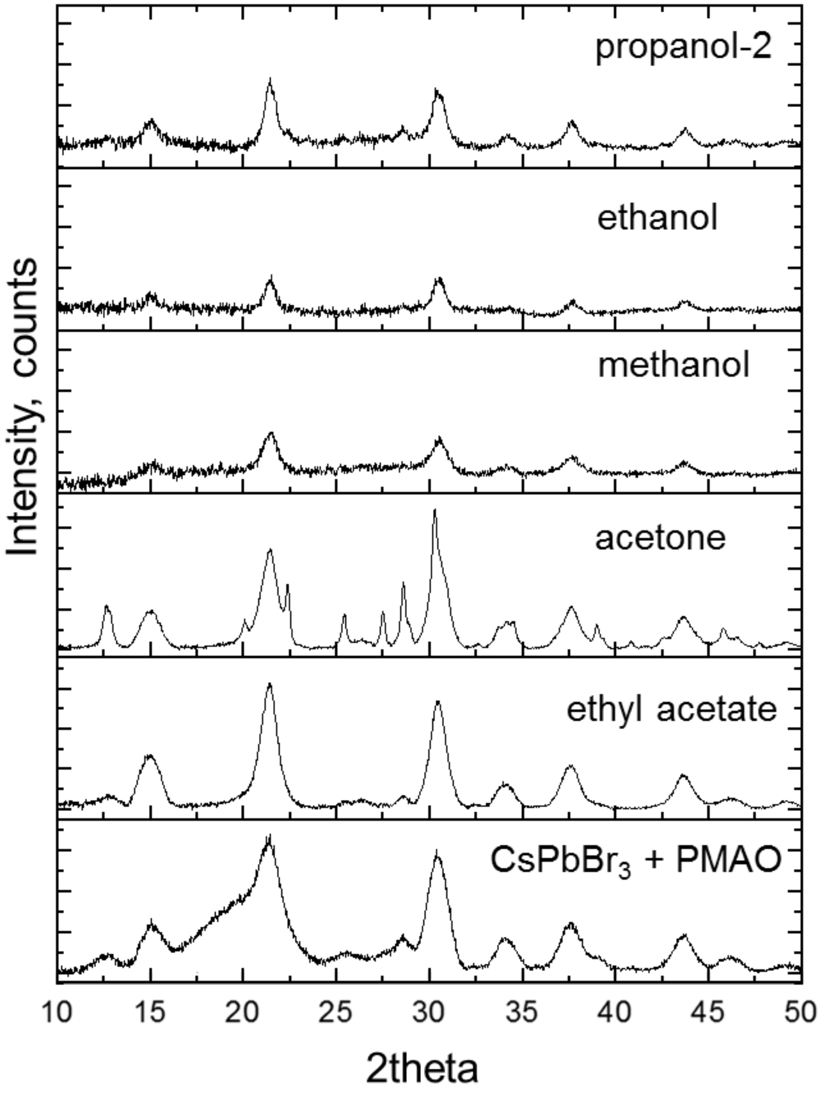

Figure S31. XRD patterns of (bottom panel) fully-transformed $CsPbBr_3$ NC sample with PMAO, and of the samples obtained from it by precipitation with five different polar solvents (top to bottom): propanol-2, ethanol, methanol, acetone, and ethyl acetate. Washing the $CsPbBr_3$+PMAO sample with acetone resulted in the NC aggregation and degradation, including partial back-conversion to Cs_4PbBr_6. Washing with other solvents did not appear to have such a severe effect.

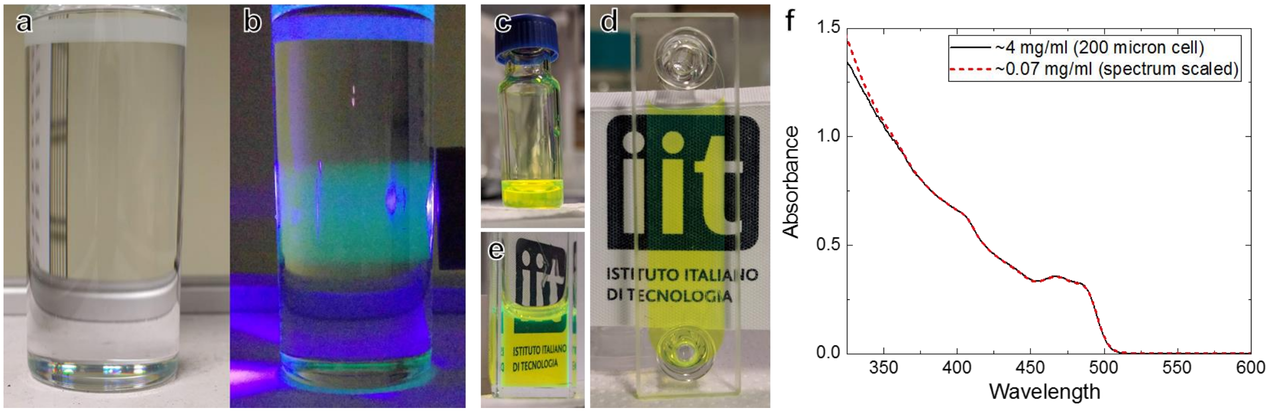

Figure S32. Diluted and concentrated solutions of $CsPbBr_3$/PMAO NCs in toluene: **a, b**) Dilute ~1×10^{-4} mg/ml solution under ambient and UV light (395 nm blue LED flashlight). The photos were acquired with a camera of LG Nexus 5X smartphone [exposure time 0.04 s, ISO-95 for the photo **a)**, 0.25 s, ISO-3900 for the photo **b)**] The dilute solution remained luminescent 2 weeks later (stored in a capped vial and wrapped in a piece of aluminum foil). **c)** Concentrated ~26 mg/ml solution of washed $CsPbBr_3$/PMAO NCs in a glass vial and in a **d)** 200 micron-thick optical cell, illustrating its transparency. The ~26 mg/ml concentration of $CsPbBr_3$ is within a factor of ~200 of the pure bulk $CsPbBr_3$ solid (calculated density of 4.83 g/cm^3).[24] **e)** An optically transparent ~4 mg/ml solution in a 10 mm-thick quartz cuvette. **f)** The optical absorption spectrum of ~4 mg/ml solution measured through 200 micron-thick cell (solid black line) and an overlapped absorption spectrum of ~7×10^{-2} mg/ml solution measured through 10 mm-thick cuvette (dashed red line, scaled up to match the absorbance value of the concentrated solution at 400 nm). The reported concentrations of $CsPbBr_3$ NCs were estimated optically by using a PL-based sizing curve of ref.[25] in conjunction with a molar extinction coefficient of $CsPbBr_3$ from ref.[26] and scaled with dilution as appropriate (for example, it was impossible to directly record absorption spectrum of ~26 mg/ml solution through the thinnest available cell with a known pathlength [200-micron thick cuvette (**d**)] due to the very strong light attenuation by the NC solution (optical density at 500 nm, i.e., below the band edge, was already ~1.6), so the concentrated solution was diluted and the initial concentration back-calculated using the known dilution factor).

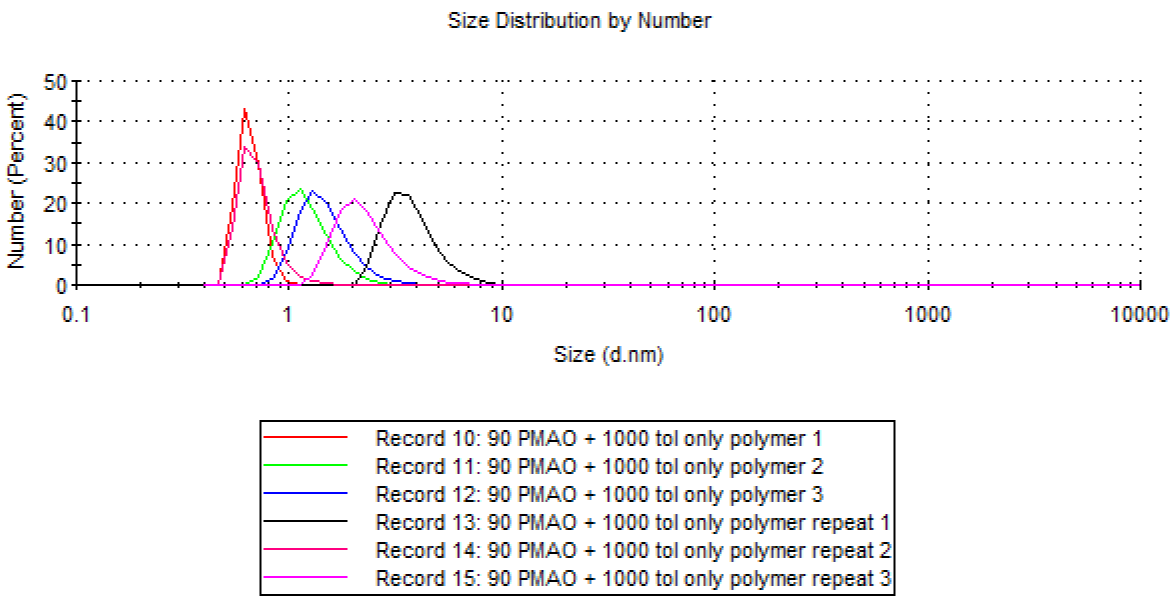

Figure S33. Dynamic light scattering (DLS) number distribution for 6 measurements of PMAO only in toluene, the average ± standard deviation of the six measurements is 1.7±1.2 nm. The measurements were conducted at 25°C with a Malvern Zetasizer Nano collecting the scattered light at 173° (backscattering mode). The light source was a HeNe laser (633 nm).

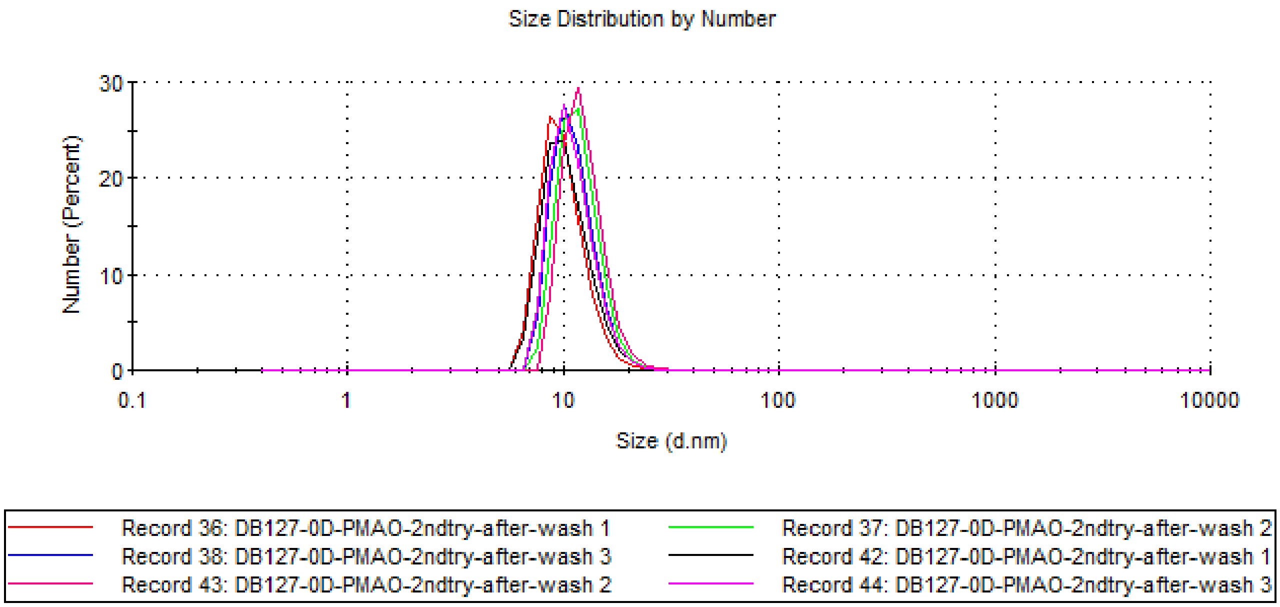

Figure S34. DLS number distribution for 6 measurements of the washed $CsPbBr_3$/PMAO NCs. The average ± standard deviation of the six measurements is 11.2±0.9 nm. The measurements were conducted at 25°C with a Malvern Zetasizer Nano collecting the scattered light at 173° (backscattering mode). The light source was a HeNe laser (633 nm).

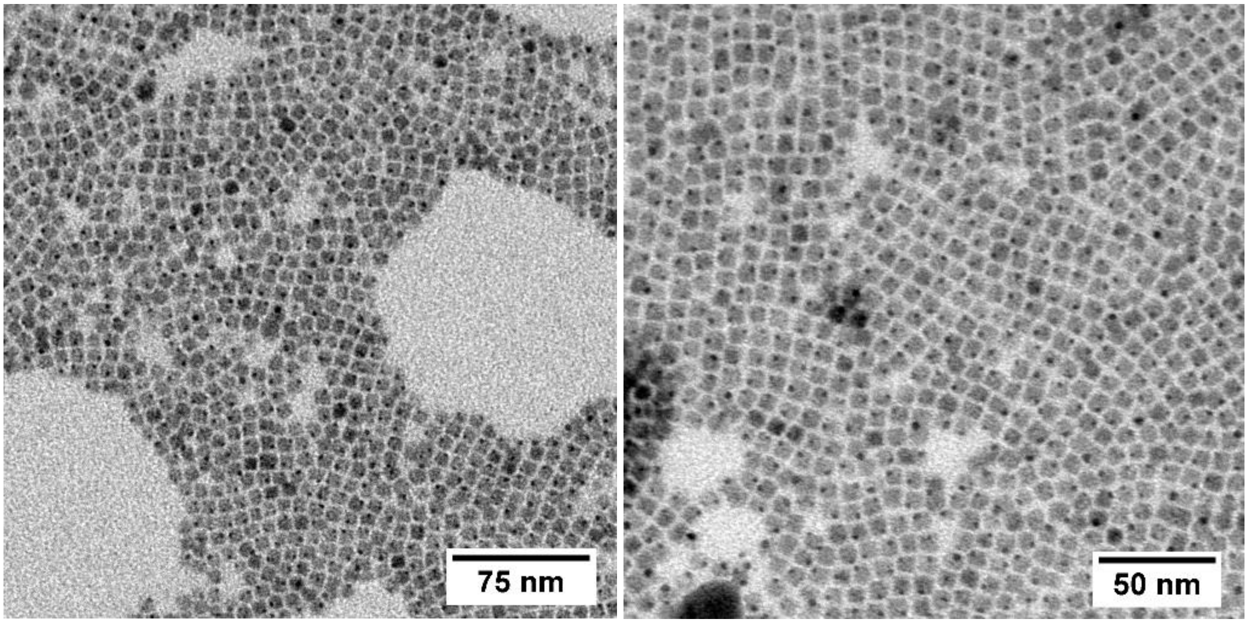

Figure S35. Low resolution TEM images of the washed $CsPbBr_3$/PMAO NCs deposited from the solution which was used for DLS. Size analysis of 5 images gave the following results (N particles, average edge length ± standard deviation):

179, 7.3±1.5 nm;
223, 7.4±1.7 nm
65, 7.2±1.6 nm;
576, 6.3±1.3
340, 7.2±1.5 nm;

S12. Additional HRTEM images

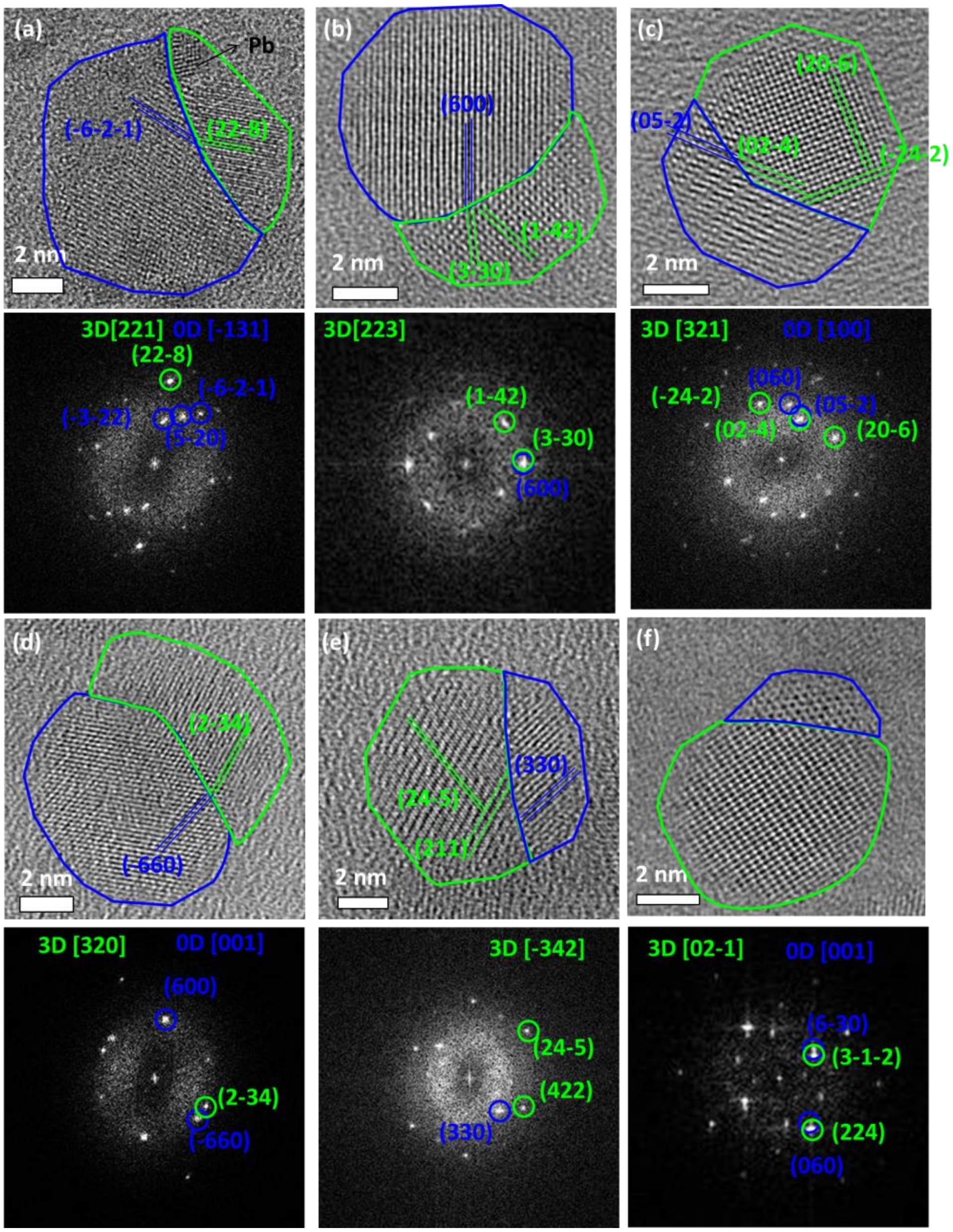

Figure S36. Annotated HRTEM images and corresponding FFT analysis of NC Cs_4PbBr_6-$CsPbBr_3$ heterostructures (green lines outline $CsPbBr_3$ domains, and blue lines outline Cs_4PbBr_6 domains). Panels a, b, c, and f reproduce heterostructures shown in **Figure 3**, panels a, b, d, and e in the main text. The heterostructure shown in (a) contains a domain of Pb (labeled by a black arrow) produced as a result of electron beam damage. The heterostructure shown in (f) contains two domains adopting epitaxial relationship, while the rest of the heterostructures do not show a consistent structural relationship between the two domains. In other cases, a set of $CsPbBr_3$ planes and Cs_4PbBr_6 planes adopt a small angle at the interface. This indicates that although these are not perfect epitaxial relationships or grain boundary conditions, the octahedral planes of two domains are connected at a small angle.

S13. TEM size analysis before and after transformation

a) ~10 nm Cs_4PbBr_6 NCs to ~8 nm $CsPbBr_3$ NCs

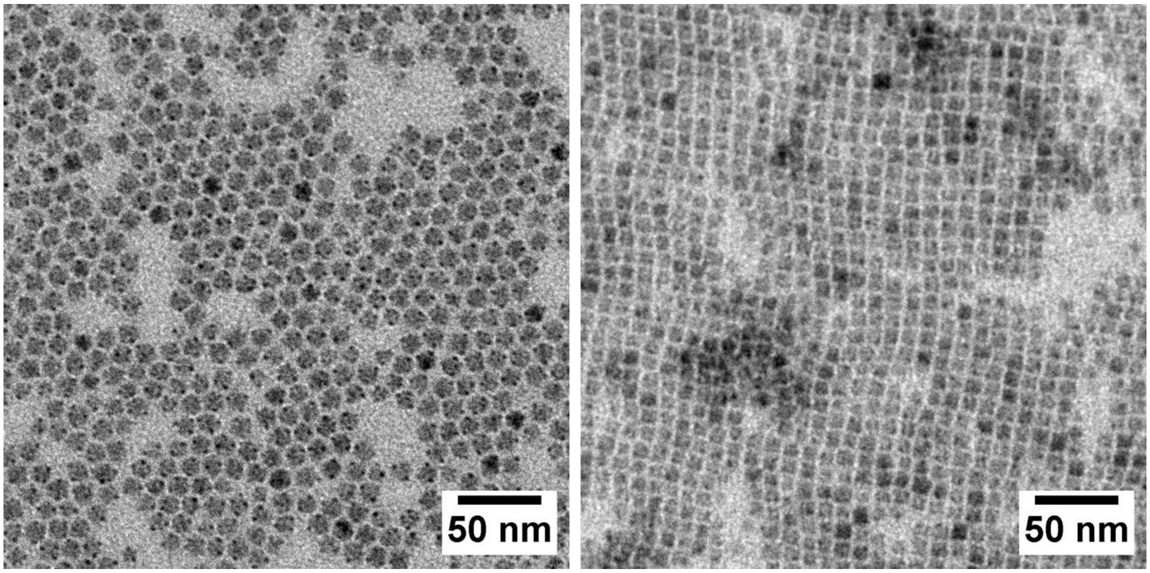

Figure S37. Side by side comparison of TEM images on the same scale of the starting Cs_4PbBr_6 NCs and resulting $CsPbBr_3$ NCs derived from them using PMAO-induced transformation.

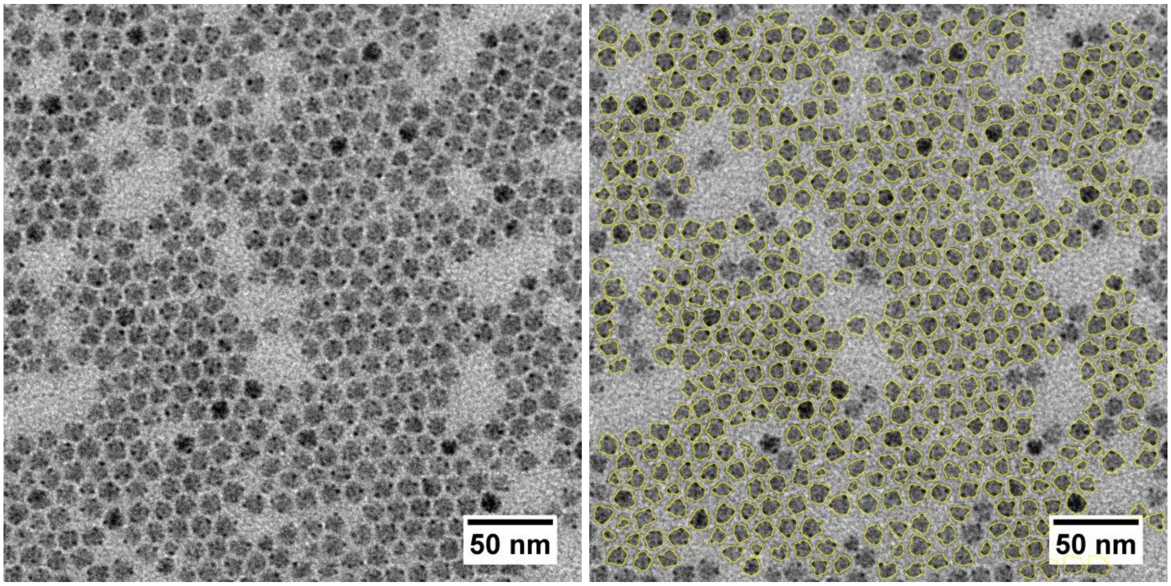

Figure S38. A representative low magnification TEM image of Cs_4PbBr_6 NCs (left image) and contours of NCs identified by the thresholding analysis (right image). The average circular area equivalent diameter ± standard deviation for this image is 10 nm ± 1.4 nm (473 NCs). Other images (not shown) yielded similar average diameters, e.g., 10 nm ± 1.3 nm (457 NCs), 10.2 nm ± 1.5 nm (936 NCs). The global average is 10.1 nm ± 1.4 nm.

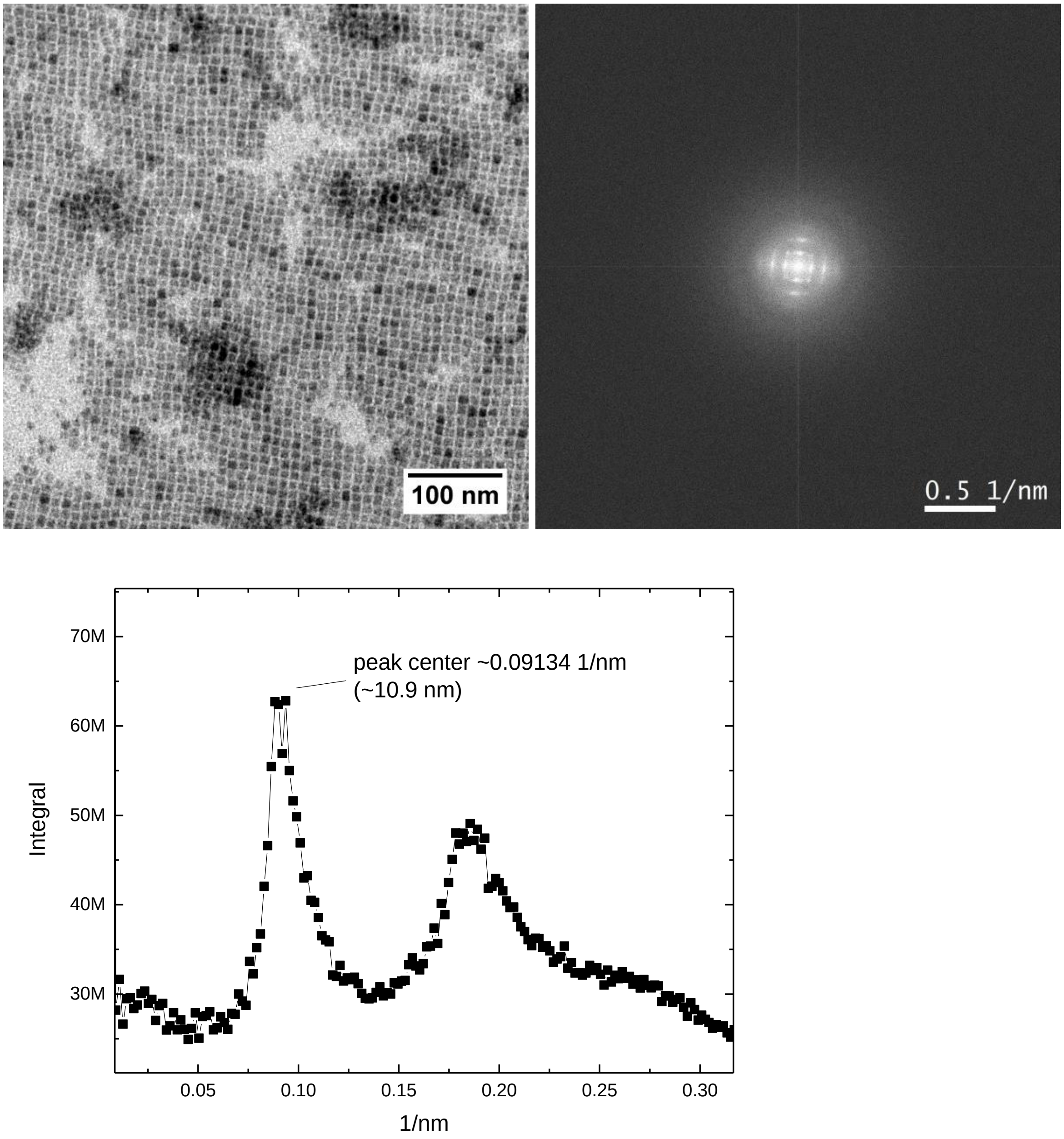

Figure S39. TEM photograph (upper left image) of $CsPbBr_3$ NCs formed from Cs_4PbBr_6 NCs by PMAO-induced transformation. Fast Fourier transform (FFT, upper right image) and it's azimuthal integral (lower left image). The FFT analysis takes advantage of close-packing of the $CsPbBr_3$ NCs and provides means to quantify their periodicity in space. The periodicity determined from FFT integral of the TEM photograph is ~10.9 nm, as indicated in the figure. Averaging the periodicity across 5 images yielded 10.8 nm ± 0.2 nm. That spatial period is interpreted as a sum of NC width and interparticle spacing.[6] The spacing between NCs of ~2.8 nm ± 0.4 nm was estimated from real space images using ImageJ. The difference between the abovementioned values gives an estimated value for the NC edge length of ~8 nm ± 0.4 nm.

b) ~16 nm Cs_4PbBr_6 NCs to ~12 nm $CsPbBr_3$ NCs

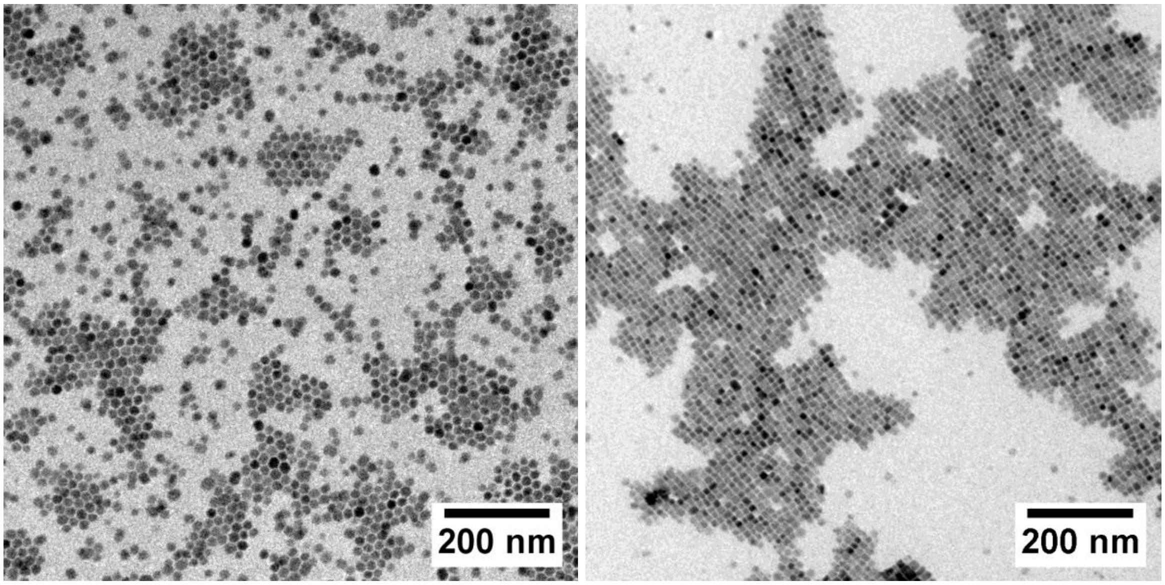

Figure S40. Side by side comparison of TEM images on the same scale of the starting Cs_4PbBr_6 NCs and resulting $CsPbBr_3$ NCs derived from them using PMAO-induced transformation.

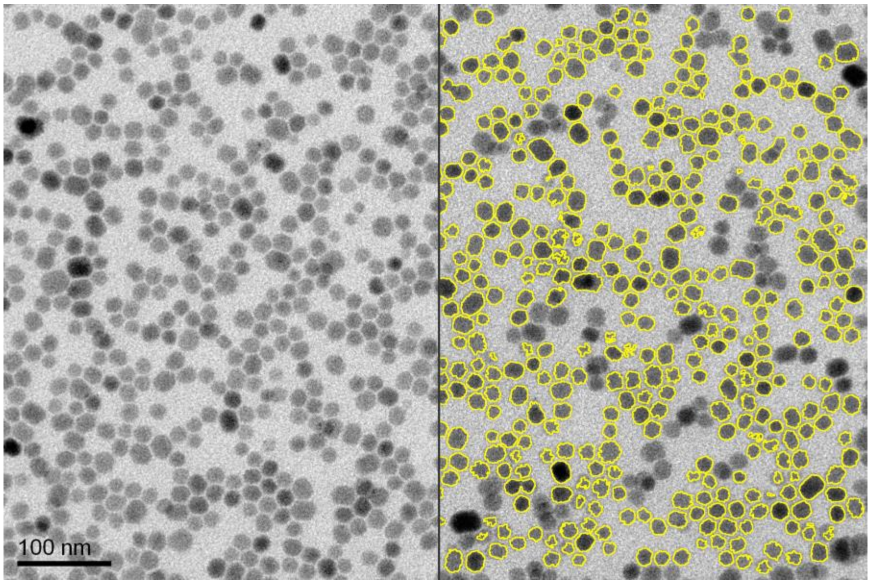

Figure S41. A representative low magnification TEM image of Cs_4PbBr_6 NCs (left image) and contours of NCs identified by the thresholding analysis (right image). The average circular area equivalent diameter for this sample was determined to be 15.7 nm ± 2.6 nm (total of 3357 NCs from three images).

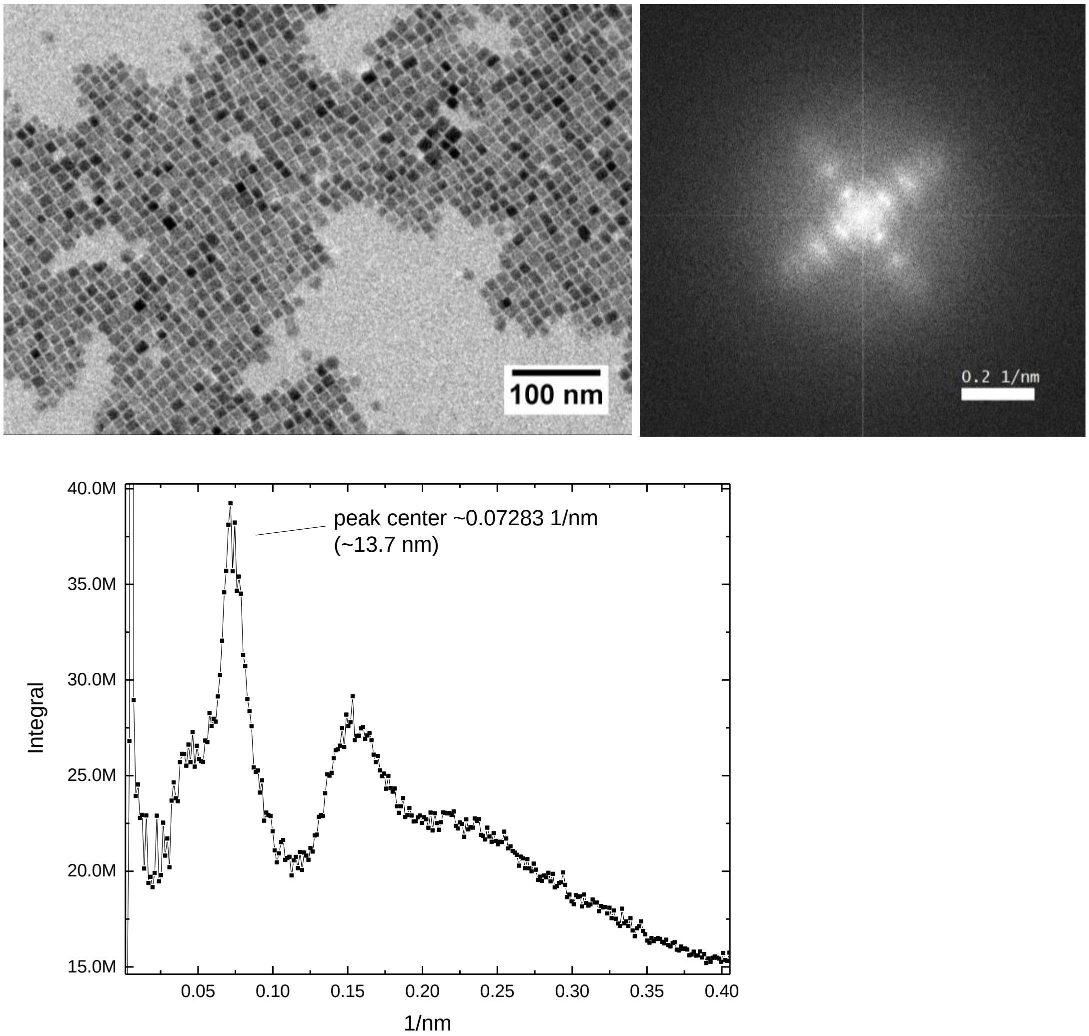

Figure S42. TEM photograph (upper left image) of $CsPbBr_3$ NCs formed from Cs_4PbBr_6 NCs by PMAO-induced transformation. Fast Fourier transform (FFT, upper right image) and it's azimuthal integral (lower left image). The FFT analysis takes advantage of close-packing of the $CsPbBr_3$ NCs and provides means to quantify their periodicity in space. The periodicity determined from FFT integral of the TEM photograph is ~13.7 nm, as indicated in the figure. Averaging the periodicity across 9 images yielded 14.2 nm ± 1.8 nm. That spatial period is interpreted as a sum of NC width and interparticle spacing.[6] The spacing between NCs of ~2.2 nm ± 0.5 nm was estimated from real space images using ImageJ. The difference between the abovementioned values gives an estimated value for the NC edge length of ~12 nm ± 1.9 nm.

c) Calculations of conversion stoichiometry

The NC shapes are assumed to be a sphere for Cs_4PbBr_6 NCs and a cube for $CsPbBr_3$ NCs. Another assumption is that the transformation happens one for one, i.e., one NC Cs_4PbBr_6 transforms into one NC of $CsPbBr_3$.

A sphere of bulk Cs_4PbBr_6 with a diameter of 10.1 nm has a volume of:

$$V_{NC\ Cs_4PbBr_6} = \frac{4}{3}\pi\left(\frac{10.1\ nm}{2}\right)^3 \approx 540\ nm^3,$$

And, using the calculated bulk density $\rho_{Cs_4PbBr_6} = 4.299$ g/cm^3,[13] $M_{w\ Cs_4PbBr_6} = 1218.29$ g/mol, contains a number of moles of Cs_4PbBr_6:

$$n_{Cs_4PbBr_6/NC} = V_{NC\ Cs_4PbBr_6} \cdot \rho_{Cs_4PbBr_6} / M_{w\ Cs_4PbBr_6} = 1.904 \cdot 10^{-21} \text{ moles},$$

Table S1 summarizes calculated cube edges for $CsPbBr_3$ NC, assuming 1 equiv. Cs_4PbBr_6 converts to n equiv. of $CsPbBr_3$, and using $\rho_{CsPbBr_3} = 4.86$ g/cm^3 (ref. [14]) and $M_{w\ CsPbBr_3} = 579.82$ g/mol for a reverse calculation from the number of moles to the NC cube dimensions.

Table S1. One to one Cs_4PbBr_6 to $CsPbBr_3$ NC conversion calculations. Values in bold are the ones in agreement with the interpretation of TEM results.

Sphere (Cs_4PbBr_6), average diameter 10.1 nm (experimental) to Cube ($CsPbBr_3$), average edge length 8 nm (experimental)					
1 equiv. Cs_4PbBr_6 to *n* equiv. of $CsPbBr_3$	n = 1	n = 2	**n = 2.25**	n = 3	n = 4
Cube edge length, calculated, nm	6.1	**7.7**	**8.0**	8.8	9.7
Sphere (Cs_4PbBr_6), average diameter 15.7 nm (experimental) to Cube ($CsPbBr_3$), average edge length 12 nm (experimental)					
1 equiv. Cs_4PbBr_6 to *n* equiv. of $CsPbBr_3$	n = 1	**n = 2**	-	n = 3	n = 4
Cube edge length, calculated, nm	9.5	**12**	-	13.7	15.1

S14. PL maps of NC samples at room and cryogenic temperatures

Sample preparation: The samples of Cs_4PbBr_6 NCs and a freshly made partially converted Cs_4PbBr_6-$CsPbBr_3$ NCs were drop-casted (10-20 μL drop volume) from toluene solutions on top of a 1”-diameter sapphire disk and dried under ambient atmosphere. After that, the sapphire disk was promptly mounted into the cryostat and placed under vacuum. The time between the preparation (i.e. the moment of mixing of Cs_4PbBr_6 NCs and PMAO) of a partially-converted sample and it’s reaching the lowest temperature (~35K) was around 3 hours.

Cryostat and optical setup: The excitation-emission correlation maps (PL maps) of a partially-converted sample of Cs_4PbBr_6-$CsPbBr_3$ NCs at 292K and 35K (**Figure 4a,b** in the main text) and the as-synthesized Cs_4PbBr_6 NCs (**Figure S43** below) were collected by using a closed-cycle helium cryostat (Advanced Research Systems, Inc., model DE204SI) coupled to FLS920 Edinburgh Instruments spectrofluorimeter via a set of optical fibers. The samples were excited using the output of xenon lamp (Xe900) coupled to a monochromator.

- For the Cs_4PbBr_6-$CsPbBr_3$ NC sample, the PL maps were collected using the following instrumental settings:

Excitation wavelength (y-axis): 300-480 nm range, 3 nm steps, excitation slit width 3 nm,
Emission wavelength (x-axis): 340-550 nm range, 3 nm steps, emission slit width 3 nm,
Dwell time 0.2 s, 3 scans were accumulated at each excitation wavelength.

- For the Cs_4PbBr_6 NC sample, the PL map was collected using the following instrumental settings:

Excitation wavelength (y-axis): 259-349 nm range, 3 nm steps, excitation slit width 3 nm,
Emission wavelength (x-axis): 310-520 nm range, 3 nm steps, emission slit width 3 nm,
Dwell time 0.2 s, 1 scan was accumulated at each excitation wavelength.

The PL maps of the Cs_4PbBr_6-$CsPbBr_3$ NC sample at 292K and 35K were corrected by subtracting the PL maps of a “blank” sample (drop-casted PMAO film) collected at the respective temperatures in a separate experiment under identical instrumental settings. The PL map of the Cs_4PbBr_6 NC sample is shown without correction for a blank.

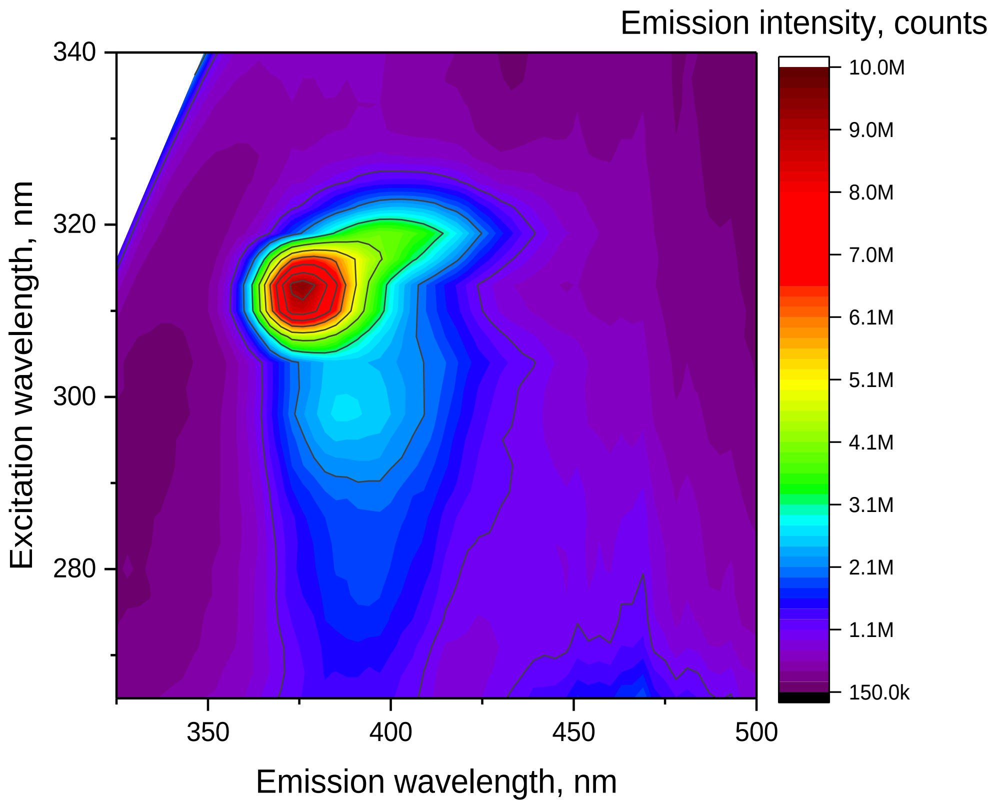

Figure S43. PL map of Cs_4PbBr_6 NCs at T ~27K. The weaker features at around the main emission peak (λ_{exc} ~313 nm, λ_{em} ~373 nm) are attributed to the to various electronic transitions in Pb^{2+} ion.[27-29]

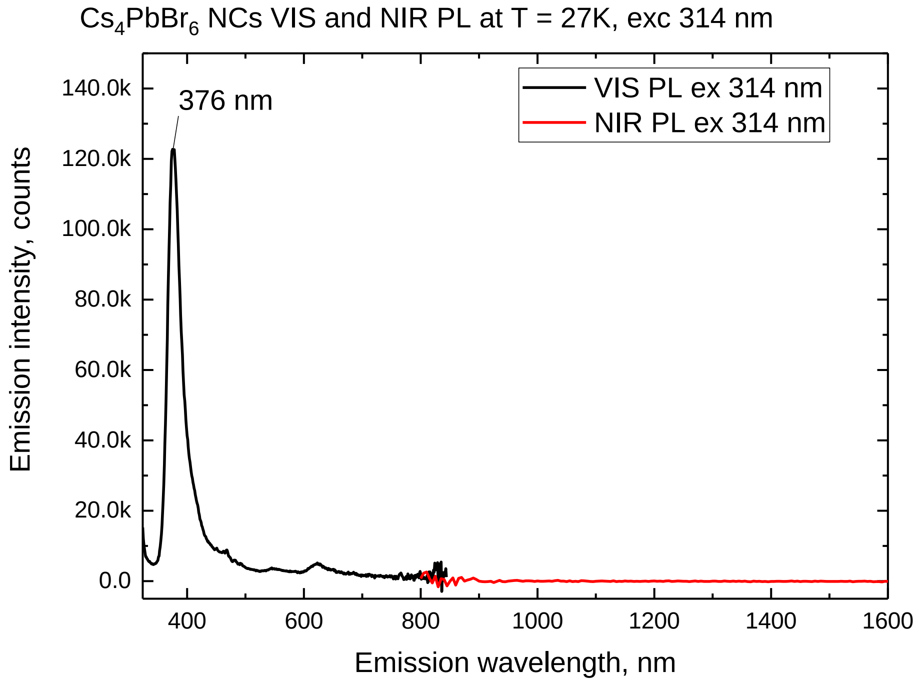

Figure S44. The emission spectrum of Cs_4PbBr_6 NCs at 27K collected over 324-1600 nm range (λ_{exc} ~314 nm). The weak hump at ~618 nm is an artifact from the background. The different colors of the spectra in the visible (VIS PL, black curve) and near-infrared (NIR PL, red curve) regions are due to the different detectors used for each spectral range.

S15. Raman, µ-PL, and TRPL of $CsPbBr_3$/PMAO film

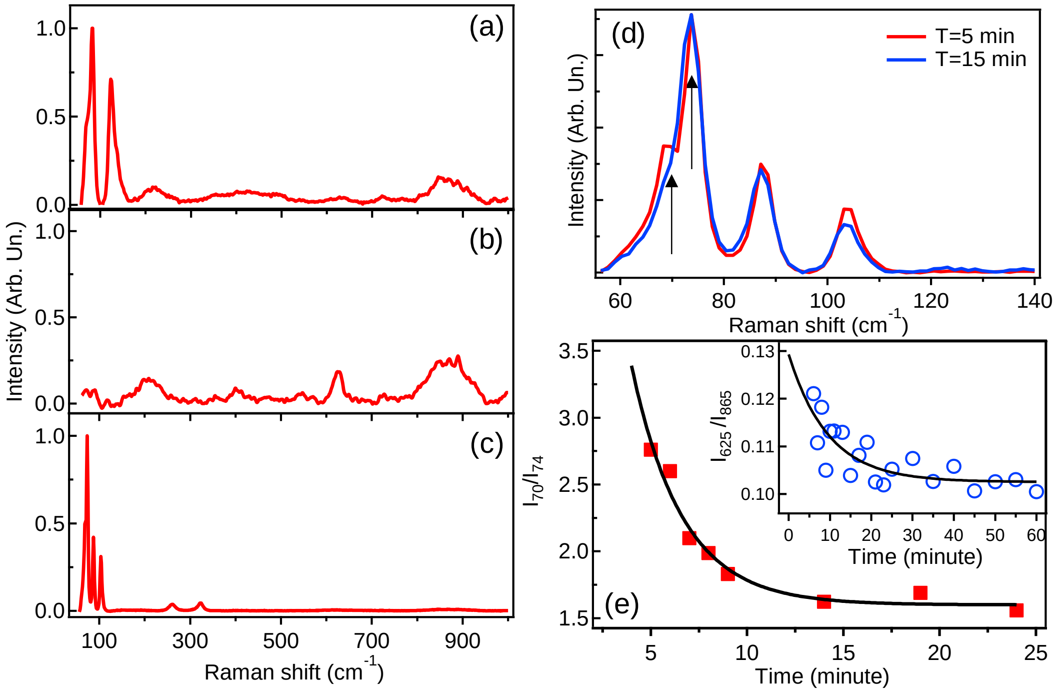

Figure S45. (a) Raman spectra of (a) Cs_4PbBr_6 NCs, (b) PMAO polymer powder and (c) drop-casted film after the complete transformation of Cs_4PbBr_6 into $CsPbBr_3$. (d) Raman spectra of a drop-casted film measured 5 minutes (continuous red line) and 15 minutes (continuous blue line) after the mixing of NCs and PMAO. The vertical arrows highlight the modes at 70 and 74 cm^{-1}, attributed to the Cs_4PbBr_6 and to the $CsPbBr_3$ NCs, respectively. (e) Temporal evolution of the ratio between the intensities of the peak at 70 cm^{-1} (I_{70}) and the peak at 74 cm^{-1} (I_{74}). The continuous line is a fit to the data by first-order kinetics. Inset: temporal evolution of the intensities of the peaks at 625 cm^{-1} (I_{625}) and at 825 cm^{-1} (I_{825}), attributed to the PMAO polymer. The continuous line is a fit to the data by first-order kinetics.

Figure S45a shows the characteristic Raman spectra of the Cs_4PbBr_6 NCs, the PMAO polymer powder (**Figure S45b**), and the composite film after full conversion to $CsPbBr_3$ NCs (**Figure S45c**). One can observe peaks at 625 cm^{-1} (ring bend) and 865 cm^{-1} (C-C stretch), characteristic of the maleic anhydride of the PMAO,[30] and peaks at 70, 82, 123, and 1642 cm^{-1} of the Cs_4PbBr_6. After the reaction, new peaks at 74, 87, 103, 261, and 323 cm^{-1} emerge, which are attributed to the $CsPbBr_3$ NCs.[31] The analysis of the evolution of the Raman spectra during the reaction in the solid-state films evidences the conversion of the Cs_4PbBr_6 NCs to $CsPbBr_3$ NCs, supported by the decrease of the peak at 70 cm^{-1} of the Cs_4PbBr_6 NCs with respect to the peak at 74 cm^{-1} of the $CsPbBr_3$ NCs (**Figure S45d**). This is better highlighted in **Figure S45e**, which displays the ratio of the intensity of those peaks as a function of the reaction time. In fact, the ratio I_{70}/I_{74} (where I_{70} and I_{74} are the intensities of the peaks at 70 and 74 cm^{-1}, respectively) decreases with increasing reaction time, following a trend similar to the one observed in photoluminescence measurements.

Finally, the inset of **Figure S45e** shows the temporal evolution of the peak at 625 cm^{-1}, attributed to the maleic anhydride ring bend, normalized to the peak of the C-C stretch at 865 cm^{-1}, here used a reference. The decrease of the ratio I_{625}/I_{865} (where I_{625} and I_{865} are the intensities of the peaks at 625 and 865 cm^{-1}, respectively) is indicative of a weakening of the modes associated to the anhydride ring, suggesting a reaction path, which might involve the opening of the anhydride ring.

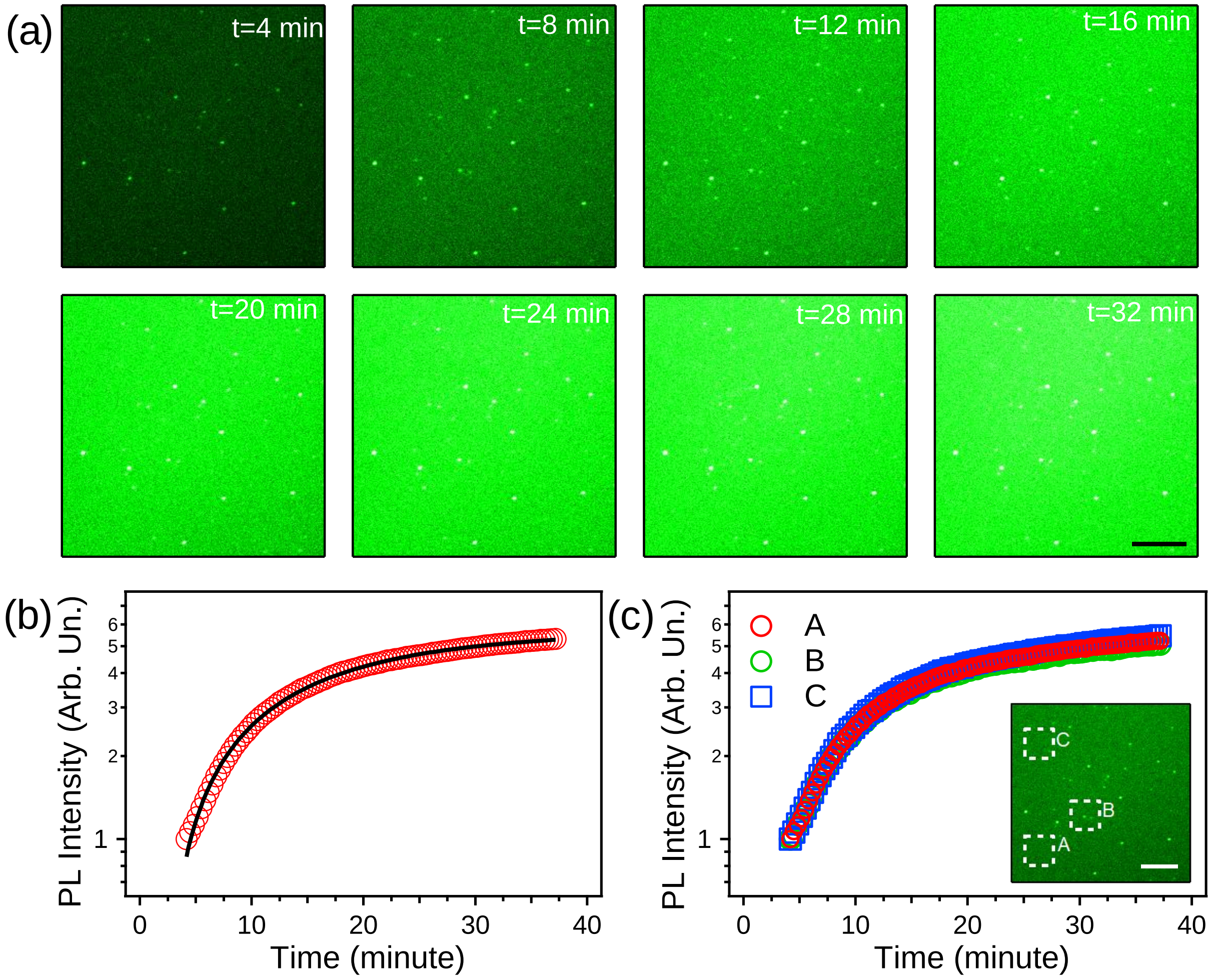

Figure S46. (a) The temporal sequence of fluorescence confocal micrographs measured over the transformation of Cs_4PbBr_6 into $CsPbBr_3$ in drop-casted film. Scale bar: 50 µm. (b) The temporal evolution of the PL intensity averaged over the measured area of the sample. The continuous line is a fit to the data by first-order kinetics. (c) Comparison of the temporal evolution of the PL intensity during the transformation reaction in three different regions of the drop-casted film. The investigated areas are highlighted in the fluorescence maps shown in the inset by dashed squares. Scale bar: 50 µm. t=0 corresponds to the mixing of the NCs and PMAO.

The evolution of the conversion reaction of the Cs_4PbBr_6 NCs to the $CsPbBr_3$ was also investigated by *in-situ* micro-photoluminescence (µ-PL), using a fluorescence confocal microscope. The results are summarized in **Figure S46**. **Figure S46a** shows a sequence of PL

spatial maps (size 250×250 μm^2), clearly evidencing an increase of the sample emission with increasing reaction time. A few bright spots are also observed, which can be attributed to the formation of aggregates. The μ-PL intensity increases following an exponential trend (**Figure S46b**), with a characteristic time of 13 minutes, comparable to the one found for CW and time-resolved macroscopic PL measurements. Interestingly, such a trend is spatially uniform, as shown in **Figure S46c**, where the μ-PL intensity collected in three different regions (labeled as A, B, and C) of the sample are compared.

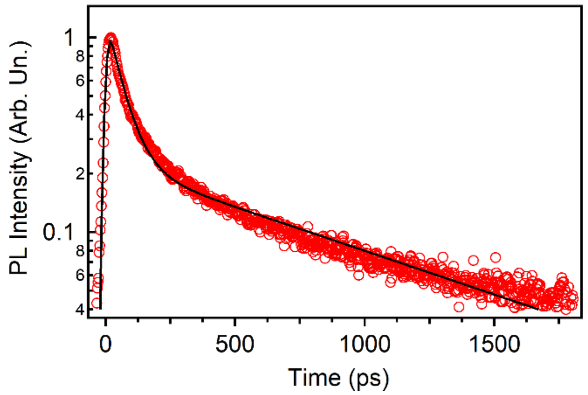

Figure S47. Example of a temporal decay profile of the PL intensity of a drop-casted film after the transformation of Cs_4PbBr_6 into $CsPbBr_3$. Two emitting components are identified, with lifetimes τ_1=60 ps and τ_2=950 ps, as obtained by fitting the data to the sum of two exponential functions, convoluted with the instrumental response function (continuous line).

S16. References

1. Akkerman, Q. A.; Park, S.; Radicchi, E.; Nunzi, F.; Mosconi, E.; De Angelis, F.; Brescia, R.; Rastogi, P.; Prato, M.; Manna, L., Nearly Monodisperse Insulator Cs_4PbX_6 (X = Cl, Br, I) Nanocrystals, Their Mixed Halide Compositions, and Their Transformation into $CsPbX_3$ Nanocrystals. *Nano Lett.* **2017,** 17, (3), 1924-1930.

2. Schneider, C. A.; Rasband, W. S.; Eliceiri, K. W., NIH Image to ImageJ: 25 years of image analysis. *Nat. Methods* **2012,** 9, 671.

3. Gammer, C.; Mangler, C.; Rentenberger, C.; Karnthaler, H. P., Quantitative local profile analysis of nanomaterials by electron diffraction. *Scripta Mater.* **2010,** 63, (3), 312-315.

4. Segets, D.; Lucas, J. M.; Klupp Taylor, R. N.; Scheele, M.; Zheng, H.; Alivisatos, A. P.; Peukert, W., Determination of the Quantum Dot Band Gap Dependence on Particle Size from Optical Absorbance and Transmission Electron Microscopy Measurements. *ACS Nano* **2012,** 6, (10), 9021-9032.

5. Park, S. D.; Baranov, D.; Ryu, J.; Cho, B.; Halder, A.; Seifert, S.; Vajda, S.; Jonas, D. M., Bandgap Inhomogeneity of a PbSe Quantum Dot Ensemble from Two-Dimensional Spectroscopy and Comparison to Size Inhomogeneity from Electron Microscopy. *Nano Lett.* **2017,** 17, (2), 762-771.

6. Toso, S.; Baranov, D.; Giannini, C.; Marras, S.; Manna, L., Wide-Angle X-ray Diffraction Evidence of Structural Coherence in $CsPbBr_3$ Nanocrystal Superlattices. *ACS Mater. Lett.* **2019,** 1, (2), 272-276.

7. de Mello, J. C.; Wittmann, H. F.; Friend, R. H., An improved experimental determination of external photoluminescence quantum efficiency. *Adv. Mater.* **1997,** 9, (3), 230-232.

8. Leff, D. V.; Brandt, L.; Heath, J. R., Synthesis and Characterization of Hydrophobic, Organically-Soluble Gold Nanocrystals Functionalized with Primary Amines. *Langmuir* **1996,** 12, (20), 4723-4730.

9. Hens, Z.; Martins, J. C., A Solution NMR Toolbox for Characterizing the Surface Chemistry of Colloidal Nanocrystals. *Chem. Mater.* **2013,** 25, (8), 1211-1221.

10. De Roo, J.; Ibáñez, M.; Geiregat, P.; Nedelcu, G.; Walravens, W.; Maes, J.; Martins, J. C.; Van Driessche, I.; Kovalenko, M. V.; Hens, Z., Highly Dynamic Ligand Binding and Light Absorption Coefficient of Cesium Lead Bromide Perovskite Nanocrystals. *ACS Nano* **2016,** 10, (2), 2071-2081.

11. De Roo, J.; Yazdani, N.; Drijvers, E.; Lauria, A.; Maes, J.; Owen, J. S.; Van Driessche, I.; Niederberger, M.; Wood, V.; Martins, J. C.; Infante, I.; Hens, Z., Probing Solvent–Ligand Interactions in Colloidal Nanocrystals by the NMR Line Broadening. *Chem. Mater.* **2018,** 30, (15), 5485-5492.

12. Almeida, G.; Goldoni, L.; Akkerman, Q.; Dang, Z.; Khan, A. H.; Marras, S.; Moreels, I.; Manna, L., Role of Acid–Base Equilibria in the Size, Shape, and Phase Control of Cesium Lead Bromide Nanocrystals. *ACS Nano* **2018,** 12, (2), 1704-1711.

13. Velázquez, M.; Ferrier, A.; Péchev, S.; Gravereau, P.; Chaminade, J.-P.; Portier, X.; Moncorgé, R., Growth and characterization of pure and Pr^{3+}-doped Cs_4PbBr_6 crystals. *J. Cryst. Growth* **2008,** 310, (24), 5458-5463.

14. Stoumpos, C. C.; Malliakas, C. D.; Peters, J. A.; Liu, Z.; Sebastian, M.; Im, J.; Chasapis, T. C.; Wibowo, A. C.; Chung, D. Y.; Freeman, A. J.; Wessels, B. W.; Kanatzidis, M. G., Crystal Growth of the Perovskite Semiconductor $CsPbBr_3$: A New Material for High-Energy Radiation Detection. *Cryst. Growth Des.* **2013,** 13, (7), 2722-2727.

15. Ray, A.; Maggioni, D.; Baranov, D.; Dang, Z.; Prato, M.; Akkerman, Q. A.; Goldoni, L.; Caneva, E.; Manna, L.; Abdelhady, A. L., Green-Emitting Powders of Zero-Dimensional Cs_4PbBr_6: Delineating the Intricacies of the Synthesis and the Origin of Photoluminescence. *Chem. Mater.* **2019,** 31, (18), 7761-7769.

16. Demont, A.; Prestipino, C.; Hernandez, O.; Elkaïm, E.; Paofai, S.; Naumov, N.; Fontaine, B.; Gautier, R.; Cordier, S., Unprecedented Electron-Poor Octahedral Ta_6 Clusters in a Solid-State Compound: Synthesis, Characterisations and Theoretical Investigations of $Cs_2BaTa_6Br_{15}O_3$. *Chem. Eur. J.* **2013,** 19, (38), 12711-12719.

17. Zherebetskyy, D.; Scheele, M.; Zhang, Y.; Bronstein, N.; Thompson, C.; Britt, D.; Salmeron, M.; Alivisatos, P.; Wang, L.-W., Hydroxylation of the surface of PbS nanocrystals passivated with oleic acid. *Science* **2014,** 344, (6190), 1380-1384.

18. Grisorio, R.; Debellis, D.; Suranna, G. P.; Gigli, G.; Giansante, C., The Dynamic Organic/Inorganic Interface of Colloidal PbS Quantum Dots. *Angew. Chem. Int. Ed.* **2016,** 55, (23), 6628-6633.

19. Beygi, H.; Sajjadi, S. A.; Babakhani, A.; Young, J. F.; van Veggel, F. C. J. M., Surface chemistry of as-synthesized and air-oxidized PbS quantum dots. *Appl. Surf. Sci.* **2018,** 457, 1-10.

20. Baranov, D.; Lynch, M. J.; Curtis, A. C.; Carollo, A. R.; Douglass, C. R.; Mateo-Tejada, A. M.; Jonas, D. M., Purification of Oleylamine for Materials Synthesis and Spectroscopic Diagnostics for trans Isomers. *Chem. Mater.* **2019,** 31, (4), 1223-1230.

21. Cass, L. C.; Malicki, M.; Weiss, E. A., The Chemical Environments of Oleate Species within Samples of Oleate-Coated PbS Quantum Dots. *Anal. Chem.* **2013,** 85, (14), 6974-6979.

22. McPhail, M. R.; Weiss, E. A., Role of Organosulfur Compounds in the Growth and Final Surface Chemistry of PbS Quantum Dots. *Chem. Mater.* **2014,** 26, (11), 3377-3384.

23. Almeida, G.; Ashton, O. J.; Goldoni, L.; Maggioni, D.; Petralanda, U.; Mishra, N.; Akkerman, Q. A.; Infante, I.; Snaith, H. J.; Manna, L., The Phosphine Oxide Route toward Lead Halide Perovskite Nanocrystals. *J. Am. Chem. Soc.* **2018,** 140, (44), 14878-14886.

24. Rodová, M.; Brožek, J.; Knížek, K.; Nitsch, K., Phase transitions in ternary caesium lead bromide. *J. Therm. Anal. Calorim.* **2003,** 71, (2), 667-673.

25. Brennan, M. C.; Herr, J. E.; Nguyen-Beck, T. S.; Zinna, J.; Draguta, S.; Rouvimov, S.; Parkhill, J.; Kuno, M., Origin of the Size-Dependent Stokes Shift in $CsPbBr_3$ Perovskite Nanocrystals. *J. Am. Chem. Soc.* **2017,** 139, (35), 12201-12208.

26. Maes, J.; Balcaen, L.; Drijvers, E.; Zhao, Q.; De Roo, J.; Vantomme, A.; Vanhaecke, F.; Geiregat, P.; Hens, Z., Light Absorption Coefficient of $CsPbBr_3$ Perovskite Nanocrystals. *J. Phys. Chem. Lett.* **2018,** 9, (11), 3093-3097.

27. Radhakrishna, S.; Pande, K. P., Lead Centers in Cesium Halides. *Phys. Rev. B* **1973,** 7, (1), 424-431.

28. Jacobs, P. W. M., Alkali halide crystals containing impurity ions with the ns^2 ground-state electronic configuration. *J. Phys. Chem. Solids* **1991,** 52, (1), 35-67.

29. Yin, J.; Zhang, Y.; Bruno, A.; Soci, C.; Bakr, O. M.; Brédas, J.-L.; Mohammed, O. F., Intrinsic Lead Ion Emissions in Zero-Dimensional Cs_4PbBr_6 Nanocrystals. *ACS Energy Lett.* **2017,** 2, (12), 2805-2811.

30. Mirone, P.; Chiorboli, P., Infrared and Raman spectra and vibrational assignment of maleic anhydride. *Spectrochim. Acta* **1962,** 18, (11), 1425-1432.

31. Yaffe, O.; Guo, Y.; Tan, L. Z.; Egger, D. A.; Hull, T.; Stoumpos, C. C.; Zheng, F.; Heinz, T. F.; Kronik, L.; Kanatzidis, M. G.; Owen, J. S.; Rappe, A. M.; Pimenta, M. A.; Brus, L. E., Local Polar Fluctuations in Lead Halide Perovskite Crystals. *Phys. Rev. Lett.* **2017,** 118, (13), 136001.
